# HIV-1 vaccine design through minimizing envelope metastability

**DOI:** 10.1126/sciadv.aau6769

**Published:** 2018-11-21

**Authors:** Linling He, Sonu Kumar, Joel D. Allen, Deli Huang, Xiaohe Lin, Colin J. Mann, Karen L. Saye-Francisco, Jeffrey Copps, Anita Sarkar, Gabrielle S. Blizard, Gabriel Ozorowski, Devin Sok, Max Crispin, Andrew B. Ward, David Nemazee, Dennis R. Burton, Ian A. Wilson, Jiang Zhu

**Affiliations:** 1 Department of Immunology and Microbiology, The Scripps Research Institute, La Jolla, CA 92037, USA.; 2 Department of Integrative Structural and Computational Biology, The Scripps Research Institute, La Jolla, CA 92037, USA.; 3 International AIDS Vaccine Initiative Neutralizing Antibody Center and the Collaboration for AIDS Vaccine Discovery, The Scripps Research Institute, La Jolla, CA 92037, USA.; 4 Scripps Center for HIV/AIDS Vaccine Immunology & Immunogen Discovery, The Scripps Research Institute, La Jolla, CA 92037, USA.; 5 Centre for Biological Sciences and Institute for Life Sciences, University of Southampton, Highfield Campus, Southampton SO17 1BJ, UK.; 6 Ragon Institute of Massachusetts General Hospital, Massachusetts Institute of Technology and Harvard, Cambridge, MA 02139-3583, USA.; 7 Skaggs Institute for Chemical Biology, The Scripps Research Institute, La Jolla, CA 92037, USA.

## Abstract

Overcoming envelope metastability is crucial to trimer-based HIV-1 vaccine design. Here, we present a coherent vaccine strategy by minimizing metastability. For 10 strains across five clades, we demonstrate that the gp41 ectodomain (gp41_ECTO_) is the main source of envelope metastability by replacing wild-type gp41_ECTO_ with BG505 gp41_ECTO_ of the uncleaved prefusion-optimized (UFO) design. These gp41_ECTO_-swapped trimers can be produced in CHO cells with high yield and high purity. The crystal structure of a gp41_ECTO_-swapped trimer elucidates how a neutralization-resistant tier 3 virus evades antibody recognition of the V2 apex. UFO trimers of transmitted/founder viruses and UFO trimers containing a consensus-based ancestral gp41_ECTO_ suggest an evolutionary root of metastability. The gp41_ECTO_-stabilized trimers can be readily displayed on 24- and 60-meric nanoparticles, with incorporation of additional T cell help illustrated for a hyperstable 60-mer, I3-01. In mice and rabbits, these gp140 nanoparticles induced tier 2 neutralizing antibody responses more effectively than soluble trimers.

## Teaser

A coherent HIV-1 vaccine strategy addresses envelope stabilization, nanoparticle display, antibody response, and manufacture.

## INTRODUCTION

The envelope glycoprotein (Env) of HIV-1 harbors the epitopes of all broadly neutralizing antibodies (bNAbs) (*1*) and is the main target of vaccine design (*2*). The cleaved, mature Env is presented on HIV-1 virions as a metastable trimer of heterodimers each containing a (co-)receptor-binding protein, gp120, and a transmembrane protein, gp41, which anchors the Env spike within the viral membrane and drives the fusion process during cell entry (*3*). Because of its labile nature and a dense layer of surface glycans (*4*), Env has long resisted structure determination and trimer-based vaccine design efforts. While the functional necessity of a metastable Env for HIV-1 infection is well understood, the molecular source of metastability and how to eliminate it from the Env trimer remain unclear. It is perhaps not an overstatement that overcoming Env metastability is central to trimer-based HIV-1 vaccine design.

Several strategies have been proposed to create stable and soluble gp140 trimers as potential vaccine immunogens. The early generation of gp140 trimers was based on intuitive designs to overcome metastability via deletion of the cleavage site between gp120 and the gp41 ectodomain (gp41_ECTO_) and the addition of trimerization motifs at the C terminus (*5*–*7*). A more rigorous and successful trimer design, designated SOSIP.664, stressed the importance of the gp120/gp41_ECTO_ cleavage, trimer stability, and solubility (*8*). This format uses a disulfide bond to covalently link gp120 and gp41_ECTO_, an I559P mutation in the heptad repeat 1 (HR1) region, and truncation of gp41_ECTO_ at position 664. When applied to clade A BG505, the SOSIP trimer was shown to be a close mimic of the native Env spike (*9*) that enabled Env structure determination for the first time by x-ray crystallography and electron microscopy (EM) (*10*, *11*). The BG505 SOSIP trimer has substantially advanced HIV-1 research by providing an antigen for bNAb isolation and structural characterization (*12*–*23*), a strategy for stabilizing diverse Envs (*24*–*27*), and a template for trimer optimization either to shield non–neutralizing antibody (non-NAb) epitopes or to target bNAb precursors (*28*–*31*). As the SOSIP design continued to evolve, new versions (designated SOSIP.v5 and SOSIP.v6) that further improved trimer stability and immunogenicity were proposed (*32*). Removal of the cleavage site has proven successful in the forms of single-chain gp140 (sc-gp140) (*33*), native flexibly linked (NFL) (*34*, *35*), and uncleaved prefusion-optimized (UFO) (*36*) trimers. However, it was not until recently that the primary cause of Env metastability—an HR1 bend (residues 547 to 569) in gp41_ECTO_—was identified and targeted directly by rational redesign (*36*). Notably, the I559P mutation that improved trimer stability in the SOSIP design is within this HR1 bend (*9*). Glycine substitution in this region was also found to improve trimer properties in the NFL form (*37*). While attempts to elicit tier 2 NAb response with BG505 SOSIP trimers in wild-type (WT) mice were unsuccessful (*38*), both SOSIP and NFL trimers consistently induced autologous tier 2 NAbs in rabbits and nonhuman primates (NHPs), although only sporadic neutralization was observed for heterologous tier 2 isolates (*26*, *39*–*43*). Therefore, despite recent advances in rational design and structural analysis of native-like trimers (*44*, *45*), critical barriers still remain in the path to an effective HIV-1 vaccine.

In this study, we set out to address the diverse challenges in HIV-1 vaccine development with a coherent strategy centered on Env metastability. We first examined the utility of a transient Chinese hamster ovary (CHO) cell line (ExpiCHO) to express native-like trimers, which resulted in superior trimer yield, purity, and antigenicity while displaying subtle differences in glycosylation pattern and B cell response compared to human embryonic kidney (HEK) 293 F–produced trimers. We then demonstrated that gp41_ECTO_ is the main source of metastability by replacing WT gp41_ECTO_ with BG505 gp41_ECTO_ of the UFO design for 10 Envs across clades A, B, C, B/C, and A/E. The gp41_ECTO_-swapped trimers (termed UFO-BG) exhibited substantial yield and purity and were structurally validated by negative-stain EM. Analysis of UFO and UFO-BG trimers by biolayer interferometry (BLI) against a large panel of 19 antibodies provided comprehensive antigenic profiles for each of the 10 Envs tested. A crystal structure was determined for the H078.14 UFO-BG trimer (tier 3, clade B) at a resolution of 4.6 Å, which explains how this neutralization-resistant virus evades apex bNAbs and enables antigenic optimization of this UFO-BG trimer. We also observed high yield, high purity, and native-like antigenicity for the UFO trimers of transmitted/founder (T/F) viruses and the UFO trimers containing a consensus gp41_ECTO_ (termed UFO-C), suggesting an evolutionary explanation of Env metastability. Next, we displayed diverse UFO-BG trimers on a 24-meric ferritin nanoparticle (*46*) and demonstrated how to incorporate T cell help into nanoparticle constructs for a hyperstable 60-mer, I3-01 (*47*). In WT mice, ferritin and I3-01 nanoparticles, as well as a scaffolded gp140 trimer, induced autologous tier 2 NAbs to BG505.T332N after 8 weeks, whereas soluble trimers did not. In rabbits, the ferritin nanoparticle elicited an autologous tier 2 NAb response after 6 weeks, which was statistically different than the tier 2 NAb response elicited by the trimer throughout the course of immunization. Heterologous neutralization was also detected in the analysis of rabbit samples against a 12-virus global panel (*48*). Our study thus presents a coherent strategy for HIV-1 vaccine design as well as vaccine candidates that merit further evaluation in NHPs and potentially in humans.

## RESULTS

### A robust expression system for production of native-like gp140 trimers

Rapid growth in protein therapeutics and vaccines has accelerated the development of high-yield mammalian cell lines (*49*). In the past, trimer designs were primarily characterized in laboratory expression systems, with uncertainties in their transferability to an industrial setting. The CHO cell line is one of the principal mammalian expression systems that meet the Good Manufacturing Practice (GMP) standard. Although SOSIP and NFL trimers have been produced in stable CHO cell lines for in vitro and in vivo testing, gp140 modification and bNAb affinity purification were required to achieve high-quality materials (*42*, *50*–*52*). A transient, high-yield CHO cell line derived from the GMP CHO-S cells, such as ExpiCHO, would likely accelerate the evaluation of new trimer designs and their development toward vaccine candidates.

**Fig. 1:**
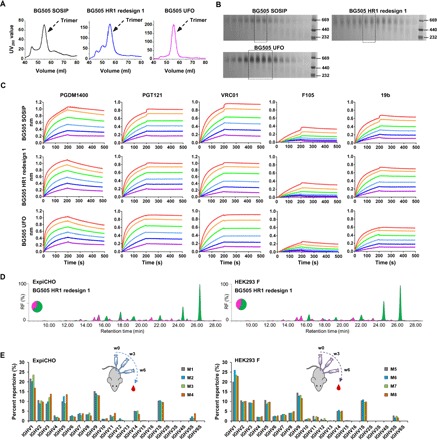
Characterization of ExpiCHO-produced native-like Env trimers. (**A**) SEC profiles of ExpiCHO-expressed, GNL-purified BG505 SOSIP.664 trimer, HR1-redesigned trimer (HR1 redesign 1), and UFO trimer from a Superdex 200 16/600 column. Transient expression in 100-ml ExpiCHO cells was used. (**B**) BN-PAGE of Env proteins for the three aforementioned trimers. The fractions used for antigenic profiling are circled by black dotted lines on the gel. (**C**) Antigenic profiles of the purified trimers measured against a panel of representative bNAbs and non-NAbs, with additional antibody binding profiles shown in fig. S1. Sensorgrams were obtained on an Octet RED96 using a trimer titration series of six concentrations (200 to 6.25 nM by twofold dilution). (**D**) Hydrophilic interaction liquid chromatography (HILIC)–UPLC profiles of the enzymatically released N-linked glycans of the HR1-redesigned trimer produced in ExpiCHO and 293 F cells followed by GNL and SEC purification. Oligomannose-type and hybrid glycans (green) were identified by their sensitivity to endoglycosidase H (Endo H) digestion. Peaks corresponding to complex-type glycans are shown in pink. The peaks are integrated, and the pie charts summarize the quantification of the peak areas. RF, retention factor. (**E**) Heavy-chain germline gene usage of the mouse antibody repertoire primed by ExpiCHO and 293 F–expressed BG505 gp140 trimers containing the HR1 redesign. The percentage of each germline gene family is plotted as a histogram, with a cartoon picture showing the immunization scheme. The results from the four mice in each group are colored in gray (M1, M5), cyan (M2, M6), light green (M3, M7), and orange (M4, M8). w0, week 0; w3, week 3; w6, week 6.

Previously, we reported ExpiCHO expression of gp140-ferritin nanoparticles (*46*). Here, we examined the utility of ExpiCHO for producing native-like trimers. First, we transiently expressed BG505 SOSIP, HR1-redesigned, and UFO gp140 trimers in ExpiCHO cells (all gp140 constructs tested hereafter were truncated at residue 664 unless otherwise stated). Env proteins were extracted from supernatants using a *Galanthus nivalis* lectin (GNL) column and purified by size exclusion chromatography (SEC) on a Superdex 200 16/600 column. Ultraviolet absorbance at 280 nm (UV_280_) was used as a metric to compare the SEC profiles (Fig. 1A). A 100-ml ExpiCHO expression produced well-folded gp140 protein equivalent to that obtained from 2 to 4 liters of 293 F cells (5 to 12 mg before SEC). Overall, we observed a substantial reduction of misfolded species in the Env protein produced by ExpiCHO cells as compared to 293 F cells (*36*). While SOSIP and HR1-redesigned trimers were still mixed with small amounts of aggregates, as shown by the shoulder to the left of the trimer peak at 55 ml, the UFO trimer displayed a single peak indicative of homogeneity. In addition, the UV_280_ value of the UFO trimer was 2.6- and 1.2-fold greater than that of SOSIP and HR1-redesigned trimers, respectively, indicating a higher yield for the UFO trimer. Blue native polyacrylamide gel electrophoresis (BN-PAGE) showed a characteristic trimer band across all SEC fractions with minimal impurity (Fig. 1B). We then evaluated trimer antigenicity using BLI and a panel of six bNAbs and four non-NAbs (Fig. 1C and fig. S1A). While binding kinetics appeared to be largely independent of the cell lines used, trimers produced in ExpiCHO cells showed enhanced bNAb recognition relative to those in 293 F cells (*36*). The UFO trimer displayed the least binding to non-NAbs, consistent with its high purity and stability, although all three trimers bound to a V3-specific non-NAb, 19b.

Next, we investigated the N-glycan processing of the cleaved HR1-redesigned BG505 trimer (*36*) in ExpiCHO and 293 F cells using methods previously reported for the cleaved BG505 SOSIP trimer (*53*, *54*). The oligomannose content of gp140 was quantified using ultrahigh-performance liquid chromatography (UPLC) (Fig. 1D and fig. S1B). Glycans released from 293 F–expressed gp140 consisted of 56% oligomannose type and 44% complex type, while an elevated proportion (64%) of oligomannose-type glycans was observed for the same construct expressed in ExpiCHO cells. The oligomannose content of gp140 expressed in both cell lines appeared to be similar to that observed for the BG505 SOSIP trimer, approximately 63% (*53*) or 68% (*52*). Site-specific glycan analysis was performed using liquid chromatography–mass spectrometry (LC-MS) to determine the relative intensities of the various glycoforms at each N-glycosylation site, revealing features characteristic of the native-like trimers (fig. S1C). As determined by UPLC, many glycosylation sites that present solely oligomannose-type glycans are from regions of underprocessed glycans previously characterized for the SOSIP trimer. For example, glycans at sites N332 and N295 are exclusively oligomannose type (*53*) and are the key elements of a glycan supersite targeted by multiple classes of bNAbs recognizing Man_9_GlcNAc_2_ (*55*). Our analysis thus confirmed that this glycan supersite is present on the HR1-redesigned trimers produced in both cell lines. Similarly, another oligomannose-rich region encompassing N156 and N160 near the trimer apex showed patterns consistent with those observed for the SOSIP trimer (*53*, *54*). Overall, the HR1-redesigned trimers expressed in either 293 F or ExpiCHO cells presented glycosylation patterns consistent with correctly folded Env. However, some ExpiCHO-specific glycan patterns were also observed, such as a lower to nondetectable proportion of complex-type glycans at N339, which may contribute to the enhanced bNAb binding to the N332 supersite (fig. S1D). To examine whether cell line–specific glycan patterns affect trimer-induced B cell responses in vivo, we immunized BALB/c mice with intraperitoneal injections of 50 μg of BG505 gp140 trimer (HR1 redesign 1) adjuvanted with AddaVax at weeks 0, 3, and 6 and then probed the peripheral B cell repertoires by next-generation sequencing (NGS) (*56*). Although the two groups of mice displayed similar patterns of germline gene usage, slightly increased IGHV3 and IGHV5 frequencies (up to 4%) were observed in the B cell repertoires primed by the ExpiCHO-expressed trimer, suggesting a potential glycan influence on trimer-induced B cell response (Fig. 1E).

Together, ExpiCHO provides a robust expression system for producing both native-like trimers and gp140 nanoparticles, with important implications for manufacture. In addition to the proper proteolytic cleavage by furin when coexpressed in ExpiCHO cells, as previously reported for a standard CHO cell line (*51*), subtle differences in glycan processing and the B cell repertoire response are noted between trimers produced in ExpiCHO and 293 F cells.

### Design and characterization of UFO-BG trimers for five HIV-1 clades

A major obstacle faced by current trimer designs is measurable loss in yield, purity, and stability once they are extended from BG505 to other strains. The solutions proposed thus far include the following: (i) purification methods aimed to separate native-like trimers from misfolded and other Env species (monomers, dimers, and aggregates), such as bNAb affinity columns (*24*), negative selection (*34*), multicycle SEC (*33*), and a combined chromatographic approach (*57*), and (ii) auxiliary Env-stabilizing mutations informed by atomic structures (*29*, *37*) or selected from large-scale screening (*31*, *58*). However, these are empirical solutions that may often result in suboptimal outcomes such as reduced trimer yield and bNAb recognition. Recently, we identified an HR1 bend (residues 547 to 569) in gp41_ECTO_ as the primary cause of Env metastability (*36*). Rational redesign of this HR1 bend notably improved trimer yield and purity for multiple HIV-1 strains, yet still produced varying amounts of misfolded Env (*36*). These results suggested that other regions besides HR1, which might be located within gp120 and/or gp41_ECTO_, also contribute to Env metastability. Thus, determining the location of these “secondary factors of metastability” and eliminating them from the Env trimer may prove crucial for trimer-based vaccine design.

**Fig. 2:**
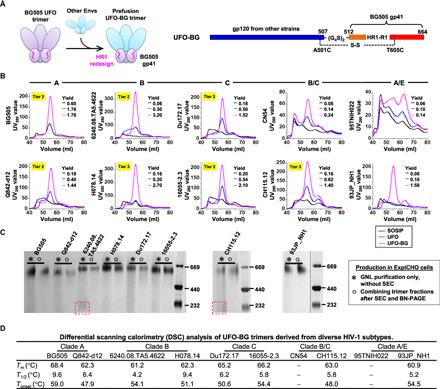
Biochemical and biophysical characterization of the UFO-BG trimers for diverse HIV-1 strains. (**A**) Design (left) and schematic representation (right) of the UFO-BG trimers. As shown on the left, BG505 gp41_ECTO_ of the UFO design is used to stabilize gp120s from other HIV-1 Envs in a hybrid form of gp140 trimer designated UFO-BG. The redesigned HR1 bend is highlighted in magenta. (**B**) SEC profiles of SOSIP, UFO, and UFO-BG trimers derived from 10 Envs across five subtypes (A, B, C, B/C, and A/E) following 100-ml ExpiCHO expression and GNL purification. The yield (in milligrams) of SEC-purified trimer protein (fractions corresponding to 53 to 57 ml) obtained from a 100-ml ExpiCHO expression is listed for each of the three trimer designs (SOSIP, UFO, and UFO-BG). (**C**) BN-PAGE of Env proteins after GNL purification but before SEC and of purified trimers following SEC and BN-PAGE for eight UFO-BG constructs. Two recombinant strains, B/C CN54 and A/E 95TNIH022, were not included due to low purity. (**D**) DSC analysis of eight UFO-BG trimers following GNL and SEC purification. Three thermal parameters (*T*_m_, *T*_1/2_, and *T*_onset_) are listed for each trimer construct.

Here, we hypothesized that all factors of Env metastability are encoded within gp41_ECTO_ and that BG505 gp41_ECTO_ of the UFO design (termed UFO-BG) can be used to stabilize diverse HIV-1 Envs (Fig. 2A). To investigate this hypothesis, we selected 10 Envs across five clades (A, B, C, B/C, and A/E) from either a large panel of tiered HIV-1 pseudoviruses (*59*) or the available database (www.hiv.lanl.gov) and included three Envs tested in our previous study (*36*). Notably, 7 of 10 Envs tested here were derived from tier 2/3 isolates. For each Env, the gp140 constructs of SOSIP, UFO, and UFO-BG designs were transiently expressed in 100-ml ExpiCHO cells, with the SOSIP trimer cotransfected with furin. Following GNL purification, the SEC profiles of 30 gp140s were generated from a Superdex 200 16/600 column for comparison (Fig. 2B). Overall, UFO-BG produced pure trimer protein up to 53- and 5-fold more than SOSIP and UFO, respectively. For all 10 Envs, except for BG505, SOSIPs showed a large proportion of aggregates (at volumes of 40 to 50 ml in the SEC profile) accompanied by an extremely low yield and sometimes the absence of a trimer peak. UFOs showed considerably improved trimer yield and purity, most notably for two clade C strains, although not for clade A/E. UFO-BGs demonstrated unparalleled trimer yield and purity for 8 of 10 strains, with no or only slight hints of dimers and monomers. All 30 gp140 proteins were then characterized by BN-PAGE (fig. S2A). Overall, UFO-BG markedly reduced the dimer and monomer content with respect to SOSIP and UFO, showing a trimer band across all SEC fractions and only occasionally faint bands of lower molecular weight. On the basis of this finding, we compared the total Env protein obtained from a GNL column against the pooled trimer protein after SEC and fraction analysis. GNL purification alone yielded comparable purity for all UFO-BG trimers, except for those derived from a tier 2 clade B strain and a tier 3 clade B/C strain (Fig. 2C). Next, thermal stability was assessed for eight purified UFO-BG trimers using differential scanning calorimetry (DSC) (Fig. 2D and fig. S2B). Notably, the DSC profiles exhibited a clade- or strain-specific pattern, with the thermal denaturation midpoint (*T*_m_) ranging from 60.9° to 68.4°C. Among the eight UFO-BG trimers tested, BG505 displayed the highest *T*_m_ (68.4°C), which was followed by two clade C trimers (65.2° to 66.2°C). The DSC data largely reflected the thermal stability of gp41_ECTO_-stabilized Envs in the absence of additional disulfide bonds and cavity-filling mutations. Additional DSC data for selected constructs revealed that the further enhanced thermal stability of UFO-BG trimers was a result of gp41_ECTO_ swapping (fig. S2C). The expression system had negligible effect on Env thermal stability, as ExpiCHO- and 293 F–expressed trimers showed almost identical *T*_m_ values (fig. S2D). It should be noted that the CN54 UFO and UFO-BG constructs contained 14 mutations (CN54M14), which reduced aggregates for 293 F–produced trimers (fig. S2E). Four UFO-BG trimers were randomly selected from clades B, C, and B/C for expression in 293 F cells and SEC characterization (fig. S2F). UFO-BG was found to improve trimer properties regardless of the expression system but achieved optimal purity when produced in ExpiCHO cells.

Our results thus confirm that gp41_ECTO_ is the primary source of metastability and BG505 gp41_ECTO_ of the UFO design can be used to stabilize diverse Envs. Notably, Env stabilization by BG505 gp41_ECTO_ of the SOSIP design was recently reported (*60*, *61*), but the trigger of Env metastability—the HR1 bend (*36*)—is still present in the resulting trimers. For UFO-BG trimers, the similar purity before and after SEC suggests that a simple and cost-effective manufacturing solution can be achieved. The inherent high purity of UFO-BG trimers should also accelerate the development and clinical testing of nucleic acid vaccine strategies (*62*–*64*).

### Structural characterization of the UFO-BG trimers

**Fig. 3:**
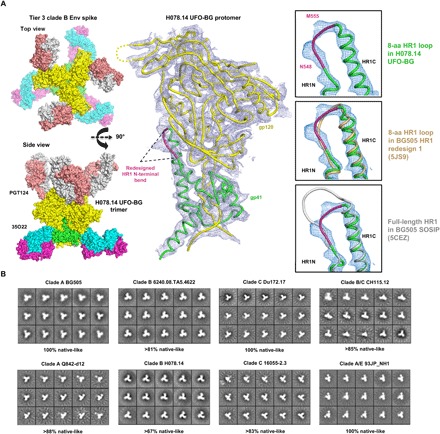
Structural characterization of the UFO-BG trimers derived from diverse HIV-1 Envs. (**A**) Crystal structure of a clade B tier 3 H078.14 Env spike determined at a resolution of 4.6 Å. Molecular surface of the H078.14 UFO-BG trimer in complex with bNAb Fabs PGT124 and 35O22 is shown on the left (top view and side view), with a ribbon model of the gp140 protomer and two Fabs shown in the middle, and a zoomed-in view of the redesigned HR1 bend (alone and superimposed onto two available structures, 5JS9 and 5CEZ) on the right. (**B**) Reference-free 2D class averages derived from negative-stain EM of eight UFO-BG trimers produced in ExpiCHO cells followed by GNL and SEC purification, with the full sets of images shown in fig. S3B. The percentage of native-like trimers is indicated for each trimer construct. aa, amino acid.

While biochemical and biophysical properties are informative, structures would provide the most convincing evidence that the UFO-BG trimers are an accurate mimic of the native Env (*45*). The clade B H078.14 gp140 construct was selected for crystallization screening, as the trimer structure for a tier 3 neutralization-resistant isolate had yet to be determined. Briefly, this UFO-BG trimer was produced in 293 F cells with kifunensine treatment to inhibit the formation of complex-type glycans and then purified with a 2G12 affinity column followed by SEC on a Superdex 200 16/600 column. Cocrystallization with antigen-binding fragments (Fabs) of bNAbs PGT124 and 35O22 resulted in a complex structure at a resolution of 4.6 Å (Fig. 3A, left, and fig. S3A). Overall, the H078.14 UFO-BG trimer adopts a native-like Env conformation closely resembling that of BG505 SOSIP [Protein Data Bank (PDB): 5CEZ, 3.03 Å] and HR1-redesigned (PDB: 5JS9, 6.92 Å) trimers (*22*, *36*), with global C_α_ root mean square deviations of 0.36 and 1.09 Å, respectively (Fig. 3A, middle). Small differences were noted at the HR1 bend and the first turn of HR1 central C-terminal helix after structural superposition of gp41_ECTO_ (Fig. 3A, right). Crystallographic analysis at this moderate resolution thus confirmed that BG505 gp41_ECTO_ of the UFO design, with a minimal level of metastability, can be used to stabilize diverse HIV-1 Envs in a prefusion state, in addition to providing the first structural model for a tier 3 neutralization-resistant Env spike. Negative-stain EM was used to characterize eight UFO-BG trimers that showed substantial purity in SEC and BN-PAGE (Fig. 2, B and C). As indicated by the two-dimensional (2D) class averages, 67 to 100% of GNL-purified Env protein appeared to be native-like trimers (Fig. 3B and fig. S3B). Similar results were reported for the SOSIP trimers only after purification using bNAb affinity columns (*9*, *24*–*26*, *32*, *65*). To summarize, crystallographic and EM analyses validated the structural integrity of UFO-BG trimers derived from five subtypes, supporting the notion that UFO-BG is a general and effective strategy for trimer stabilization.

### Antigenic evaluation of UFO and UFO-BG trimers

**Fig. 4:**
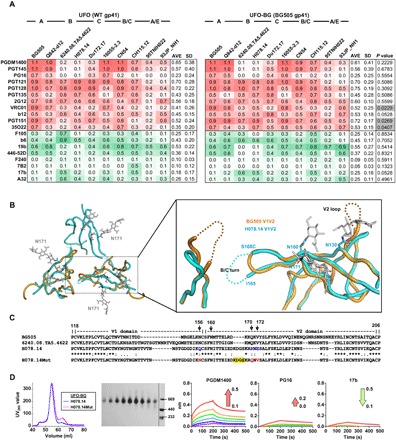
Antigenic map of diverse HIV-1 strains and structure-informed optimization of the H078.14 UFO-BG trimer. (**A**) Antigenic profiles of 10 UFO trimers (left) and 10 UFO-BG trimers (right) against 11 bNAbs and 8 non-NAbs. Sensorgrams were obtained from an Octet RED96 using a trimer titration series of six concentrations (200 to 6.25 nM by twofold dilution) and are shown in fig. S4. The peak values at the highest concentration are summarized in the matrix, in which cells are colored in red and green for bNAbs and non-NAbs, respectively. Higher color intensity indicates greater binding signal measured by Octet. To facilitate antigenic comparison between UFO and UFO-BG trimers, the average peak value (AVE) and SD are listed for each antibody in the two matrices. *P* values calculated from paired *t* test are listed in the last column of the UFO-BG matrix, with statistically significant *P* values (<0.05) highlighted in gray. (**B**) Top-down view of the H078.14 UFO-BG trimer apex and zoomed-in view of the H078.14 V1V2 apex superposed with that of the BG505 SOSIP.664 trimer (PDB: 5CEZ). Glycans at N130, N160, and N171 are labeled for H078.14. The turn between strands B and C of H078.14 and the V2 loop of BG505 are shown as dotted lines in blue and orange, respectively. (**C**) Sequence alignment of V1V2 regions from BG505, 6240.08.TA5.4622 (clade B), WT H078.14 (clade B), and a modified H078.14 (termed H078.14Mut) with mutations at positions 156, 170, and 172 colored in red and “KDGS” deletion at the turn of strands B and C highlighted in yellow. (**D**) Characterization of an H078.14Mut construct that also contains a disulfide bond (I201C-A433C) to prevent CD4-induced conformational changes. Trimers produced in 100-ml ExpiCHO cells are characterized by SEC (left), BN-PAGE (middle), and antigenic evaluation against the V2 apex–directed bNAbs PGDM1400 and PG16 and a CD4i-specific non-NAb 17b (right). The direction and magnitude of the change of peak binding signal (in nanometers) are labeled on the sensorgrams of the H078.14Mut UFO-BG trimer, with an arrow colored in red and green for bNAbs and non-NAbs, respectively.

Following structural characterization, the effect of gp41_ECTO_ substitution on trimer antigenicity was assessed by BLI (Fig. 4A and fig. S4). To this end, we compared the UFO-BG trimers to the UFO trimers containing WT gp41_ECTO_ and a generic GS linker at the HR1 bend (*36*). Following GNL and SEC purification, trimer proteins were tested for antibody binding on Octet RED96, as previously described (*36*). A panel of 11 bNAbs was used to assess conserved neutralizing epitopes on the trimer surface, including the V2 apex recognized by PGDM1400 (*12*), PGT145 (*55*), and PG16 (*66*); the N332 supersite recognized by PGT121, PGT128, PGT135 (*55*), and 2G12 (*67*); the CD4-binding site (CD4bs) recognized by VRC01 (*68*) and b12 (*69*); and the gp120-gp41 interface recognized by PGT151 (*14*) and 35O22 (*13*), along with eight non-NAbs targeting the CD4bs, the CD4-induced (CD4i) epitope, and the immunodominant epitopes at the V3 tip and within gp41_ECTO_.

UFO and UFO-BG trimers derived from 10 strains of five subtypes, 20 in total, were assessed against 19 antibodies in 380 Octet experiments (fig. S4, A to J). The peak antibody-binding signals, as well as the average and standard deviation (SD) for each antibody, were summarized in two matrices corresponding to UFO and UFO-BG trimers, providing by far the most complete antigenic profiles for these HIV-1 subtypes (Fig. 4A). Overall, both UFO trimer designs exhibited largely similar antigenic properties with clade-specific patterns. Notably, trimers derived from clade B 6240.08.TA5.4622 and H078.14 were poorly recognized by apex-directed bNAbs while shielding the immunodominant V3 and gp41 epitopes more effectively than trimers of other clades. However, this reduced non-NAb recognition of the distal V3 and gp41 epitopes was accompanied by enhanced non-NAb binding to the CD4bs and the CD4i epitope, suggesting localized antigenic features specific to these two clade B Envs. The trimers derived from A/E-recombinant strains displayed similar antigenic patterns, with relatively weak binding to most of the antibodies tested. Notably, the substitution of WT gp41_ECTO_ with BG505 gp41_ECTO_ of the UFO design was found to significantly improve trimer binding to bNAbs VRC01, PGT151, and 35O22, with *P* values (paired *t* test) of 0.0229, 0.0269, and 0.0407, respectively. This improved bNAb recognition was likely due to a more stable gp120 conformation (for VRC01) and a restored quaternary epitope at the gp120-gp41 interface (for PGT151 and 35O22). However, this gp41_ECTO_ swapping exerted a more complicated effect on other bNAb epitopes, causing small variations in peak signal and, in some cases, binding kinetics. For example, for clade A tier 2 Q842-d12, the UFO-BG trimer bound to PGDM1400 and PG16 with a faster association rate than the UFO trimer, whereas the B/C-recombinant CN54 trimer showed a decreased on-rate in PGDM1400 and PGT145 binding after gp41_ECTO_ swapping (fig. S4, B and G). For five selected Envs, we also assessed CD4 binding to SOSIP, UFO, and UFO-BG trimers using BLI and CD4-Ig (immunoglobulin), revealing a strain-specific rather than a design-specific pattern (fig. S4K). Nonetheless, systematic antigenic profiling by BLI confirmed that UFO-BG trimers present a close antigenic mimic of the native Env.

### Structure-informed optimization of a tier 3 clade B UFO-BG trimer

Even with a medium-resolution structure (Fig. 3A), the H078.14 UFO-BG trimer provides a valuable template for vaccine design, as it only bound to three of eight non-NAbs (Fig. 4A). However, it is imperative to first determine the cause of poor bNAb binding to the V2 apex, which appeared to be inconsistent with the native-like prefusion trimer conformation. To this end, we superposed the apices of H078.14 UFO-BG and BG505 SOSIP trimer structures (*22*) for visual inspection, which revealed a short insertion at the tip of the V2 hairpin, an additional N-linked glycan at position N171 (HXB2 numbering), and a shortened V2 loop (Fig. 4B). On the basis of the sequence alignment and the crystal structure, we identified two more residues in strand C that may have destabilized the V2 apex. Specifically, the inward-facing Q170 and V172 in BG505 are now replaced with charged bulky residues R170 and E172 in H078.14 (Fig. 4C). On the basis of this information, we sought to optimize the H078.14 UFO-BG trimer by restoring the V2 apex with a triple mutation in strand C (Q156N/R170Q/E172V, Q156N to restore this N-glycosylation site) and a deletion at the tip of the V2 hairpin (ΔKDGS) and by blocking the CD4 binding–induced conformational change with an I201C-A433C disulfide bond (*28*, *35*). As shown in SEC and BN-PAGE, the modified H078.14 UFO-BG trimer retained the same level of yield and purity as the original construct (Fig. 4D, left and middle). In BLI assays, this trimer was well recognized by PGDM1400, but still less effectively by PG16, while showing notably reduced binding to a CD4i non-NAb, 17b (Fig. 4D, right). Our analysis thus confirmed that H078.14 can evade apex bNAbs through mutations in strand C and surrounding loops. The additional glycan at N171 may have little effect on apex bNAb binding as it points sideways in the crystal structure (Fig. 4B, left). However, we noted that clade B 6240.08.TA5.4622, which also showed poor trimer binding to apex bNAbs, has the same amino acids as BG505 at those positions critical to H078.14 (Fig. 4C), suggesting that this tier 2 virus must use a different mechanism to shield its apex.

### Envelope metastability has an evolutionary root

The genetic diversity of HIV-1 has been extensively studied (*70*) and is considered a crucial factor for vaccine design (*71*, *72*). Envs obtained from T/F viruses or derived from a sequence database by phylogeny, which both represent ancestral states of HIV-1, have been evaluated as vaccine immunogens (*73*–*75*). The T/F viruses have been found to exhibit greater infectivity relative to chronic viruses (*76*). Considering that BG505 is a T/F virus (*77*) and that BG505 gp41_ECTO_ of the UFO design can stabilize diverse Envs, we hypothesized that the superior infectivity of T/F viruses could be a result of greater gp41_ECTO_ stability, and hence, the T/F UFO trimers might exhibit higher yield, purity, and stability (fig. S5A). To explore this hypothesis, we tested UFO and UFO-BG trimers for three T/F strains: clade B B41 (*24*, *40*, *58*), clade C CH505 (*78*–*80*), and clade C 1086 (*37*, *73*). Following 100-ml ExpiCHO expression and GNL purification, the Env protein was characterized by SEC (fig. S5B). For clade B T/F B41, UFO and UFO-BG trimers showed similar purity, with a greater yield observed for UFO-BG. For two clade C T/F Envs, a considerably high trimer yield was observed for the UFO-BG design. B41 UFO and CH505 UFO-BG were then assessed by BLI using a small panel of antibodies, both displaying antigenic profiles consistent with native-like trimers (fig. S5C). In a recent study, Sullivan *et al*. (*58*) screened 852 mutations to improve the antigenicity and stability of a B41 SOSIP trimer. In another study, Guenaga *et al*. (*37*) screened various glycine substitutions in the HR1 bend in addition to other stabilizing mutations to improve the 1086 NFL trimer. Our results suggest that UFO and UFO-BG may provide a simple and effective alternative to the large-scale screening-based approaches for engineering native-like T/F trimers.

We next explored the UFO design using a consensus gp41_ECTO_ derived from the available Env sequences in the database, designated UFO-C (fig. S5D). If such a UFO-C design is proven successful, then it will provide further evidence for the evolutionary root of metastability, as consensus has been considered a simple approximation to the ancestral state in HIV-1 evolution (*72*, *81*, *82*). To this end, we derived a consensus gp41_ECTO_ from 6670 full-length Env sequences (www.hiv.lanl.gov/) with the HR1 bend replaced by a generic HR1 linker, as reported in our previous study (*36*). The UFO-C constructs were created for five Envs of different subtypes and characterized by SEC following ExpiCHO expression and GNL purification (fig. S5E). Overall, UFO-C outperformed SOSIP and UFO for five and three Envs, respectively, displaying improved trimer yield and purity. For clade A BG505, UFO-C exhibited a SEC profile similar to that of UFO but with slightly increased aggregates and decreased trimer yield. This result confirmed that consensus gp41_ECTO_ can recapitulate, in large part, the inherent features of BG505 gp41_ECTO_. UFO-C appeared to be least effective for a tier 3 B/C-recombinant strain, CH115.12, for which UFO-BG was also less successful than for other Envs (Fig. 2B). In terms of thermal stability, UFO-C trimers showed a reduction of *T*_m_ in the range of 3.5° to 5.5°C compared to their respective UFO-BG trimers (fig. S5F). UFO-C trimers derived from clade A and B Envs were further validated by BN-PAGE and showed no difference in purity before and after SEC (fig. S5G). Consistently, negative-stain EM confirmed that more than 90% of UFO-C trimers were native like (fig. S5H). Last, BLI was used to assess the antigenicity of five UFO-C trimers (fig. S5I). In general, UFO-C exhibited antigenic profiles on par with UFO-BG for Envs of clades A and B but not of others. Together, the results suggest that consensus gp41_ECTO_ is a promising design but will require further optimization to achieve the same level of stability as BG505 gp41_ECTO_.

### Nanoparticle presentation of UFO-BG trimers derived from diverse subtypes

**Fig. 5:**
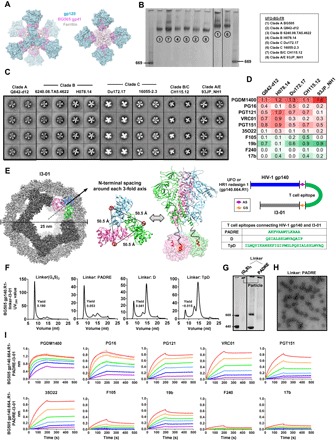
Ferritin nanoparticles presenting diverse UFO-BG trimers and I3-01–based gp140 nanoparticles with embedded T cell help signal. (**A**) Surface model of UFO-BG gp140-ferritin nanoparticle, with gp120, BG505 gp41_ECTO_ of the UFO design, and ferritin colored in cyan, magenta, and gray, respectively. (**B**) BN-PAGE of eight UFO-BG-FR nanoparticles after a single-step 2G12 affinity purification. (**C**) Reference-free 2D class averages derived from negative-stain EM of five representative UFO-BG-FR nanoparticles. (**D**) Antigenic profiles of five representative UFO-BG-FR nanoparticles against six bNAbs and four non-NAbs. Sensorgrams were obtained on an Octet RED96 using a trimer titration series of six concentrations (starting at 35 nM by twofold dilution) and are shown in fig. S6. The peak values at the highest concentration are summarized in the matrix, in which cells are colored in red and green for bNAbs and non-NAbs, respectively. Higher color intensity indicates greater binding signal measured by Octet. (**E**) Left: Surface model of the I3-01 nanoparticle (colored in gray), with the subunits surrounding a front-facing fivefold axis highlighted in dark gray and three subunits forming a threefold axis colored in sky blue, magenta, and green, respectively. Middle: Spacing between N termini of three I3-01 subunits surrounding a threefold axis (top view) and the anchoring of a gp140 trimer onto three I3-01 subunits by flexible peptide linkers (indicated by black dotted lines). Right: Schematic representation of I3-01 nanoparticle constructs containing both gp140 and a T helper epitope, with sequences listed for three such T helper epitopes: PADRE, D, and TpD. (**F**) SEC profiles of I3-01 nanoparticles presenting an HR1-redesigned BG505 trimer (termed gp140.664.R1) with a 10–amino acid GS linker (left) and three T helper epitope linkers (right). The yields (in milligrams) of nanoparticle protein obtained from a 100-ml ExpiCHO expression and after 2G12 and SEC purification are labeled on the SEC profiles. (**G**) BN-PAGE of two I3-01 nanoparticles, containing a GS linker and a T helper epitope linker (PADRE), after a single-step 2G12 affinity purification. (**H**) Micrograph derived from negative-stain EM of 2G12-purified I3-01 nanoparticle presenting an HR1-redesigned BG505 trimer with a PADRE linker (termed gp140.664.R1-PADRE-I3-01). (**I**) Antigenic profiles of BG505 gp140.664.R1-PADRE-I3-01 nanoparticle against six bNAbs and four non-NAbs. Sensorgrams were obtained on an Octet RED96 using a trimer titration series of six concentrations (starting at 14 nM by twofold dilution).

It has been well established that nanoparticle display of antigens elicits stronger immune responses than non-arrayed antigens (*83*–*86*). However, creating trimer-presenting nanoparticles by the gene fusion approach has proven difficult and was only reported for clade A BG505 (*46*, *87*). On the surface of these gp140 nanoparticles, gp41_ECTO_ would form a “neck” region that connects the gp140 trimer and the nanoparticle backbone beneath. Here, we hypothesized that BG505 gp41_ECTO_ of the UFO design can facilitate both gp140 trimerization and nanoparticle assembly (Fig. 5A). To validate this hypothesis, we displayed eight UFO-BG trimers of five subtypes on a ferritin (FR) nanoparticle, which was previously used to present an HR1-resesigned BG505 trimer (*46*). Briefly, UFO-BG-FR constructs were designed by fusing the C terminus of gp41_ECTO_ (residue 664) to the N terminus (Asp5) of a ferritin subunit. These constructs were expressed transiently in 100-ml ExpiCHO cells followed by a single-step purification with a 2G12 affinity column. BN-PAGE displayed a distinctive band of high molecular weight corresponding to well-formed UFO-BG-FR nanoparticles for all eight strains (Fig. 5B). Nanoparticle assembly was further confirmed by negative-stain EM, showing a visible core decorated with eight trimer spikes protruding from the nanoparticle surface (Fig. 5C and fig. S6A). The UFO-BG-FR nanoparticles exhibited greater thermal stability than the respective UFO-BG trimers, with *T*_m_ ranging from 68° to 70°C (fig. S6B). The antigenicity was assessed for five representative UFO-BG-FR nanoparticles using six bNAbs and four non-NAbs. Overall, particulate display retained, and in some cases enhanced, the native-like trimer antigenicity, showing patterns specific to epitopes as well as HIV-1 subtypes (Fig. 5D and fig. S6C). For the V2 apex, PGDM1400 bound to all nanoparticles with comparable or notably higher affinity than the corresponding trimers (Fig. 4A), suggesting that the displayed trimers have native-like, closed conformations. For clade B tier 3 H078.14, the restored binding to apex bNAbs might be explained by the enhanced stability of V2 hairpin due to molecular crowding in the presence of neighboring trimers on the nanoparticle surface (*88*), whereas for Du172.17 and 93JP_NH1, the increased affinity for apex bNAbs was likely a result of avidity. For the N332 supersite and the CD4bs, particulate display exerted a more favorable influence on the H078.14 UFO-BG trimer. For the gp120-gp41 interface, while all UFO-BG-FR nanoparticles retained the trimer binding to PGT151 (*15*), a cross-clade reduction in 35O22 binding was observed due to the constrained angle of approach (*13*) on the ferritin nanoparticle surface. For non-NAbs, UFO-BG-FR nanoparticles displayed similar antigenic profiles to respective UFO-BG trimers, with slightly reduced binding to non-NAbs except for 19b. Our results suggest that UFO-BG trimers of diverse subtypes can be readily displayed on nanoparticles due to their enhanced gp41_ECTO_ stability, thus providing an effective approach for designing heterologous nanoparticle vaccines.

### Design of trimer-presenting nanoparticles with built-in T cell help

Previously, we designed and characterized gp120 and gp140 nanoparticles based on a large 60-mer, E2p (*46*). Recently, Hsia *et al*. (*47*) reported a hyperstable 60-mer (I3-01) resistant to guanidine hydrochloride at high temperature. Our database search identified a bacterial enzyme from *Thermotoga maritima* with only five residues differing from I3-01 that has been crystallized at a resolution of 2.3 Å (PDB: 1VLW) (fig. S6D). Here, we examined the utility of I3-01 for designing gp140 nanoparticles. In terms of symmetry (dodecahedron) and size (25 nm), I3-01 (Fig. 5E, left) closely resembles E2p (*46*). However, the large spacing between the N termini of I3-01 subunits (~50.5 Å) requires a long linker to connect with the C termini of the gp140 trimer (29.1 Å) (Fig. 5E, middle). We thus hypothesized that a helper T cell epitope may be used not only as a linker between gp140 and I3-01 but also as an embedded signal to boost T cell response and to accelerate Env-specific B cell development toward bNAbs (*89*). To explore this possibility, we designed three constructs, each containing an HR1-redesigned BG505 gp140 (termed gp140.664.R1) (*36*), one of the three selected T cell epitopes [PADRE (*90*), D, and TpD (*91*)], and an I3-01 subunit (Fig. 5E, right). A fourth construct containing a 10–amino acid (G_4_S)_2_ linker was included for comparison. Following furin coexpression in ExpiCHO cells, the 2G12-purified protein was characterized by SEC (Fig. 5F). The 10–amino acid GS linker resulted in I3-01 nanoparticles of high yield and purity, whereas the three T cell epitopes appeared to affect nanoparticle assembly to various extents due to their hydrophobic nature. Of the three T cell epitopes, PADRE produced nanoparticles of the highest purity, as indicated by SEC (Fig. 5F), BN-PAGE (Fig. 5G), and negative-stain EM (Fig. 5H). In BLI assays, the gp140.664.R1-PADRE-I3-01 nanoparticle exhibited a desirable antigenic profile with strong bNAb binding and minimal non-NAb binding (Fig. 5I). To probe the stability of this nanoparticle, we designed 10 variants based on the original gene of I3-01, 1VLW (fig. S6E). The SEC profiles revealed the importance of a hydrophobic patch at the dimeric interface that facilitates nanoparticle assembly (fig. S6F). Together, we have reengineered a hyperstable nanoparticle to display 20 gp41_ECTO_-stabilized trimers on the surface with a built-in T cell help signal.

### Nanoparticles potently activate B cells expressing bNAbs

**Fig. 6:**
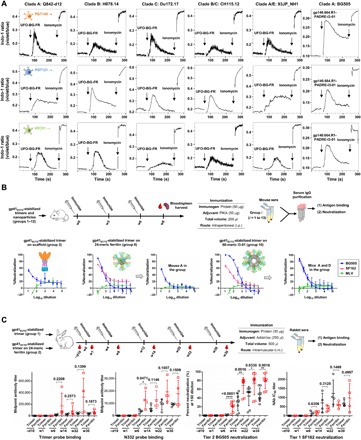
Evaluation of trimers and nanoparticles in B cell activation assays and in two small-animal models. (**A**) Ca^2+^ mobilization by various gp140 nanoparticles in B cell transfectants carrying PGT145, PGT121, and VRC01 bNAb receptors. WEHI231 cells expressing a doxycycline-inducible form of bNAb BCR were stimulated with anti-BCR antibodies or the indicated antigens at a concentration of 10 μg ml^−1^: anti-human Ig κ-chain F(ab′)_2_; anti-mouse IgM; an UFO-BG-FR nanoparticle derived from a clade A, B, C, B/C, or A/E strain; or a BG505 gp140-PADRE-I3-01 nanoparticle containing a redesigned HR1 bend within gp41_ECTO_. (**B**) Top: Assessment of immunogenicity in WT mice. Schematic representation of the mouse immunization protocol. Bottom: Neutralization curves for groups 3, 6, and 10, which correspond to a scaffolded full-length gp140 trimer (gp140.681.R1-1NOG), a gp140-ferritin nanoparticle (gp140.664.R1-FR), and a gp140-I3-01 nanoparticle with T cell help (gp140.664.R1-PADRE-I3-01), respectively. Structural models of these three immunogens are placed next to their group-combined neutralization curves. The neutralization curves are also included for individual mice whose serum IgGs neutralized BG505.T332N. (**C**) Assessment of immunogenicity in rabbits. Schematic representation of the rabbit immunization protocol (top), longitudinal analysis of midpoint titers of antibodies reactive with the HR1-redesigned BG505 trimer (gp140.664.R1) and an N332 nanoparticle probe (bottom, left), and longitudinal analysis of neutralization against autologous tier 2 BG505.T332N and clade B tier 1 SF162 (bottom, right). Percent neutralization (%) at the 50-fold plasma dilution and ID_50_ (50% inhibitory dose) are plotted for BG505 and SF162, respectively. An unpaired *t* test was performed to determine whether trimer and ferritin groups were significantly different (*P* < 0.05) in plasma binding and neutralization. *P* values are shown for time points w6, w14, w22, and w30, with asterisks indicating the level of statistical significance. *P ≤ 0.05, **P ≤ 0.01, ***P ≤ 0.001, ****P ≤ 0.0001. Detailed plasma ELISA and neutralization curves are shown in fig. S7, D and E. −d10, day −10.

Previously, we demonstrated that various BG505 gp120 and gp140 nanoparticles could engage B cells expressing cognate VRC01 receptors (*46*). Here, we assessed the degree of B cell activation by five UFO-BG-FR nanoparticles and a BG505 gp140.664.R1-PADRE-I3-01 nanoparticle with respect to trimers (Fig. 6A and fig. S7A). B cell lines expressing bNAbs PGT145, VRC01, and PGT121 (*92*) were used in this assay. Overall, nanoparticles stimulated bNAb-expressing B cells more effectively than trimers, with peak signals approaching the maximal activation by ionomycin. However, the results also revealed an epitope-dependent pattern: When tested in B cells expressing bNAb PGT121, which recognize the N332 supersite, some trimers and all nanoparticles rendered detectable Ca^2+^ flux signals; in contrast, none and few trimers activated B cells expressing PGT145 and VRC01, which target the V2 apex and the CD4bs, respectively. The stimulation of PGT145-expressing B cells by H078.14 UFO-BG-FR provides further evidence that the apex can be stabilized by neighboring trimers on the nanoparticle surface, consistent with the BLI data (Fig. 5D). A similar effect was also observed for clade A/E 93JP_NH1 UFO-BG-FR, which bound to PGT121 only weakly by BLI but induced a strong Ca^2+^ flux signal in PGT121-expressing B cells, suggesting that cross-linking of B cell receptors (BCRs) by nanoparticles may help overcome the inherent low affinity of trimers. As a result, these nanoparticles will likely elicit a more effective NAb response than trimers, thus providing more promising vaccine immunogens.

### Rapid induction of tier 2 neutralizing antibodies in mice and rabbits

Here, we performed animal immunization to assess gp41_ECTO_-stabilized trimers and nanoparticles derived from an HR1-redesigned BG505 gp140 construct, termed gp140.664.R1, which has been characterized previously (*36*) and in this study. This construct is parental to BG505 UFO, which adds a cleavage site linker, and all UFO-BGs, which replace WT gp41_ECTO_ with BG505 gp41_ECTO_ of the UFO design, because they all contain BG505 gp41_ECTO_ with a redesigned HR1 bend. The high purity, yield, and stability as well as native-like structure and antigenicity of UFO-BG trimers, in large part, originate from this construct. Thus, the in vivo properties of this gp140 construct and its nanoparticles are likely common to most UFO-BGs and their nanoparticles. To obtain an initial readout of immunogenicity, we first assessed these Env antigens in WT mice. In a previous study, BG505 SOSIP trimers failed to elicit autologous tier 2 NAb response to BG505.T332N in WT mice after 18 weeks (*38*). It was concluded that the glycan shield of native-like trimers is impenetrable to murine antibodies due to their short heavy-chain complementarity-determining region 3 (HCDR3) loops. Here, we immunized WT BALB/c mice three times with 3-week intervals to test eight trimers and four nanoparticles (Fig. 6B and fig. S7B, top panel). PIKA, a Toll-like receptor 3 (TLR3) agonist with enhanced T cell and antibody responses reported for a phase 1 rabies vaccine trial (*93*), was used as an adjuvant. IgG was purified from immunized mouse serum to eliminate nonspecific antiviral activity present in the mouse sera (*38*). We assessed the group-combined IgGs with trimer and epitope probes by enzyme-linked immunosorbent assay (ELISA) (fig. S7B). Mouse IgGs elicited by trimers produced in 293 F and ExpiCHO cells (groups 1 and 2) bound differentially to the 293 F–expressed probes, supporting our findings in glycan and B cell repertoire analyses (Fig. 1, D and E). The three scaffolded gp140.681.R1 trimers (groups 3 to 5) elicited stronger antibody responses than the parent gp140.664.R1 trimer (group 2), consistent with our previous study (*56*). While the ferritin nanoparticle (group 6) showed a high level of N332-specific IgG response, all three I3-01 nanoparticles (groups 10 to 12) outperformed the respective trimers containing the same T cell epitopes (groups 7 to 9). We then evaluated the neutralization of group-combined IgGs (3 to 8 mg/ml) in TZM-bl assays (fig. S7C). Autologous tier 2 neutralization was observed for a scaffolded gp140.681.R1 trimer (group 3), a ferritin nanoparticle (group 6), and two I3-01 60-mers (groups 10 and 11), but not for soluble trimers. When the starting IgG concentration was reduced to 1 mg/ml in neutralization assays, group 3 showed a borderline response, whereas one mouse in group 6 and two mice in group 10 generated autologous tier 2 NAbs against BG505.T332N at week 8 (Fig. 6B). The mouse immunization data suggested a potential correlation between antigen valency (the number of trimers presented on an antigen) and rapid elicitation of tier 2 NAbs.

Subsequent analysis was performed on the BG505 gp140.664.R1 trimer (*36*) and its ferritin nanoparticle (*46*) in rabbits with five intramuscular injections over 28 weeks (Fig. 6C). Rabbit plasma collected at six time points during immunization were analyzed by ELISA with a trimer probe and an N332-specific probe (Fig. 6C and fig. S7D). Overall, rabbits in the trimer group yielded comparable median effective concentration (EC_50_) titers that increased steadily over time, whereas rabbits in the ferritin group exhibited a stronger and more rapid antibody response with varying EC_50_ titers. In the ferritin group, rabbit 65 showed a midpoint titer of a trimer-specific response that was 7- to 12-fold greater than that of others at week 6, and rabbit 63 reached a high level of antibody response at week 22. A similar trend was observed in ELISA binding of the N332 probe: The ferritin group showed mean midpoint 3.6- and 2.1-fold greater than the trimer group at weeks 6 and 22, respectively, with a statistically significant *P* value (0.0471) found for week 6. The elevated binding antibody titers might be linked to greater neutralizing activity. To examine this possibility, we conducted TZM-bl neutralization assays longitudinally (Fig. 6C and fig. S7E). The ferritin nanoparticle induced autologous tier 2 NAbs to BG505.T332N more effectively than the trimer, showing a higher percent neutralization at the first point of the sample dilution series (50-fold). At week 6, all rabbits in the ferritin group exhibited consistent autologous tier 2 neutralization, whereas the trimer group did not induce these detectable tier 2 NAbs until week 14 (fig. S7E). A statistical analysis found that the tier 2 NAb response was significantly different between the ferritin group and the trimer group for week 6 (*P* < 0.0001), week 14 (*P* = 0.0016), week 22 (*P* = 0.0330), and week 30 (*P* = 0.0016). Both trimer and ferritin groups showed tier 1 neutralizing activity against clade B SF162 starting at week 6, with greater ID_50_ titers observed for the trimer group, which was consistent with the mouse data (Fig. 6B), although this finding is not statistically significant. Last, the week 22 samples were analyzed against a global panel of 12 viruses using the preimmunization samples as a control (fig. S7, F and G). Neutralization against isolate p398F1 within the same subtype as BG505 (clade A) was observed for both trimer and ferritin groups with ID_50_ titers in the range of 53 to 290, while cross-neutralization was detected for other subtypes at low titers, as indicated by the percent neutralization 2-fold greater than the control at the 20- and 60-fold dilutions.

Our results demonstrate the superior immunogenicity of nanoparticles displaying gp41_ECTO_-stabilized trimers in small animals when compared to trimers alone and confirm the difficulty of eliciting a rapid tier 2 NAb response with soluble trimers independent of the design platform (*26*, *39*–*41*). The immunogenicity of gp140 nanoparticles can be improved by optimizing the vaccine regimen (*42*) or using larger 60-mers designed here and in our previous study (*46*).

## DISCUSSION

Antibody-based HIV-1 vaccines aim to elicit antibodies capable of neutralizing tier 2 strains, thus preventing the acquisition of infection (*2*). The advent of the BG505 SOSIP.644 gp140 trimer and structural analysis of trimers based on SOSIP and other designs have established native-like trimers as a promising vaccine platform (*44*, *45*). These native-like trimers have elicited autologous tier 2 NAb response in rabbits and NHPs following 6 to 12 months of immunization (*40*–*42*, *87*). The failure for SOSIP trimers to elicit tier 2 NAbs in WT mice has been attributed to the dense glycan shield and short HCDR3 loops of murine antibodies (*38*). Design of virus-like particles presenting native-like Env spikes (*94*) and induction of high-quality T follicular helper cells (*89*) were also deemed critical for developing effective HIV-1 vaccine candidates.

Here, we addressed these critical issues in HIV-1 vaccine development with a strategy that focused primarily on Env metastability. Previously, we identified the HR1 bend in gp41_ECTO_ as the primary cause of Env metastability and developed the UFO platform—containing a redesigned HR1 bend and a cleavage site linker—for trimer stabilization (*36*). In this study, we demonstrated that gp41_ECTO_ is the main source of metastability by replacing gp41_ECTO_ of diverse Envs with BG505 gp41_ECTO_ of the UFO design and revealed the evolutionary root of metastability by analyzing UFO trimers of three T/F strains and with a consensus gp41_ECTO_. The importance of gp41_ECTO_ stability for nanoparticle vaccines was illustrated by designing ferritin nanoparticles for diverse subtypes and incorporating T cell help into a large 60-mer, which was only possible when gp41_ECTO_ has minimized metastability. A subset of gp41_ECTO_-stabilized trimers and gp140 nanoparticles derived from the HR1-redesigned BG505 gp140 construct, gp140.664.R1 (*36*), was assessed in WT mice and rabbits, revealing a critical factor in tier 2 NAb elicitation that has not been apparent or clear in current trimer vaccines: Nanoparticles can induce tier 2 NAbs more effectively than individual trimers, indicating a correlation between antigen valency and tier 2 NAb elicitation. High-quality UFO trimers and nanoparticles can be produced in ExpiCHO cells with substantial yield and purity, bearing important implications for vaccine manufacture.

Future investigation should be directed toward optimization and evaluation of the large, 60-meric I3-01 and E2p nanoparticles, which would be expected to be more effective than the ferritin nanoparticle in the elicitation of tier 2 NAbs. Nanoparticles presenting gp41_ECTO_-stabilized trimers of diverse subtypes, when used in a cocktail or sequentially, may be more capable of generating rapid NAb response to each subtype and thus conferring broad protection against HIV-1.

## MATERIALS AND METHODS

### Antibodies

We used a panel of bNAbs and non-NAbs to characterize the antigenicity of various native-like trimers and gp140 nanoparticles. Antibodies were requested from the National Institutes of Health (NIH) AIDS Reagent Program (www.aidsreagent.org/) except for bNAbs PGDM1400, PGT145, PGT121, and PGT151 and non-NAb 19b, which were provided by D.S. and D.R.B. Soluble CD4 in an Ig form (CD4-Ig, catalog no. 12960) was also obtained from the NIH AIDS Reagent Program.

### Expression and purification of HIV-1 Env trimers and nanoparticles

Trimers were transiently expressed in HEK293 F or ExpiCHO cells (Thermo Fisher Scientific) except for crystallographic analysis in which HEK293 F cells were treated with kifunensine. The protocol used for trimer production in HEK293 F cells has been described previously (*36*, *56*). For cleaved HR1-redesigned trimers, the furin plasmid was added during transfection. The protocol for trimer and nanoparticle production in ExpiCHO cells is as follows. Briefly, ExpiCHO cells were thawed and incubated with ExpiCHO Expression Medium (Thermo Fisher Scientific) in a shaker incubator at 37°C, with 135 rpm and 8% CO_2_. When the cells reached a density of 10 × 10^6^ ml^−1^, ExpiCHO Expression Medium was added to reduce cell density to 6 × 10^6^ ml^−1^ for transfection. The ExpiFectamine CHO/plasmid DNA complexes were prepared for 100-ml transfection in ExpiCHO cells following the manufacturer’s instructions. For SOSIP and HR1-redesigned trimers as well as the I3-01 nanoparticles presenting an HR1-redesigned trimer of the BG505 strain, 80 μg of antigen plasmid, 30 μg of furin plasmid, and 320 μl of ExpiFectamine CHO reagent were mixed in 7.7 ml of cold OptiPRO medium (Thermo Fisher Scientific), whereas for UFO trimers (including UFO-BG and UFO-C) as well as UFO-BG-FR nanoparticles, 100 μg of antigen plasmid was used without furin. After the first feed on day 1, ExpiCHO cells were cultured in a shaker incubator at 32°C, with 120 rpm and 8% CO_2_ following the Max Titer protocol with an additional feed on day 5 (Thermo Fisher Scientific). Culture supernatants were harvested 13 to 14 days after transfection, clarified by centrifugation at 4000 rpm for 20 min, and filtered using a 0.45-μm filter (Thermo Fisher Scientific). For trimers, Env protein was extracted from the culture supernatants using a GNL column (Vector Labs), whereas for nanoparticles, Env fusion protein was purified using a 2G12 affinity column. Some trimers were further purified by SEC on a Superdex 200 Increase 10/300 GL column or a HiLoad 16/600 Superdex 200 PG column (GE Healthcare). The purity of I3-01 nanoparticles was characterized by SEC on a Superose 6 10/300 GL column. For both trimers and nanoparticles, protein concentration was determined using UV_280_ absorbance with theoretical extinction coefficients.

### Analysis of total and site-specific glycosylation profiles

The total glycan profiles of ExpiCHO and 293 F–produced trimers were generated by HILIC-UPLC. N-linked glycans were enzymatically released from Envs via in-gel digestion with peptide N-glycosidase F (PNGase F), subsequently fluorescently labeled with 2-aminobenzoic acid, and analyzed by HILIC-UPLC, as previously described (*53*, *54*, *95*, *96*). Digestion of released glycans with Endo H enabled the quantitation of oligomannose-type glycans (*95*). The composition of the glycans was determined by analyzing released glycans from trimers by PNGase F digestion using ion mobility MS (*53*). Negative ion mass, collision-induced dissociation, and ion mobility spectra were recorded with a Waters SYNAPT G2-Si mass spectrometer (Waters Corp.) fitted with a nanoelectrospray ion source. Waters DriftScope (version 2.8) software and MassLynx (version 4.1) were used for data acquisition and processing. Spectra were interpreted as described previously (*97*–*100*). The results obtained served as the basis for the creation of sample-specific glycan libraries, which were used for subsequent site-specific N-glycosylation analyses. For site-specific N-glycosylation analysis, before digestion, trimers were denatured and alkylated by incubation for 1 hour at room temperature in a 50 mM tris-HCl (pH 8.0) buffer containing 6 M urea and 5 mM dithiothreitol (DTT), followed by addition of 20 mM iodoacetamide (IAA) for a further 1 hour at room temperature in the dark and then additional DTT (20 mM) for another 1 hour to eliminate any residual IAA. The alkylated trimers were buffer-exchanged into 50 mM tris-HCl (pH 8.0) using Vivaspin columns and digested separately with trypsin and chymotrypsin (Mass Spectrometry Grade, Promega) at a ratio of 1:30 (w/w). Glycopeptides were selected from the protease-digested samples using the ProteoExtract Glycopeptide Enrichment Kit (Merck Millipore). Enriched glycopeptides were analyzed by LC–electrospray ionization MS on an Orbitrap fusion mass spectrometer (Thermo Fisher Scientific), as previously described (*53*), using higher-energy collisional dissociation fragmentation. Data analysis and glycopeptide identification were performed using Byonic (version 2.7) and Byologic software (version 2.3; Protein Metrics Inc.), as previously described (*53*).

### Blue native polyacrylamide gel electrophoresis

Env proteins and nanoparticles were analyzed by BN-PAGE and stained with Coomassie blue. The protein samples were mixed with G250 loading dye and added to a 4 to 12% bis-tris NuPAGE gel (Life Technologies). BN-PAGE gels were run for 2.5 hours at 150 V using the NativePAGE running buffer (Life Technologies) according to the manufacturer’s instructions.

### Differential scanning calorimetry

Thermal stability of UFO, UFO-BG, and UFO-C trimers expressed in ExpiCHO and 293 F cells and trimer-presenting nanoparticles was measured using a MicroCal VP-Capillary calorimeter (Malvern) in phosphate-buffered saline (PBS) buffer at a scanning rate of 90°C hour^−1^ from 20° to 120°C. Data were analyzed using the VP-Capillary DSC automated data analysis software.

### Protein production and purification for crystallization

The clade B tier 3 H078.14 UFO-BG trimer was expressed in FreeStyle 293 F cells treated with kifunensine and purified from culture supernatant using a 2G12 affinity column followed by SEC. Fabs PGT124 and 35O22 were transiently transfected into FreeStyle 293F cells (Invitrogen) and purified using a LC-λ capture select column, before further purification by ion exchange chromatography and SEC on a Superdex 200 16/600 column. The trimer complexes were prepared by mixing H078.14 UFO-BG trimer protein with PGT124 and 35O22 at a molar ratio of 1:3.5 for 30 min at room temperature. To decrease glycan heterogeneity, deglycosylation was conducted on the PGT124- and 35O22-bound H078.14 UFO-BG Env protein produced in 293 F cells with Endo H (New England Biolabs) overnight at 4°C. The trimer complexes were subjected to crystal trials after further purification by SEC.

### Protein crystallization and data collection

The SEC-purified H078.14 UFO-BG trimer complexes were concentrated to ~8 mg/ml before being subjected to extensive crystallization trials at both 4° and 20°C using our automated CrystalMation robotic system (Rigaku) at The Scripps Research Institute (TSRI) (*101*). Crystals for protein complex containing Fab PGT124 and 35O22 bound to the UFO-BG trimer were obtained from 0.1 M tris (pH 7.4), 0.2 M lithium sulfate, and 6% (w/v) polyethylene glycol 4000, using the sitting drop (0.2 μl) vapor diffusion method, and harvested and cryoprotected with 30% ethylene glycol, followed by immediate flash cooling in liquid nitrogen. The best crystal diffracted to a resolution of 4.60 Å, and diffraction data were collected at the Advanced Photon Source (APS) beamline 23IDB, processed with HKL-2000 (*102*), and indexed in space group *P*6_3_ with 98% completeness with the following unit cell parameters: *a* = *b* = 127.3 Å and *c* = 316.3 Å (table S1).

### Structure determination and refinement

The H078.14 UFO-BG trimer structure bound to PGT124 and 35O22 was solved by molecular replacement (MR) using Phaser (*103*) with the BG505 SOSIP.664 protomer:35O22 component from a previous structure (PDB: 5CEZ) (*22*), followed by the PGT124 Fab structure (PDB: 4R26) (*23*) as the MR models. The structures were refined using Phenix (*104*), with Coot used for model building (*105*) and MolProbity for structure validation (*106*). Because of the limited resolution of the datasets, two *B*-factor groups per residue were used in refinement. The final *R*_cryst_ and *R*_free_ values for complex structure are 31.2 and 34.0%. Figures were generated with PyMOL and Chimera (*107*). In the crystal structure, residues were numbered according to the Kabat definition (*108*) for the Fabs and according to the HXBc2 system for gp140.

### Negative-stain EM

UFO-BG trimers, UFO-C trimers, and gp140 trimer-presenting nanoparticles were analyzed by negative-stain EM. A 3-μl aliquot containing trimers or nanoparticles (~0.01 mg/ml) was applied for 15 s onto a carbon-coated 400 Cu mesh grid that had been glow discharged at 20 mA for 30 s and then negatively stained with 2% (w/v) uranyl formate for 30 s. Data were collected using a FEI Tecnai Spirit electron microscope operating at 120 kV, with an electron dose of ~25 e^−^ Å^−2^ and a magnification of ×52,000 that resulted in a pixel size of 2.05 Å at the specimen plane. Images were acquired with a Tietz 4k × 4k TemCam-F416 complementary metal-oxide semiconductor (CMOS) camera using a nominal defocus of 1500 nm and the Leginon package (*109*). UFO-BG trimer particles were selected automatically from the raw micrographs using DoG Picker (*110*), while trimer-presenting nanoparticles were selected manually using the Appion Manual Picker (*111*). Both were put into particle stack using the Appion software package (*111*). Reference-free 2D class averages were calculated using particles binned by two via iterative multivariate statistical analysis/multireference alignment and sorted into classes (*112*). To analyze the quality of the trimers (native-like and non-native), the reference-free 2D class averages were examined by eye, as previously described (*26*).

### Biolayer interferometry

The kinetics of trimer and nanoparticle binding to bNAbs, non-NAbs, and CD4-Ig was measured using an Octet Red96 instrument (fortéBio, Pall Life Sciences). All assays were performed with agitation set to 1000 rpm in fortéBio 1× kinetic buffer. The final volume for all the solutions was 200 μl per well. Assays were performed at 30°C in solid black 96-well plates (Geiger Bio-One). Antibody (5 μg ml^−1^) in 1× kinetic buffer was loaded onto the surface of anti-human Fc capture (AHC) biosensors for 300 s. A 60-s biosensor baseline step was applied before the analysis of the association of the antibody on the biosensor to the antigen in solution for 200 s. A twofold concentration gradient of antigen, starting at 200 nM for trimers and 14 to 35 nM for nanoparticles depending on the size, was used in a titration series of six. The dissociation of the interaction was followed for 300 s. Correction of baseline drift was performed by subtracting the mean value of shifts recorded for a sensor loaded with antibody but not incubated with antigen and for a sensor without antibody but incubated with antigen. Octet data were processed by fortéBio’s data acquisition software version 8.1. Notably, for apex-directed bNAbs, experimental data were fitted with the binding equations describing a 2:1 interaction to achieve the optimal fitting results. CD4-Ig binding experiments were performed following the same procedure described above. Paired *t* test in Prism (two groups, *n* = 10) was used to determine whether trimer-antibody binding signals were statistically different (*P* < 0.05) upon gp41_ECTO_ swapping.

### B cell activation assay

Generation of K46 B cell lines expressing PGT121, PGT145, or VRCO1 has been previously described (*92*). Briefly, K46 cells expressing a doxycycline-inducible form of bNAb BCRs were maintained in advanced Dulbecco’s modified Eagle’s medium (DMEM) (Gibco), supplemented with 10% fetal calf serum, penicillin/streptomycin antibiotics, and puromycin (2 μg/ml; Gibco). Cells were treated overnight in doxycycline (1 μg/ml; Clontech) to induce human BCR expression. After loading with Indo-1 (Molecular Probes) at 1 μM for 1 hour at 37°C, washed cells were stimulated with the indicated agents at a concentration of 10 μg ml^−1^: anti-mouse IgM (Jackson ImmunoResearch), UFO-BG or an HR1-redesigned gp140 trimer (*36*) with a T helper epitope (PADRE) fused to the C terminus, and UFO-BG-FR or I3-01 nanoparticle presenting an HR1-redesigned gp140 trimer. Calcium mobilization was assessed on an LSR II flow cytometer (BD Biosciences). In each run, the unstimulated B cells were first recorded for 60 s; immunogen was added, mixed thoroughly, and recorded for 180 s; and then 1 μl of ionomycin (1 μg ml^−1^; Sigma) was added and recorded for another 60 s to verify Indo loading.

### Immunization and serum IgG purification

The Institutional Animal Care and Use Committee (IACUC) guidelines were followed with animal subjects tested in the immunization study. Seven-week-old BALB/c mice were purchased from the Jackson Laboratory. The mice were housed in ventilated cages in environmentally controlled rooms at TSRI, in compliance with an approved IACUC protocol and AAALAC (Association for Assessment and Accreditation of Laboratory Animal Care) International guidelines. At week 0, each mouse was immunized with 200 μl of antigen/adjuvant mix containing 50 μg of antigen and 100 μl of the AddaVax adjuvant (InvivoGen) or 50 μl of the PIKA adjuvant (Yisheng Biopharma) as per the manufacturer’s instruction via the intraperitoneal route. At weeks 3 and 6, the animals were boosted with 50 μg of antigen formulated in the AddaVax or PIKA adjuvant. At week 8, the animals were terminally bled through the retro-orbital membrane using heparinized capillary tubes. Samples were diluted with an equal volume of PBS and then overlaid on 4.5 ml of Ficoll/Histopaque in a 15-ml SepMate tube (STEMCELL Technologies) and spun at 1200 rpm for 10 min at 20°C to separate plasma and cells. The plasma was heat inactivated at 56°C for 1 hour, spun at 1200 rpm for 10 min, and sterile filtered. The cells were washed once in PBS and then resuspended in 1 ml of ACK red blood cell (RBC) lysis buffer (Lonza). After two rounds of washing with PBS, peripheral blood mononuclear cells (PBMCs) were resuspended in 2 ml of BAMBANKER Freezing Media (Lymphotec Inc.). Spleens were also harvested and grounded against a 40-μm cell strainer (BD Falcon) to release splenocytes into a cell suspension. The cells were centrifuged, washed in PBS, treated with 10 ml of RBC lysis buffer as per the manufacturer’s specifications, and resuspended in BAMBANKER Freezing Media for cell freezing. One-third of the total serum per mouse, or 600 μl of serum, was purified using a 0.2-ml protein G spin kit (Thermo Fisher Scientific) following the manufacturer’s instructions. Purified IgGs from four mice in each group were combined for characterization by ELISA binding and initial neutralization assays, while purified IgGs from individual mice in two nanoparticle groups, 6 and 10, were used for further analysis of HIV-1 neutralization in TZM-bl assays. Rabbit immunization and blood sampling were carried out under a subcontract at Covance (Denver, PA). Two groups of female New Zealand White rabbits, four rabbits per group, were immunized intramuscularly with 30 μg of the trimer or nanoparticle formulated in 250 μl of adjuvant AddaVax (InvivoGen) with a total volume of 500 μl, at weeks 0, 4, 12, 20, and 28. Blood samples, 15 ml each time, were collected at day −10 and weeks 1, 6, 14, 22, and 28, as shown in Fig. 6C. Plasma was separated from blood and heat inactivated for ELISA binding and neutralization assays.

### Enzyme-linked immunosorbent assay

Each well of a Costar 96-well assay plate (Corning) was first coated with 50 μl of PBS containing 0.2 μg of the appropriate antigens. The plates were incubated overnight at 4°C and then washed five times with wash buffer containing PBS and 0.05% (v/v) Tween 20. Each well was then coated with 150 μl of a blocking buffer consisting of PBS, blotting-grade blocker (20 mg ml^−1^; Bio-Rad), and 5% (v/v) fetal bovine serum. The plates were incubated with the blocking buffer for 1 hour at room temperature and then washed five times with wash buffer. In the mouse sample analysis, purified IgGs were diluted in the blocking buffer to a maximum concentration of 100 μg ml^−1^, whereas in the rabbit sample analysis, heat-inactivated plasma was diluted by 50-fold in the blocking buffer; both samples were subjected to a 10-fold dilution series. For each sample dilution, a total volume of 50 μl was added to the wells. Each plate was incubated for 1 hour at room temperature and then washed five times with wash buffer. A 1:2000 dilution of horseradish peroxidase–labeled goat anti-mouse or anti-rabbit IgG antibody (Jackson ImmunoResearch Laboratories) was then made in the wash buffer, with 50 μl of this diluted secondary antibody added to each well. The plates were incubated with the secondary antibody for 1 hour at room temperature and then washed five times with wash buffer. Last, the wells were developed with 50 μl of 3,3′, 5,5;-tetramethylbenzidine (TMB) (Life Technologies) for 3 to 5 min before stopping the reaction with 50 μl of 2 N sulfuric acid. The resulting plate readouts were measured at a wavelength of 450 nm.

### Pseudovirus production and neutralization assays

Pseudoviruses were generated by transfection of 293 T cells with an HIV-1 Env-expressing plasmid and an Env-deficient genomic backbone plasmid (pSG3ΔEnv), as described previously (*113*). HIV-1 Env-expressing vectors for BG505 (catalog no. 11518), SF162 (catalog no. 10463), and the global panel (catalog no. 12670) were obtained through the NIH AIDS Reagent Program, Division of AIDS, National Institute of Allergy and Infectious Diseases, NIH (www.aidsreagent.org/). A T332N mutation was introduced into BG505 Env to produce the BG505.T332N clone. Pseudoviruses were harvested 72 hours after transfection for use in neutralization assays. Neutralizing activity of purified mouse serum IgGs or heat-inactivated rabbit plasma was assessed using a single round of replication pseudovirus assay and TZM-bl target cells, as described previously (*113*). Briefly, pseudovirus was incubated with serial dilutions of mouse serum IgG or rabbit plasma in a 96-well flat-bottom plate for 1 hour at 37°C before TZM-bl cells were seeded in the plate. In the analysis of rabbit samples against autologous clade A tier 2 BG505.T332N and clade B tier 1 SF162, the plasma was diluted by 50-fold and subjected to a 3-fold dilution series in the TZM-bl assays. Luciferase reporter gene expression was quantified 48 to 72 hours after infection upon lysis and addition of Bright-Glo Luciferase substrate (Promega). Data were retrieved from a BioTek microplate reader with Gen 5 software, the average background luminescence from a series of uninfected wells was subtracted from each experimental well, and neutralization curves were generated using GraphPad Prism 6.0, in which values from experimental wells were compared against a well containing virus only. To determine ID_50_ values, dose-response curves were fit by nonlinear regression in Prism. Unpaired *t* test (nonparametric) in Prism was used to determine whether the two rabbit groups immunized with trimer and ferritin nanoparticle (two groups, *n* = 14 when antibody titer was used or *n* = 8 when percent neutralization with duplicates was used) were significantly different (*P* < 0.05) in plasma binding and neutralization. Rabbit plasma neutralization against a global panel of 12 isolates was briefly assessed for two time points, before immunization (day −10) and week 22, without duplicates. To increase the sensitivity of detection, rabbit plasma was diluted by 20-fold followed by a 3-fold dilution series in TZM-bl assays. To reduce false positives caused by nonspecific antiviral proteins present in the rabbit plasma, a simple metric that directly compares the percent neutralization of the week 22 sample to that of the day −10 sample from the same animal subject at the 20- and 60-fold dilutions was used. The week 22 signals at both dilutions had to be twofold greater than the corresponding day −10 signals for a sample to be considered neutralizing a virus clone in the global panel.

### Mouse repertoire sequencing and bioinformatics analysis

A 5′-RACE [rapid amplification of complementary DNA (cDNA) ends] protocol has been developed for unbiased sequencing of mouse B cell repertoires, as previously described (*56*). Briefly, RNA (including mRNA) was extracted from total PBMCs of each mouse into 30 μl of water with the RNeasy Mini Kit (Qiagen). 5′-RACE was performed with the SMARTer RACE cDNA Amplification Kit (Clontech). The Ig polymerase chain reactions (PCRs) were set up with Platinum Taq High-Fidelity DNA Polymerase (Life Technologies) in a total volume of 50 μl, with 5 μl of cDNA as template, 1 μl of 5′-RACE primer, and 1 μl of 10 μM reverse primer. The 5′-RACE primer contained a PGM/S5 P1 adaptor, while the reverse primer contained a PGM/S5 A adaptor. We adapted the mouse 3′-C_γ_1-3 and 3′-C_μ_ inner primers as reverse primers for 5′-RACE PCR processing of the heavy chains. A total of 25 cycles of PCR was performed, and the expected PCR products (500 to 600 base pairs) were gel purified (Qiagen). NGS was performed on the Ion S5 system. Briefly, heavy-chain libraries from the same group were quantitated using Qubit 2.0 Fluorometer with the Qubit dsDNA HS Assay Kit and then mixed using a ratio of 1:1:1:1 for sequencing. Template preparation and (Ion 520) chip loading were performed on Ion Chef using the Ion 520/530 Ext Kit, followed by sequencing on the Ion S5 system with default settings. The mouse antibodyomics pipeline was used to process the raw data and to determine the distributions of heavy-chain germline gene usage.

## Supplementary Material

http://advances.sciencemag.org/cgi/content/full/4/11/eaau6769/DC1

## SUPPLEMENTARY MATERIALS

Supplementary material for this article is available at http://advances.sciencemag.org/cgi/content/full/4/11/eaau6769/DC1 Fig. S1. Effect of expression system on antigenicity and glycosylation of BG505 Env trimers. Fig. S2. Biochemical and biophysical characterization of diverse Env trimers. Fig. S3. Structural characterization of diverse UFO-BG trimers. Fig. S4. Antigenic profiles of UFO and UFO-BG trimers derived from 10 strains of five subtypes assessed against a panel of 11 bNAbs, 8 non-NAbs, and CD4-Ig. Fig. S5. Evolutionary root of metastability and design of UFO-C trimers containing a database-derived ancestral gp41_ECTO_. Fig. S6. Characterization of gp41_ECTO_-stabilized trimer-presenting nanoparticles. Fig. S7. B cell activation and in vivo evaluation of gp41_ECTO_-stabilized trimers and nanoparticles. Table S1. X-ray data collection and refinement statistics.
