# Supplementary material for: HIV-1 vaccine design through minimizing envelope metastability
Source: Sci Adv. 2018 Nov 21;4(11):eaau6769. doi: 10.1126/sciadv.aau6769 (PMC6248932; doi:10.1126/sciadv.aau6769)
Supplement: http://advances.sciencemag.org/cgi/content/full/4/11/eaau6769/DC1 [file supp_4_11_eaau6769__index.html]

Science Advances | Science Advances

## Supplementary Materials

**This PDF file includes:**

- Fig. S1. Effect of expression system on antigenicity and glycosylation of BG505 Env trimers.
- Fig. S2. Biochemical and biophysical characterization of diverse Env trimers.
- Fig. S3. Structural characterization of diverse UFO-BG trimers.
- Fig. S4. Antigenic profiles of UFO and UFO-BG trimers derived from 10 strains of five subtypes assessed against a panel of 11 bNAbs, 8 non-NAbs, and CD4-Ig.
- Fig. S5. Evolutionary root of metastability and design of UFO-C trimers containing a database-derived ancestral gp41ECTO.
- Fig. S6. Characterization of gp41ECTO-stabilized trimer-presenting nanoparticles.
- Fig. S7. B cell activation and in vivo evaluation of gp41ECTO-stabilized trimers and nanoparticles.
- Table S1. X-ray data collection and refinement statistics.

Download PDF

**Files in this Data Supplement:**

- Adobe PDF - aau6769\_SM.pdf
