# Supplementary material for: HIV-1 vaccine design through minimizing envelope metastability
Source: Sci Adv. 2018 Nov 21;4(11):eaau6769. doi: 10.1126/sciadv.aau6769 (PMC6248932; doi:10.1126/sciadv.aau6769)
Supplement: http://advances.sciencemag.org/cgi/content/full/4/11/eaau6769/DC1 [file aau6769_SM.pdf]

Science Advances
AAAS

advances.sciencemag.org/cgi/content/full/4/11/eaau6769/DC1

Supplementary Materials for

HIV-1 vaccine design through minimizing envelope metastability

Linling He, Sonu Kumar, Joel D. Allen, Deli Huang, Xiaohe Lin, Colin J. Mann, Karen L. Saye-Francisco, Jeffrey Copps, Anita Sarkar, Gabrielle S. Blizard, Gabriel Ozorowski, Devin Sok, Max Crispin, Andrew B. Ward, David Nemazee, Dennis R. Burton, Ian A. Wilson*, Jiang Zhu*

*Corresponding author. Email: wilson@scripps.edu (I.A.W.); jiang@scripps.edu (J.Z.)

Published 21 November 2018, *Sci. Adv.* **4**, eaau6769 (2018)
DOI: 10.1126/sciadv.aau6769

This PDF file includes:

Fig. S1. Effect of expression system on antigenicity and glycosylation of BG505 Env trimers.
Fig. S2. Biochemical and biophysical characterization of diverse Env trimers.
Fig. S3. Structural characterization of diverse UFO-BG trimers.
Fig. S4. Antigenic profiles of UFO and UFO-BG trimers derived from 10 strains of five subtypes assessed against a panel of 11 bNAbs, 8 non-NAbs, and CD4-Ig.
Fig. S5. Evolutionary root of metastability and design of UFO-C trimers containing a database-derived ancestral $gp41_{ECTO}$.
Fig. S6. Characterization of $gp41_{ECTO}$-stabilized trimer-presenting nanoparticles.
Fig. S7. B cell activation and in vivo evaluation of $gp41_{ECTO}$-stabilized trimers and nanoparticles.
Table S1. X-ray data collection and refinement statistics.

A

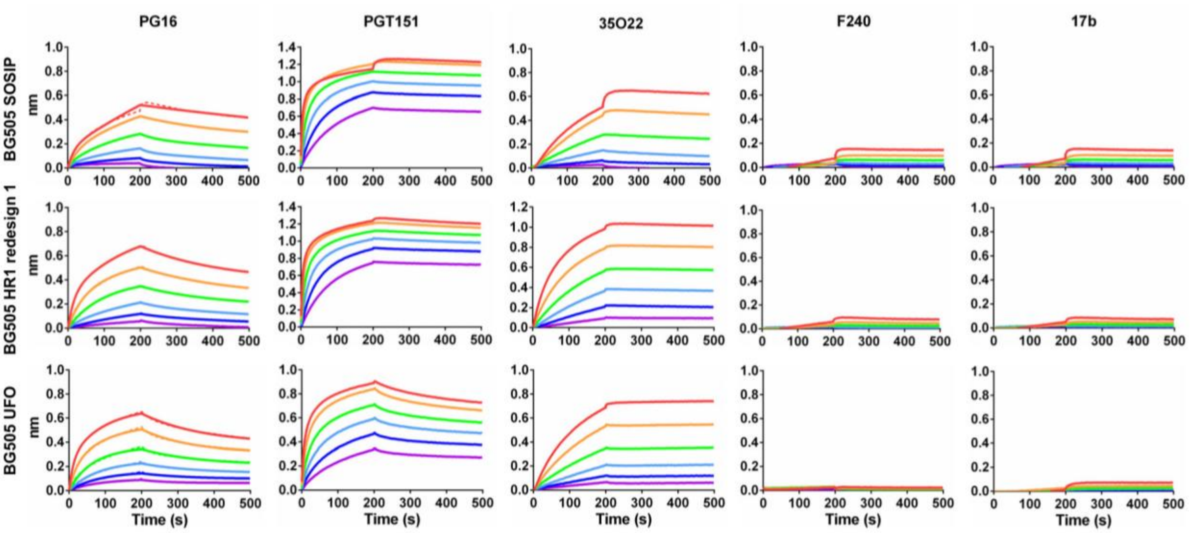

B

HEK293 F

TOTAL OLIGOMANNOSE CONTENT	
**Man5-9**	56.42%
**Man5**	5.99%
**Man6**	3.24%
**Man7**	5.52%
**Man8**	15.48%
**Man9**	26.19%

ExpiCHO

TOTAL OLIGOMANNOSE CONTENT	
**Man5-9**	64.49%
**Man5**	6.17%
**Man6**	5.12%
**Man7**	4.15%
**Man8**	12.81%
**Man9**	29.69%

C

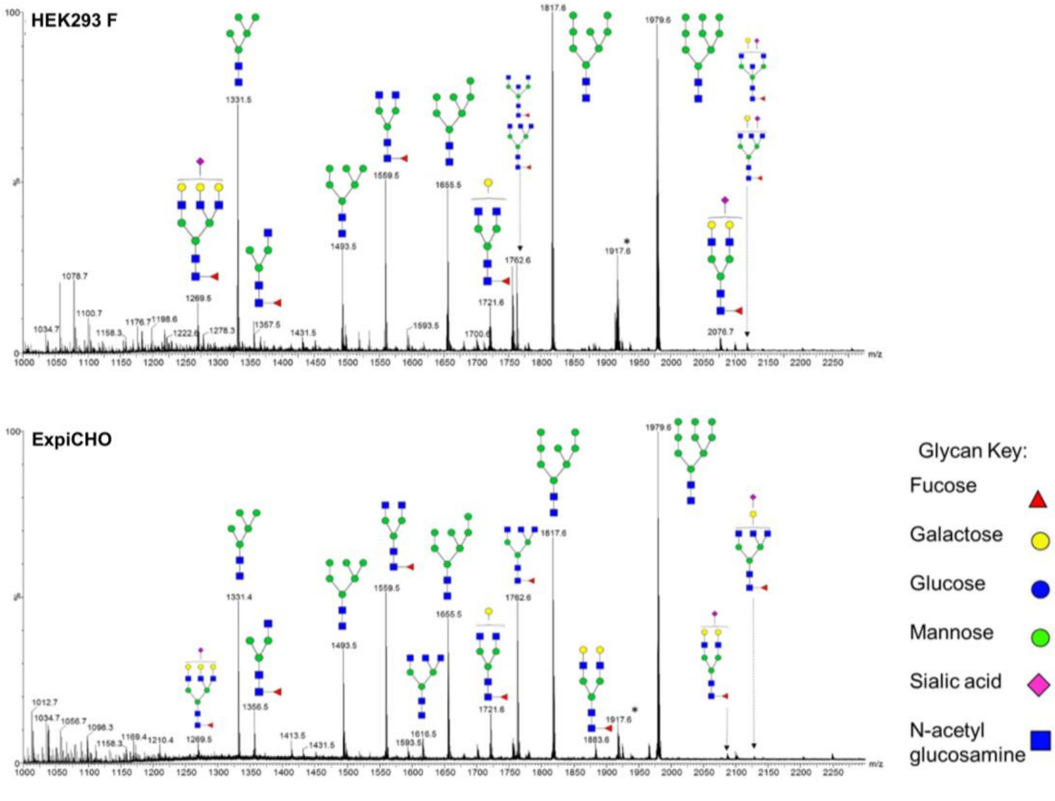

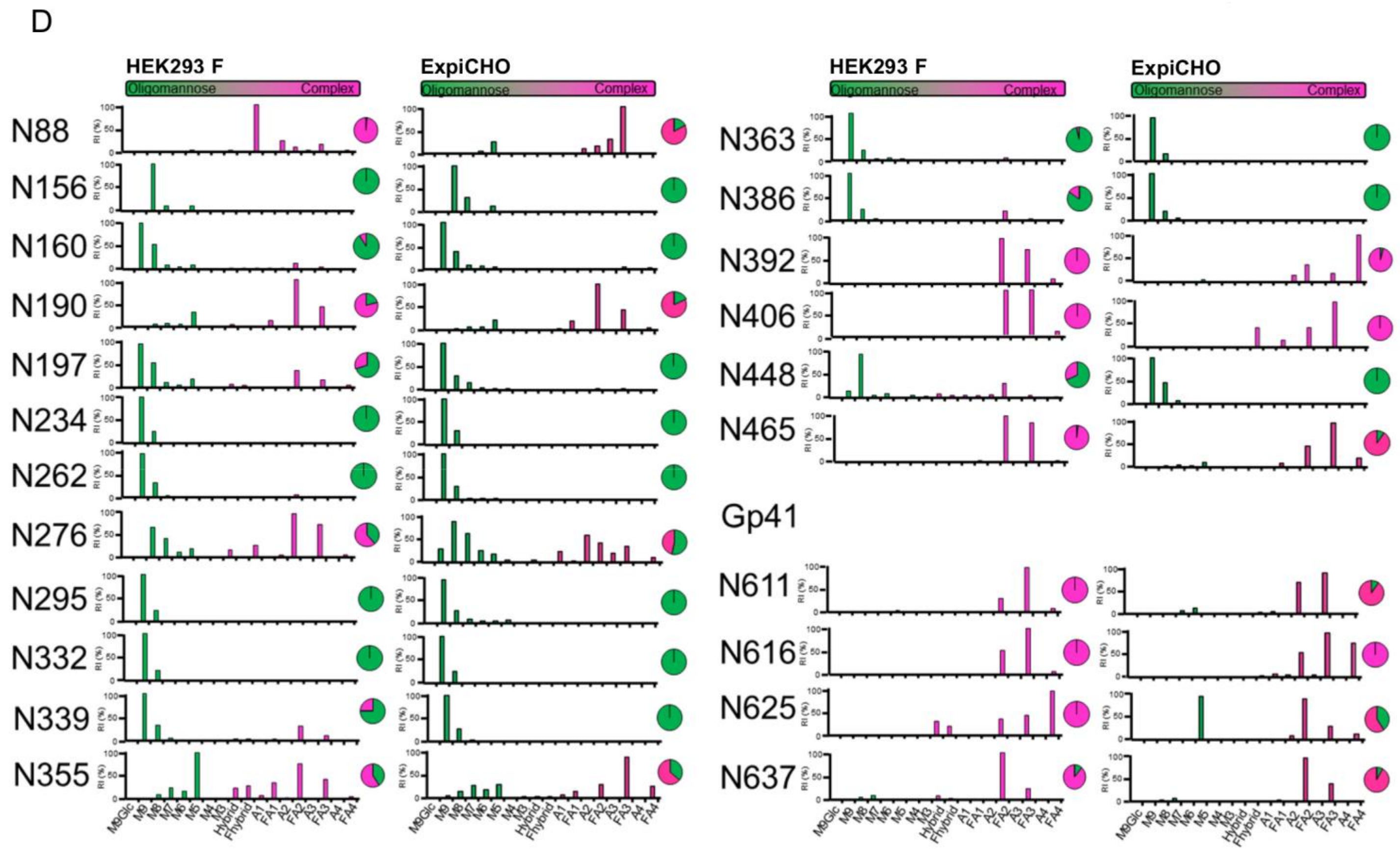

Fig. S1. Effect of expression system on antigenicity and glycosylation of BG505 Env trimers. (**A**) Additional antigenic profiles for SOSIP, HR1-redesigned, and UFO trimers derived from clade A BG505. Sensorgrams were obtained from an Octet RED96 instrument using a titration series of six trimer concentrations (200-6.25 nM by 2-fold dilution). (**B**) Total oligomannose content for a BG505 trimer produced in HEK293 F and ExpiCHO cells. (**C**) Ion mobility mass spectrometry analysis of a BG505 trimer produced in HEK293 F and ExpiCHO cells. Mobility-extracted singly charged negative-ion electrospray spectra are shown for N-linked glycans on gp140. (**D**) Quantitative site-specific N-glycosylation of a BG505 trimer produced in HEK293 F and ExpiCHO cells, with relative quantification shown for N-glycosylation sites on both gp120 and $gp41_{ECTO}$. The gp140 construct used in (B)-(D) is the HR1 redesign 1 (or termed gp140.664.R1), which is the immediate predecessor of the UFO design.

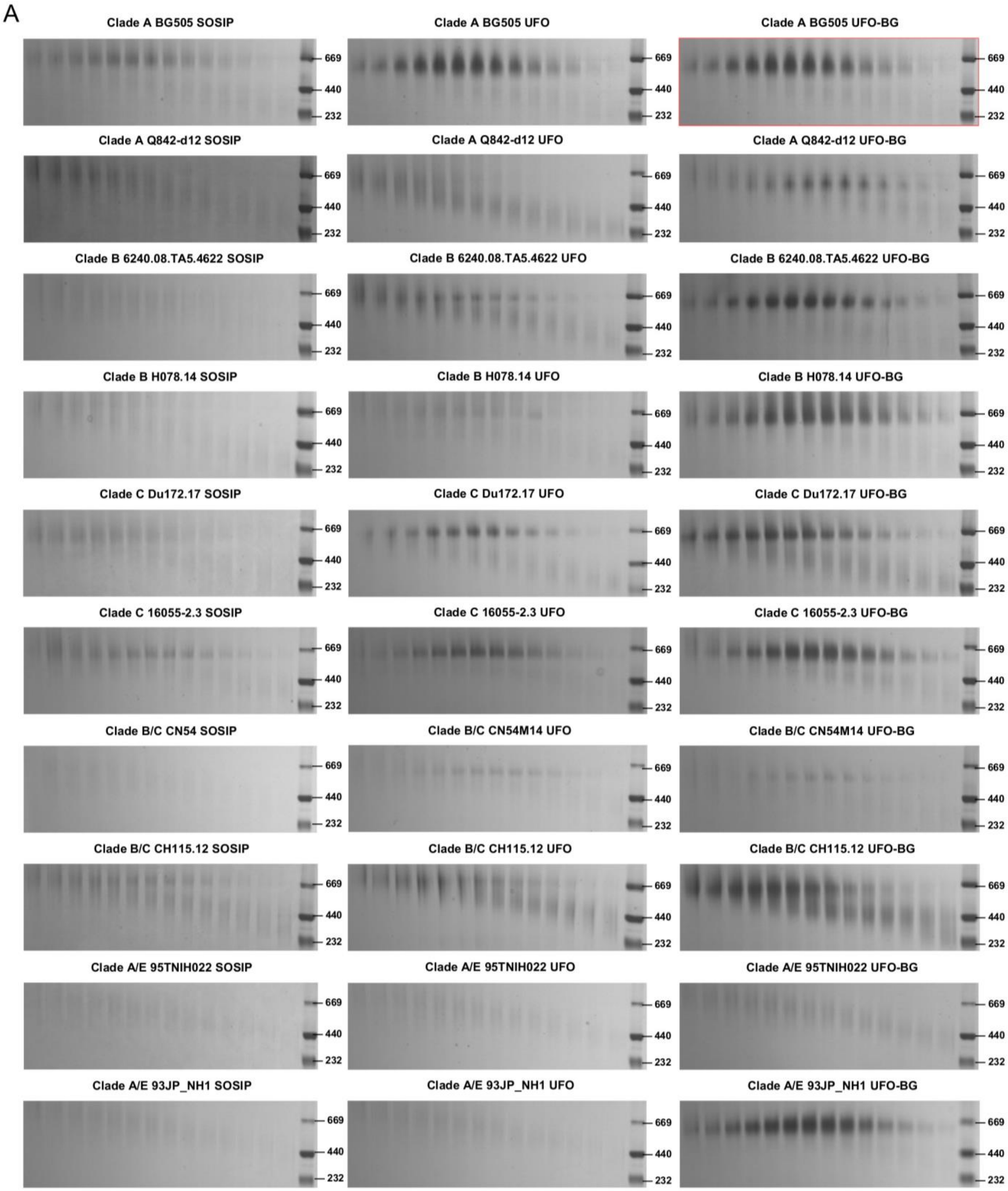
A
Clade A BG505 SOSIP
Clade A BG505 UFO
Clade A BG505 UFO-BG
Clade A Q842-d12 SOSIP
Clade A Q842-d12 UFO
Clade A Q842-d12 UFO-BG
Clade B 6240.08.TA5.4622 SOSIP
Clade B 6240.08.TA5.4622 UFO
Clade B 6240.08.TA5.4622 UFO-BG
Clade B H078.14 SOSIP
Clade B H078.14 UFO
Clade B H078.14 UFO-BG
Clade C Du172.17 SOSIP
Clade C Du172.17 UFO
Clade C Du172.17 UFO-BG
Clade C 16055-2.3 SOSIP
Clade C 16055-2.3 UFO
Clade C 16055-2.3 UFO-BG
Clade B/C CN54 SOSIP
Clade B/C CN54M14 UFO
Clade B/C CN54M14 UFO-BG
Clade B/C CH115.12 SOSIP
Clade B/C CH115.12 UFO
Clade B/C CH115.12 UFO-BG
Clade A/E 95TNIH022 SOSIP
Clade A/E 95TNIH022 UFO
Clade A/E 95TNIH022 UFO-BG
Clade A/E 93JP_NH1 SOSIP
Clade A/E 93JP_NH1 UFO
Clade A/E 93JP_NH1 UFO-BG
669
440
232

B **Thermal stability of ExpiCHO-expressed UFO-BG trimers**

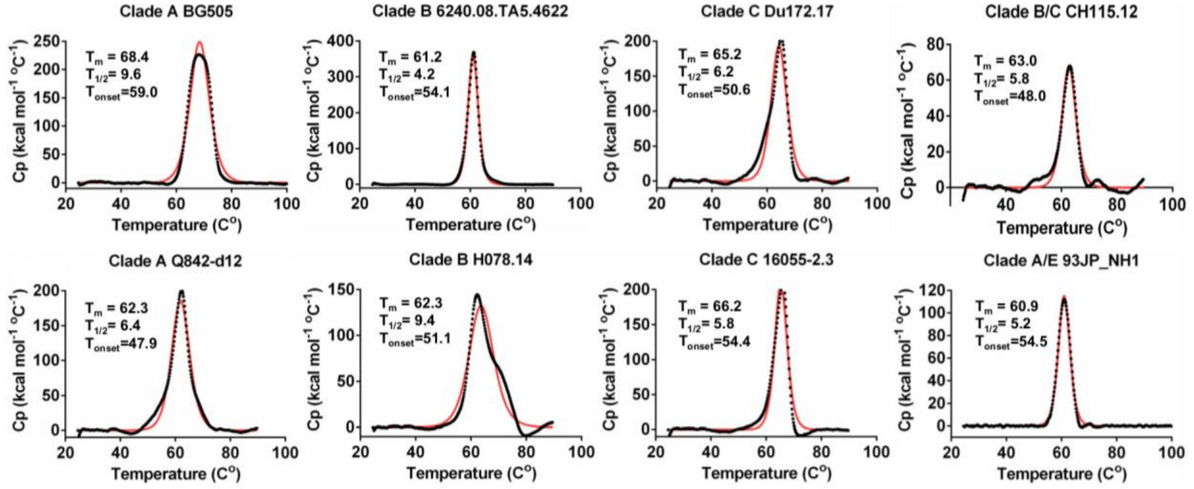

C **Thermal stability of ExpiCHO-expressed UFO trimers**

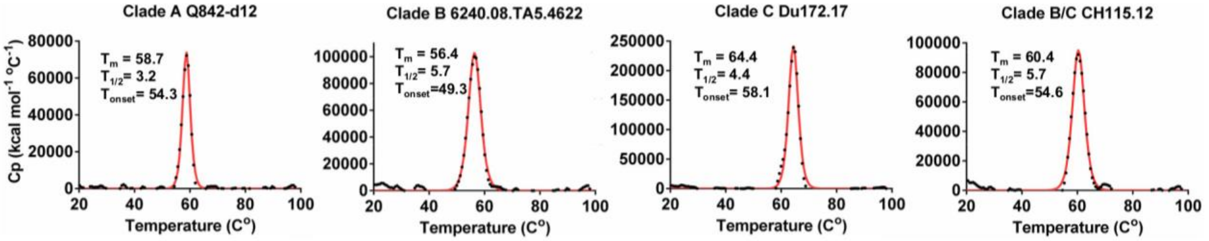

D **Thermal stability of 293 F-expressed UFO/UFO-BG trimers**

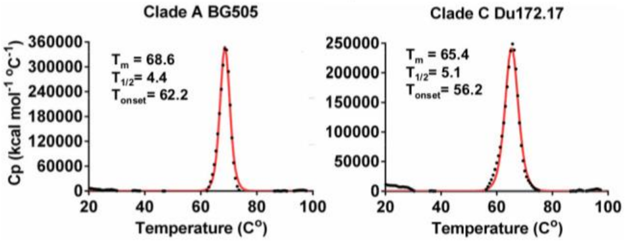

E

>CN54Mut14 (the generic UFO design)

MDAMKRGLCCVLLLCGAVFVSPSQEIHARFRRGARVGNLWVTVYYGVPVWKdAeTTLFCASDAKAYDTEkHNVWATHACVPADPNPQEMVLENVT
ENFNMWKNEMVNQMQtDVISLWDQSLKPCVKLTPLCVTLECRNVSSNSNDTYHETYHESMKEMKNCSFNATTVlRDRKQTVYALFYRLDIVPLTK
KNYSENSSEYYRLINCNTSAITQACPKVTFDPIPIHYCTPAGYAILKCNDKIFNGTGPCHNVSTVQCTHGIKPVVSTQLLLNGSLAEGEIIIRSE
NLTNNVKTIIVHLNQSVEIVCTRPGNNTRKSIRIGPGQTFYATGDIIGDIRQAHCNISEDKWNETLQRVSKKLAEHFQNKTIKFASSSGGDLEVT
THSFNCgGEFFYCNTSGLFNGAYTPNGTKSNSSSIITIPCRIKQIINMWQrVGRAMYAPPIKGNIrCvSNITGLLLVRDGGTEPNDTETFRPGGG
DMRNNWRSELYKYKVVEIKPLGVAPTrCKRRVVEGGGGSGGGGSAVGIGAVFLGFLGVAGSTMGAASmTLTVQARnLLSGGSGSGSGSTVWGIKQ
LQTRVLAIERYLrDQQLLGIWGCSGKLICCTnVPWNSSWSNKSQKEIWDNMTWMQWDKEISNYTNTVYRLLEESQNQQERNEKDLLALD

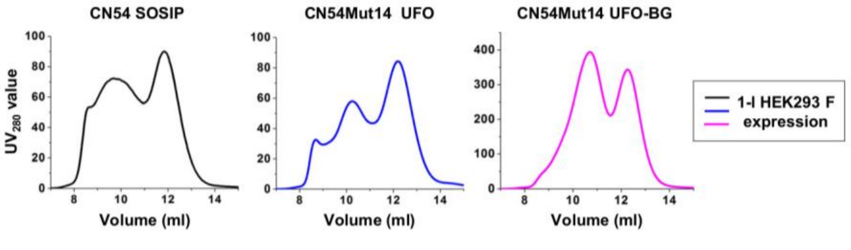

F

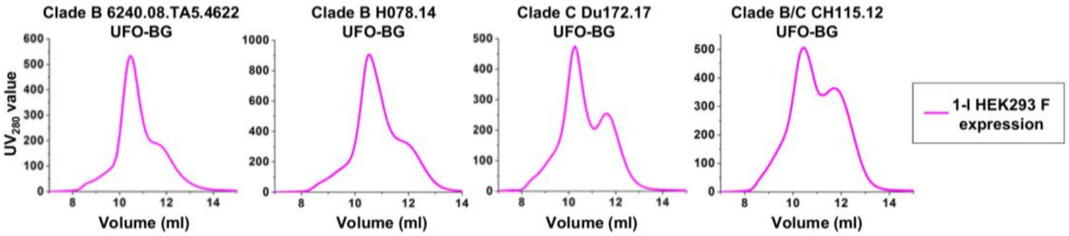

Fig. S2. Biochemical and biophysical characterization of diverse Env trimers. (**A**) BN-PAGE of ExpiCHO-expressed, GNL-purified Env proteins for SOSIP, UFO, and UFO-BG trimers derived from ten strains of five subtypes (A, B, C, B/C, and A/E) following SEC on a Superdex 200 16/600 column. (**B**) Thermal stability of eight ExpiCHO-expressed UFO-BG trimers derived from five subtypes by differential scanning calorimetry (DSC). (**C**) Thermal stability of four ExpiCHO-expressed UFO trimers derived from four subtypes by DSC. (**D**) Thermal stability of 293 F-expressed BG505 UFO trimer and DU172.17 UFO-BG trimer by DSC. In (B)-(D), three thermal parameters (T_m, $T_{1/2}$ and T_{onset}) are labeled on the DSC profiles. Data fitting was performed using the standard software provided by the vendor. The raw data and the fitted curve are plotted as black dotted lines and red lines, respectively. (**E**) A CN54 gp140 construct with 14 mutations (termed CN54M14). CN54M14 sequence with the 14 mutations shown in red and lower case, the N332 and SOS mutations in blue, the generic HR1 design ($(GS)_4$) in cyan, and the cleavage site linker ($(G_4S)_2$) in green. SEC profiles of wild-type CN54 SOSIP, CN54M14 UFO, and CN54M14 UFO-BG trimers following transient expression in 1L 293 F cells, GNL purification, and SEC on a Superdex 200 10/300 column. (**F**) SEC profiles of UFO-BG trimers derived from clades B, C, and B/C following transient expression in 1L 293 F cells, GNL purification, and SEC on a Superdex 200 10/300 column.

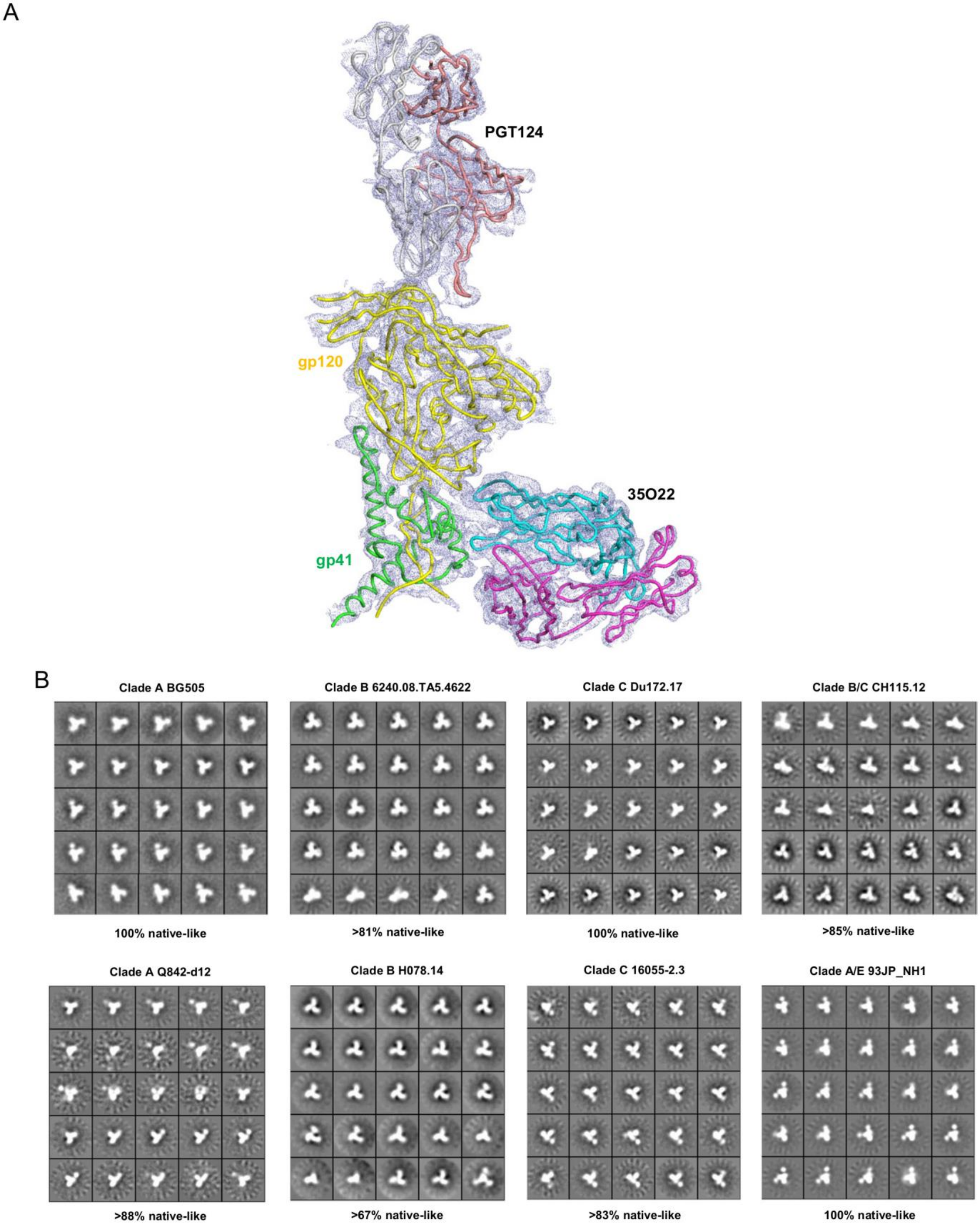
A
PGT124
gp120
35O22
gp41
B
Clade A BG505
100% native-like
Clade B 6240.08.TA5.4622
>81% native-like
Clade C Du172.17
100% native-like
Clade B/C CH115.12
>85% native-like
Clade A Q842-d12
>88% native-like
Clade B H078.14
>67% native-like
Clade C 16055-2.3
>83% native-like
Clade A/E 93JP_NH1
100% native-like

Fig. S3. Structural characterization of diverse UFO-BG trimers. (**A**) Crystal structure of clade B tier 3 H078.14 UFO-BG trimer bound to bNAbs PGT124 and 35O22 at 4.6 Å resolution. A 2Fo-Fc electron density map (contoured at 1.0 **σ**) showing gp120 (in yellow backbone tube model) and $gp41_{ECTO}$ (in green backbone tube model) of the H078.14 UFO-BG protomer in the crystal asymmetric unit bound to PGT124 (grey and pink) and 35O22 (cyan and magenta). (**B**) Negative-stain EM of UFO-BG trimers produced in ExpiCHO cells followed by GNL and SEC purification. The full sets of reference-free 2D class averages are shown for clade A BG505 (tier 2) and Q842-d12 (tier 2), clade B 6240.08.TA5.4622 (tier 2) and H078.14 (tier 2), clade C Du172.17 (tier 2) and 16055-2.3 (tier 2), clade B/C CH115.12 (tier 3) and clade A/E 93JP_NH1. The percentage of native-like trimers is indicated below each panel.

A

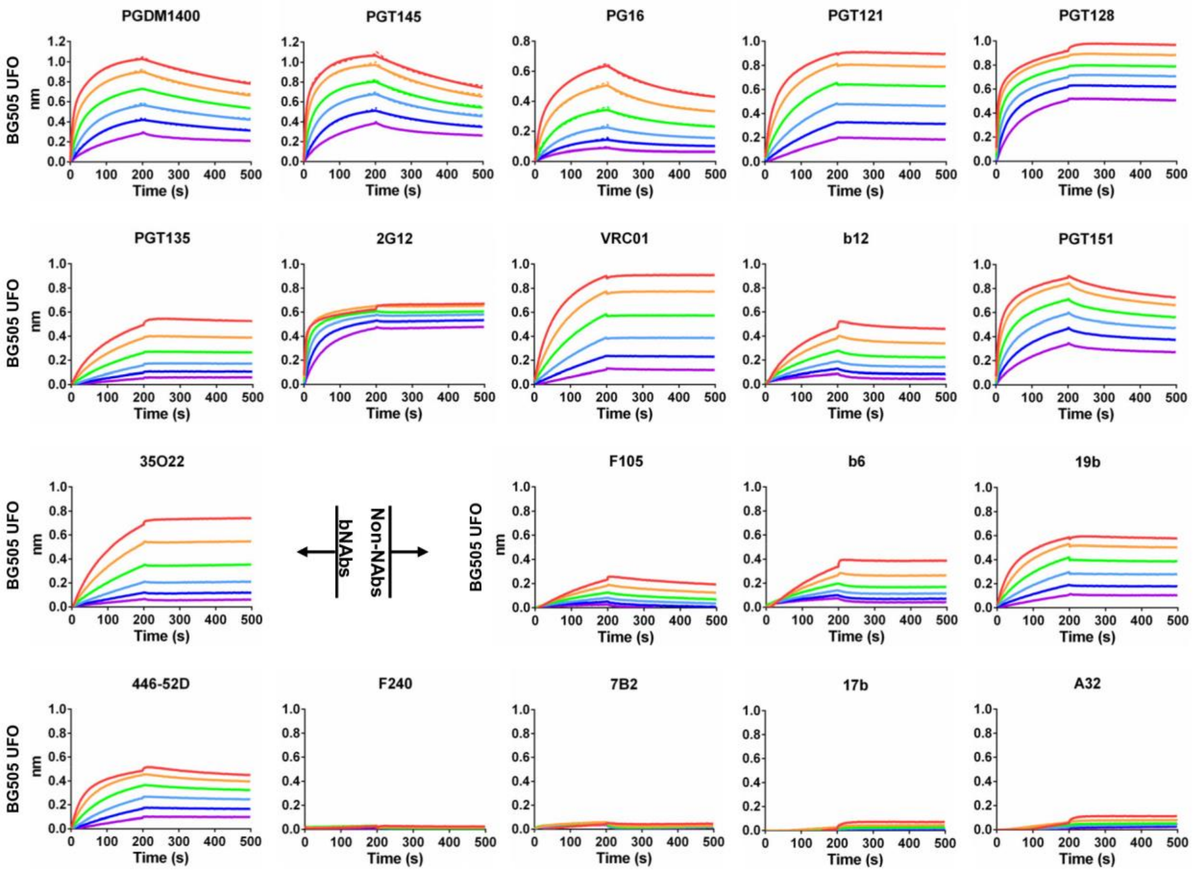

PGDM1400
PGT145
PG16
PGT121
PGT128
PGT135
2G12
VRC01
b12
PGT151
35O22
F105
b6
19b
446-52D
F240
7B2
17b
A32
BG505 UFO
nm
Time (s)
bNAbs
Non-NAbs

B

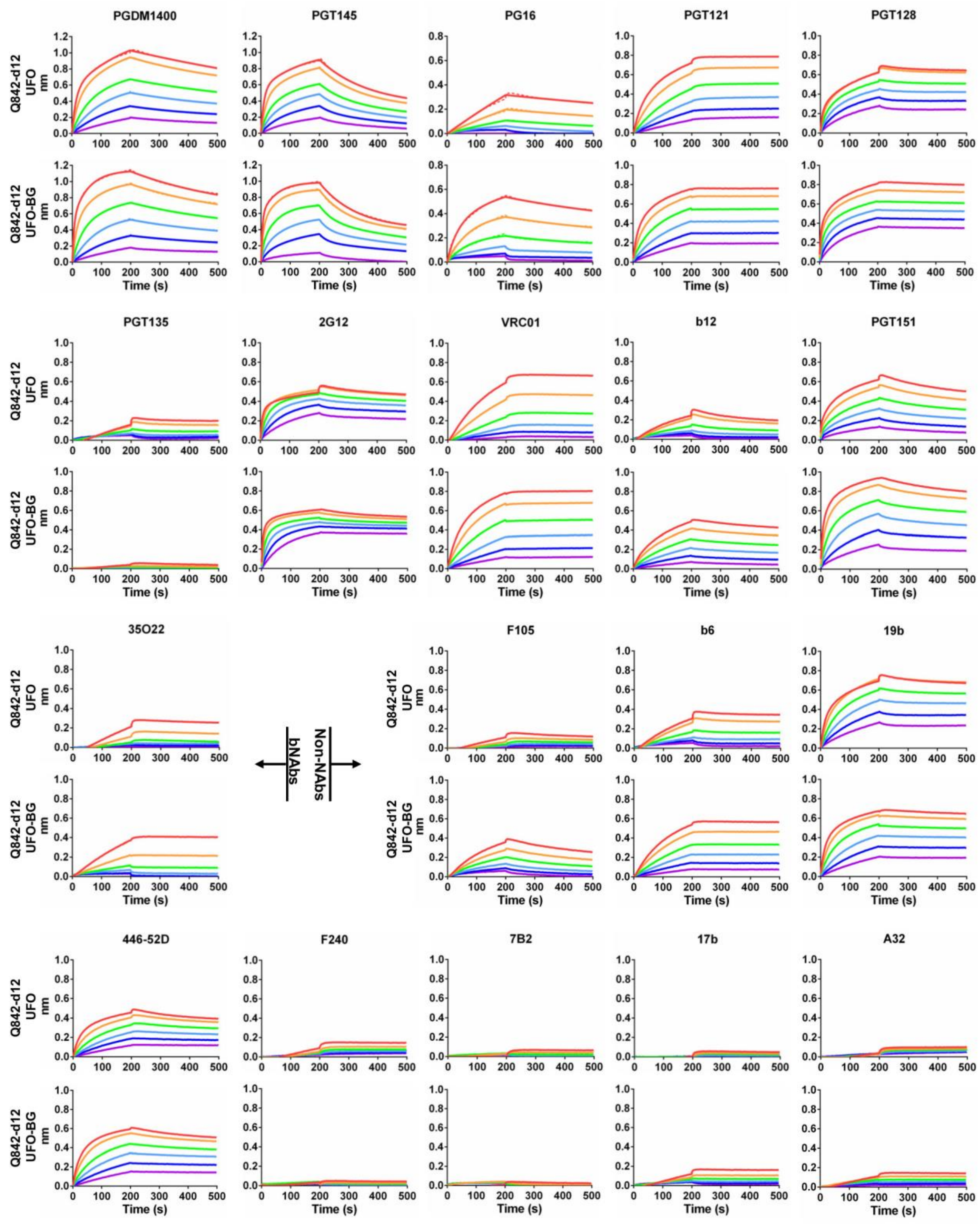

PGDM1400
PGT145
PG16
PGT121
PGT128
PGT135
2G12
VRC01
b12
PGT151
35O22
F105
b6
19b
446-52D
F240
7B2
17b
A32
Q842-d12 UFO nm
Q842-d12 UFO-BG nm
Time (s)
bNAbs
Non-NAbs

C

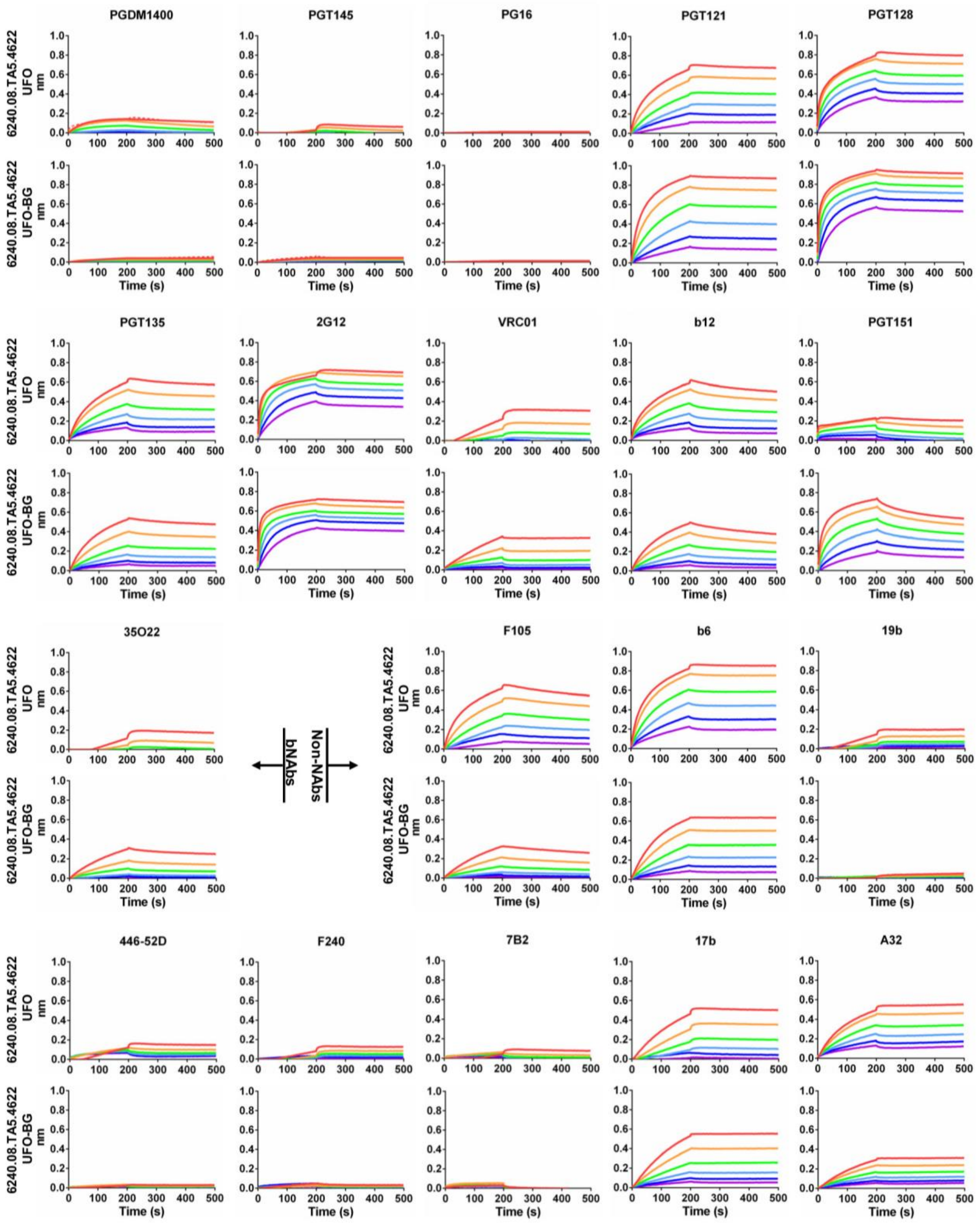

PGDM1400
PGT145
PG16
PGT121
PGT128
PGT135
2G12
VRC01
b12
PGT151
35O22
F105
b6
19b
446-52D
F240
7B2
17b
A32
6240.08.TA5.4622 UFO nm
6240.08.TA5.4622 UFO-BG nm
Time (s)
Non-NAbs
bNAbs

D

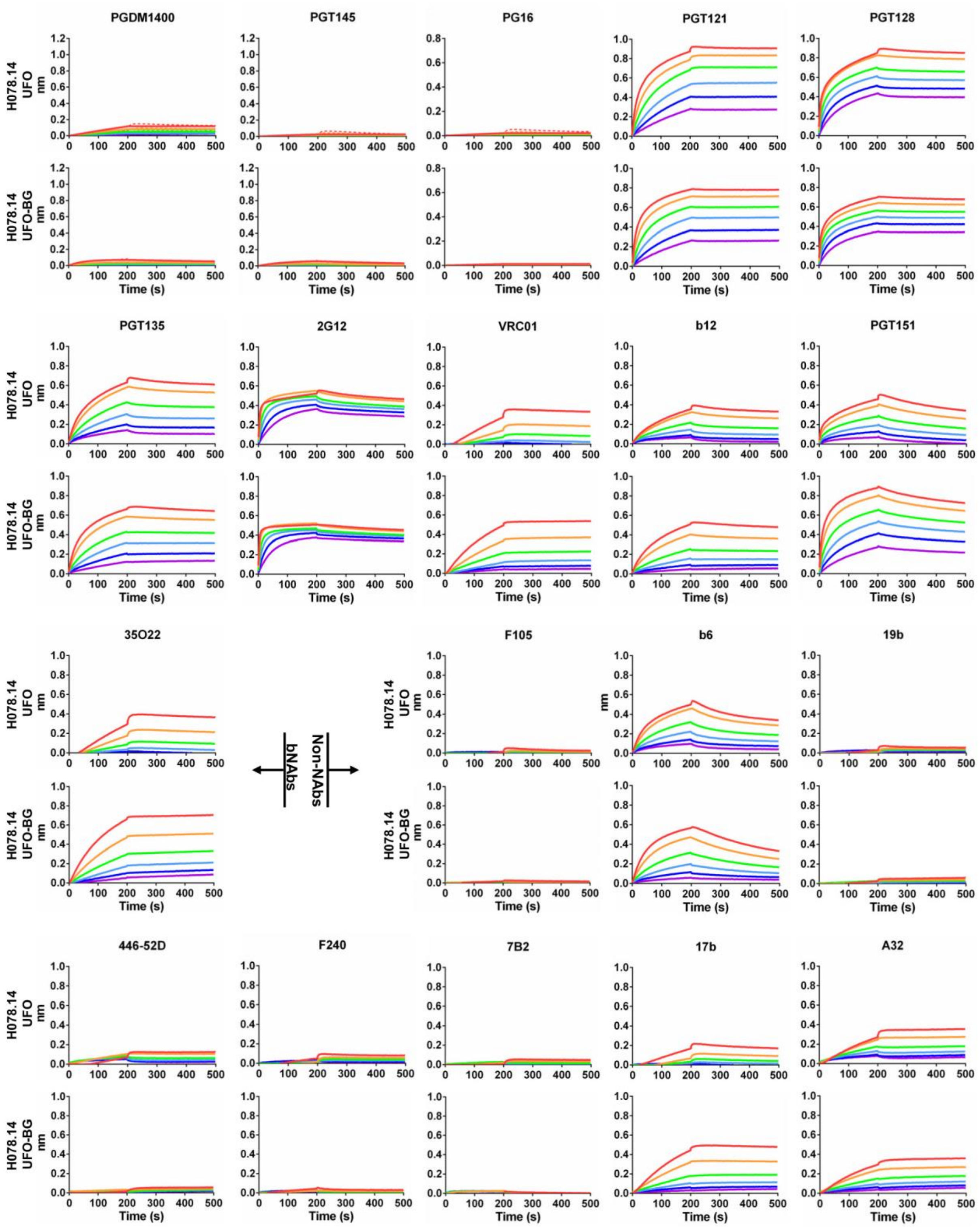

PGDM1400
PGT145
PG16
PGT121
PGT128
PGT135
2G12
VRC01
b12
PGT151
35O22
F105
b6
19b
446-52D
F240
7B2
17b
A32
H078.14 UFO nm
H078.14 UFO-BG nm
Time (s)
bNAbs
Non-NAbs

E

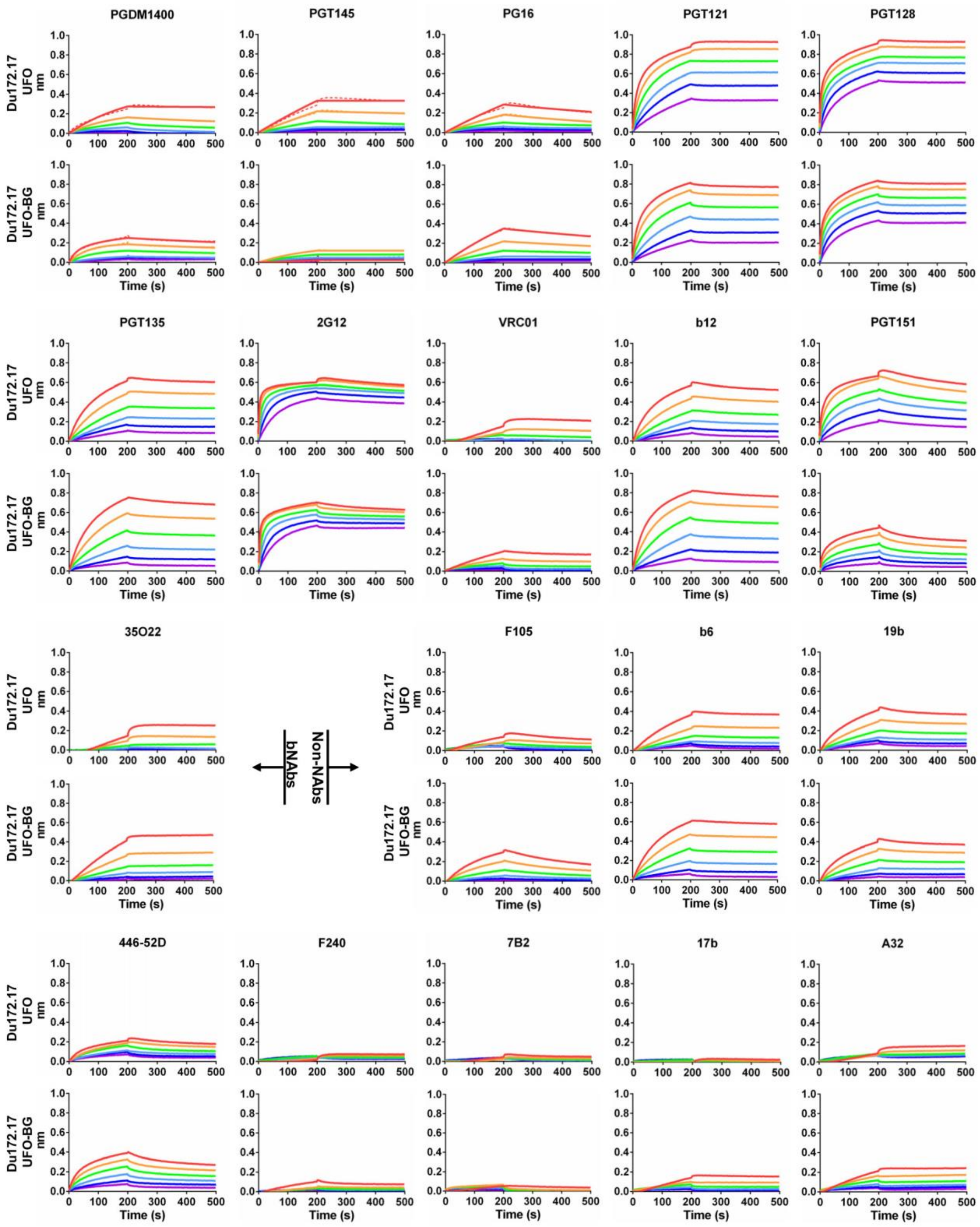

PGDM1400
PGT145
PG16
PGT121
PGT128
PGT135
2G12
VRC01
b12
PGT151
35O22
F105
b6
19b
446-52D
F240
7B2
17b
A32
Du172.17 UFO nm
Du172.17 UFO-BG nm
Time (s)
bNAbs
Non-NAbs

F

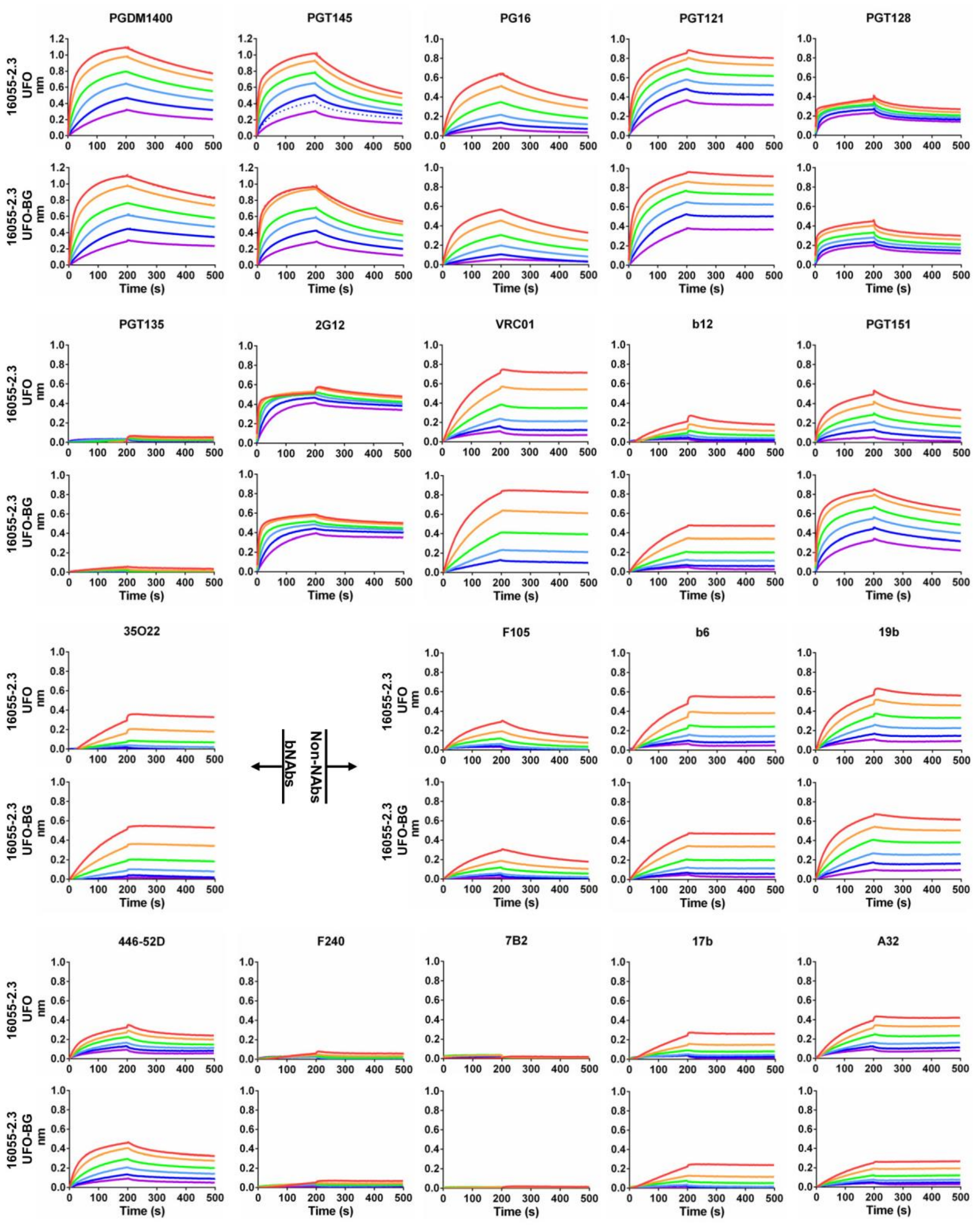
PGDM1400
PGT145
PG16
PGT121
PGT128
PGT135
2G12
VRC01
b12
PGT151
35O22
F105
b6
19b
446-52D
F240
7B2
17b
A32
16055-2.3 UFO nm
16055-2.3 UFO-BG nm
Time (s)
Non-NAbs
bNAbs

G

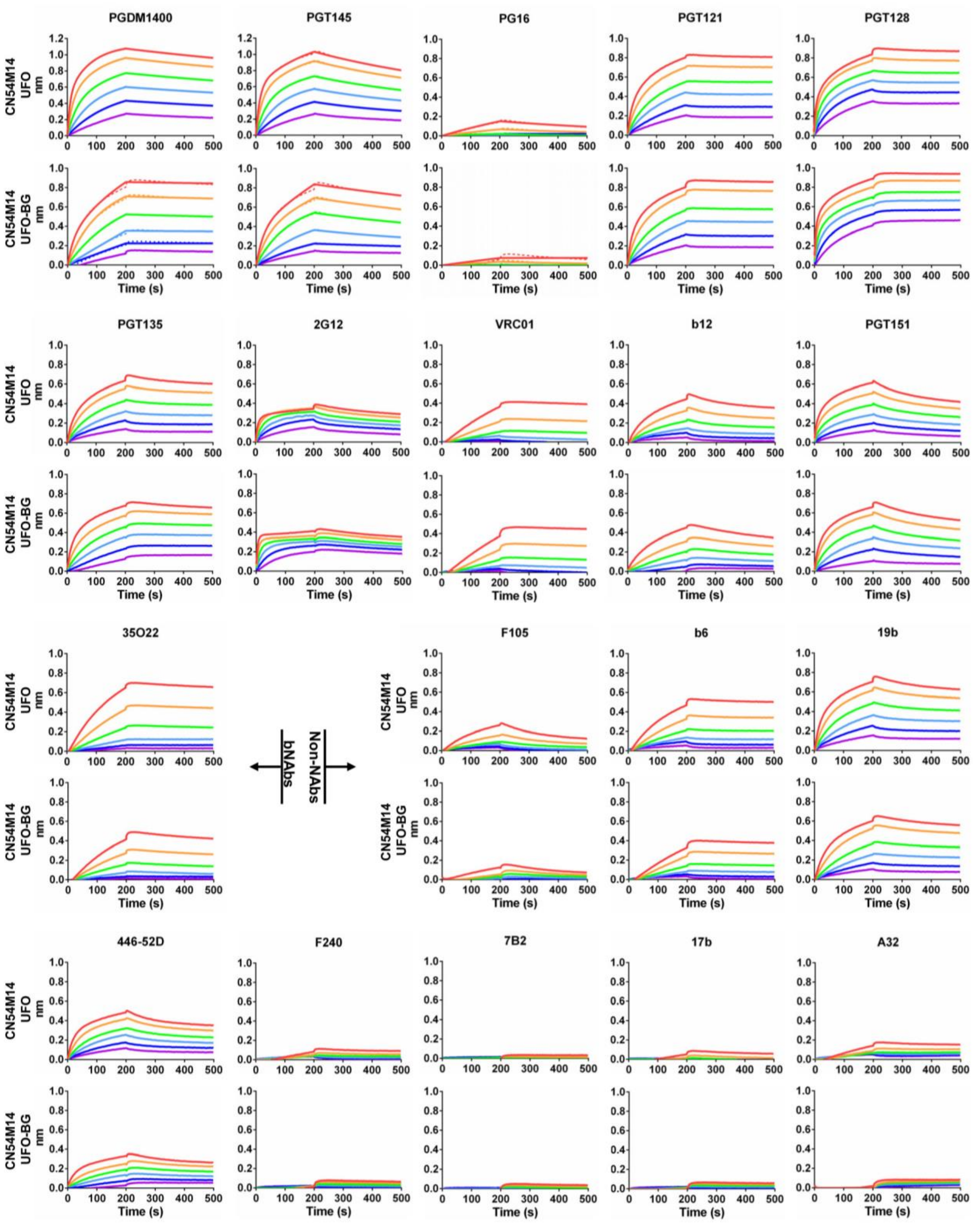
PGDM1400
PGT145
PG16
PGT121
PGT128
PGT135
2G12
VRC01
b12
PGT151
35O22
F105
b6
19b
446-52D
F240
7B2
17b
A32
CN54M14 UFO nm
CN54M14 UFO-BG nm
Time (s)
bNAbs
Non-NAbs

H

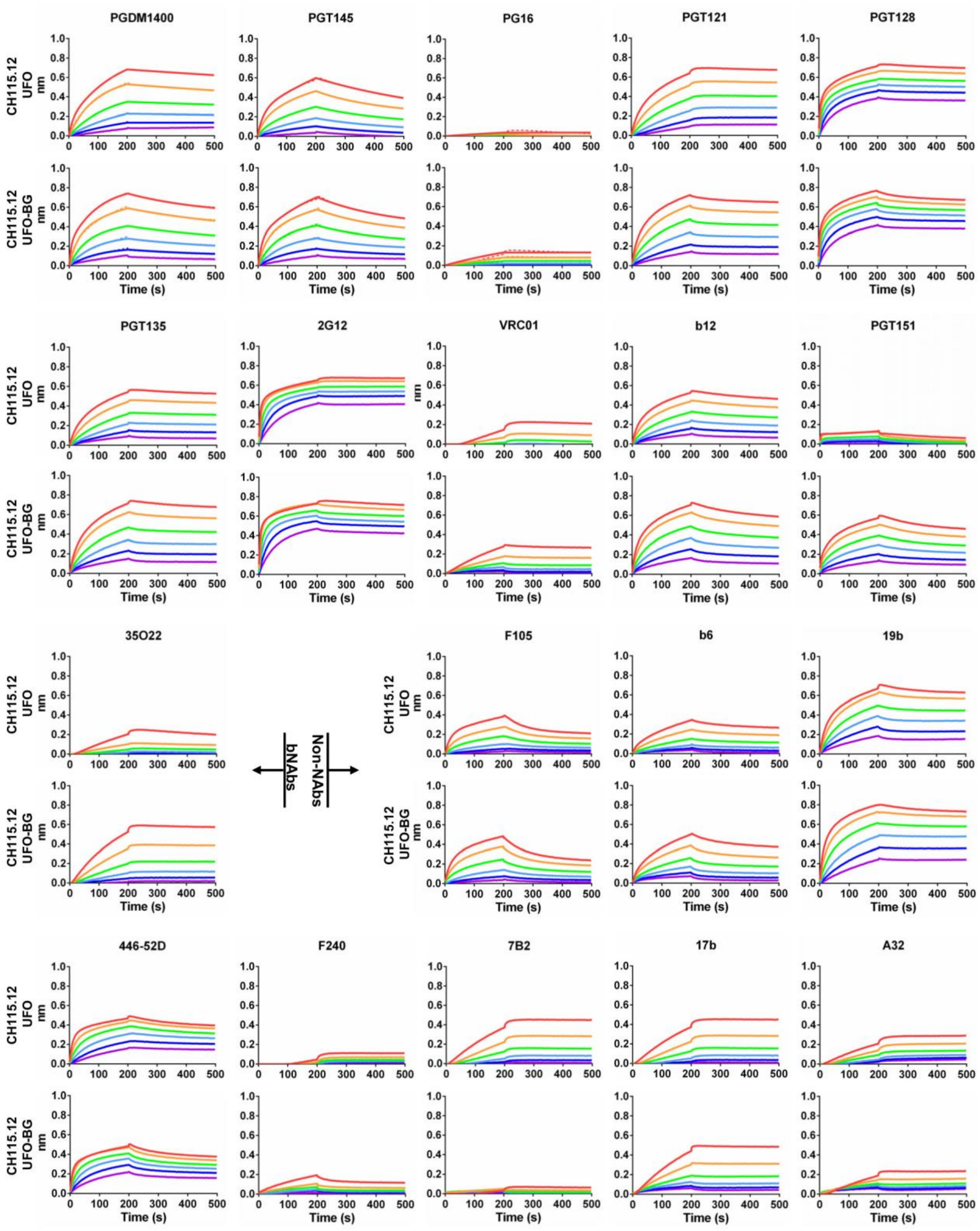
PGDM1400
PGT145
PG16
PGT121
PGT128
PGT135
2G12
VRC01
b12
PGT151
35O22
F105
b6
19b
446-52D
F240
7B2
17b
A32
CH115.12 UFO nm
CH115.12 UFO-BG nm
Time (s)
Non-NAbs
bNAbs

I

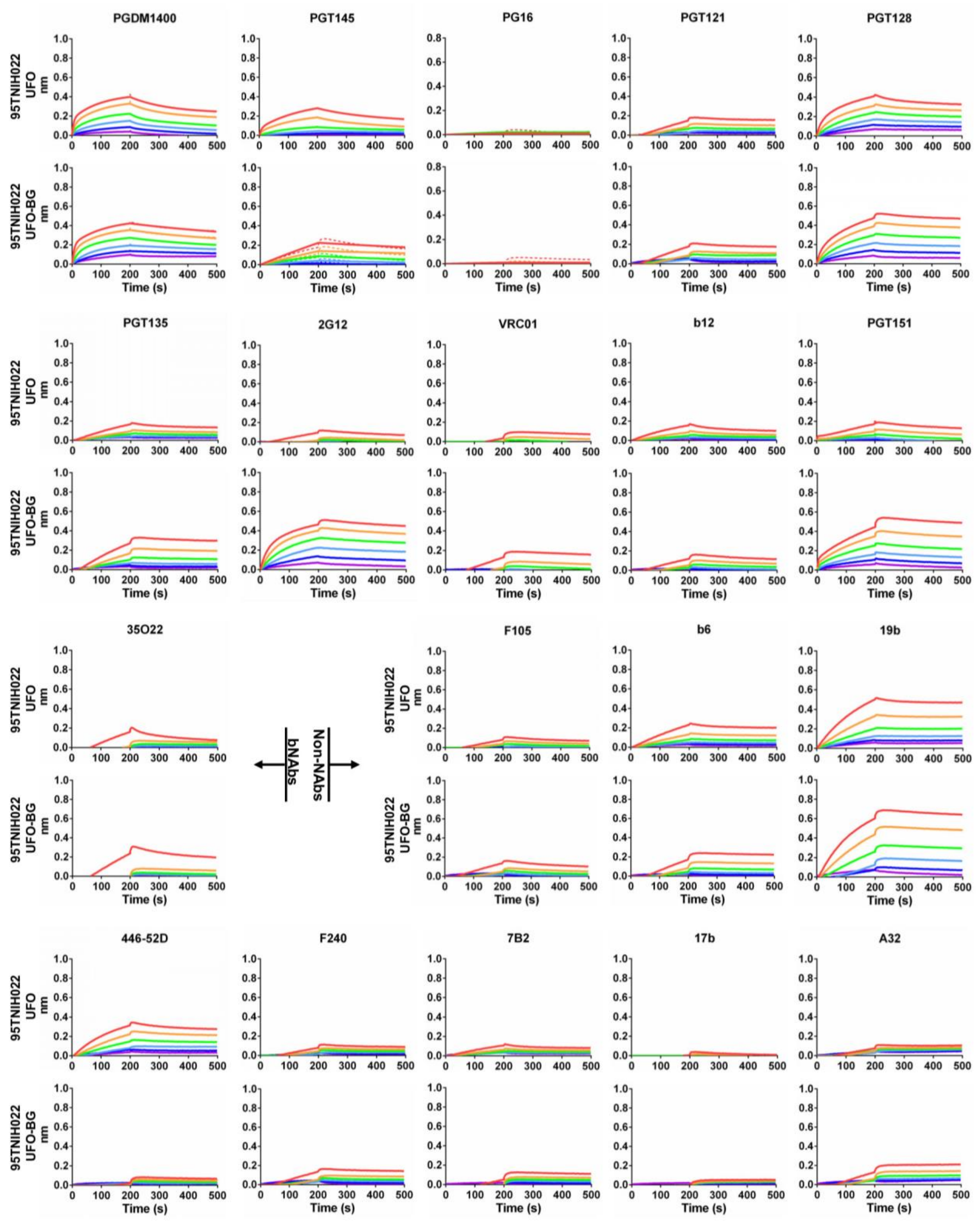
PGDM1400
PGT145
PG16
PGT121
PGT128
PGT135
2G12
VRC01
b12
PGT151
35O22
F105
b6
19b
446-52D
F240
7B2
17b
A32
95TNIH022 UFO nm
95TNIH022 UFO-BG nm
Time (s)
bNAbs
Non-NAbs

J

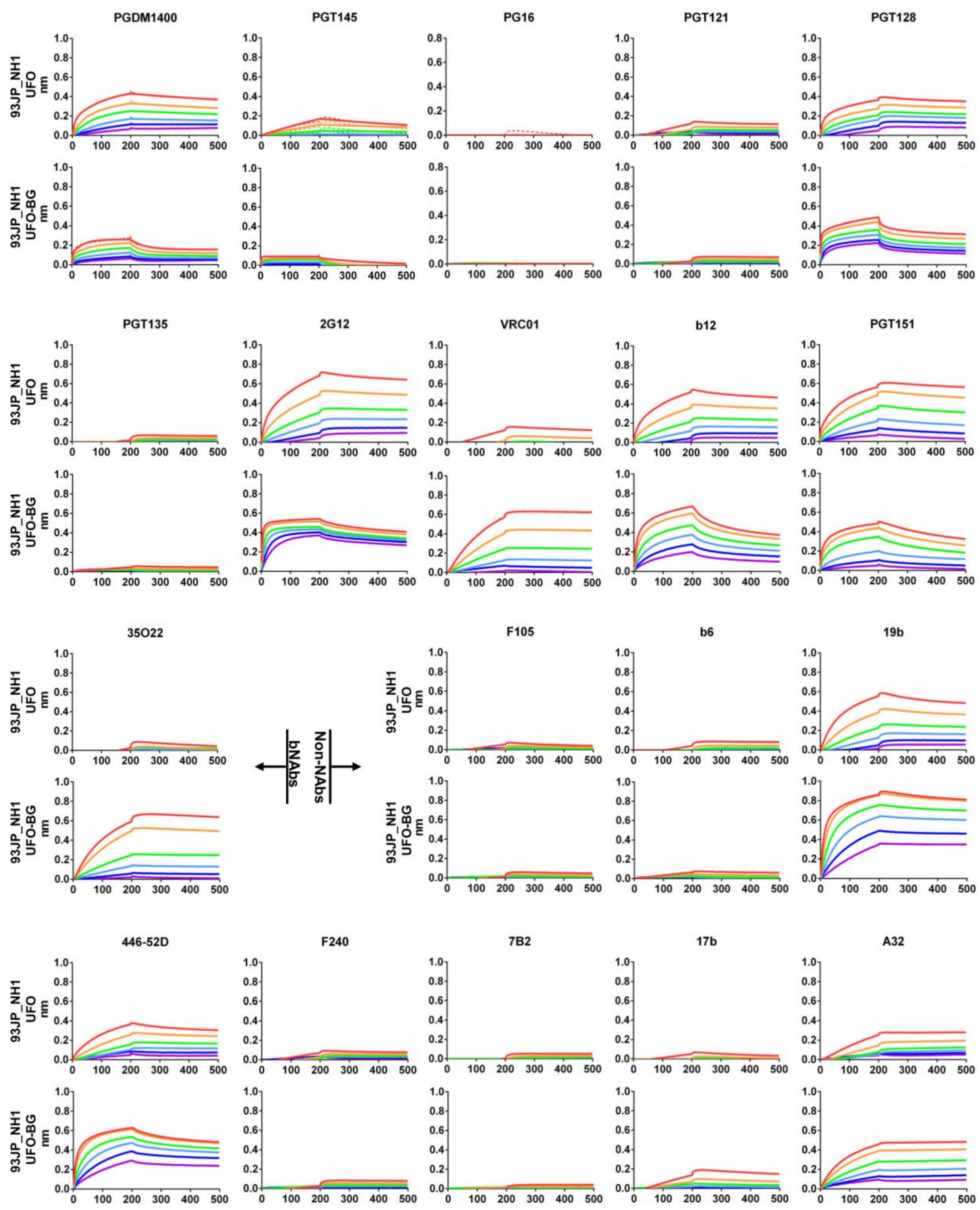

PGDM1400
PGT145
PG16
PGT121
PGT128
PGT135
2G12
VRC01
b12
PGT151
35O22
F105
b6
19b
446-52D
F240
7B2
17b
A32
93JP_NH1 UFO nm
93JP_NH1 UFO-BG nm
Non-NAbs
bNAbs

K

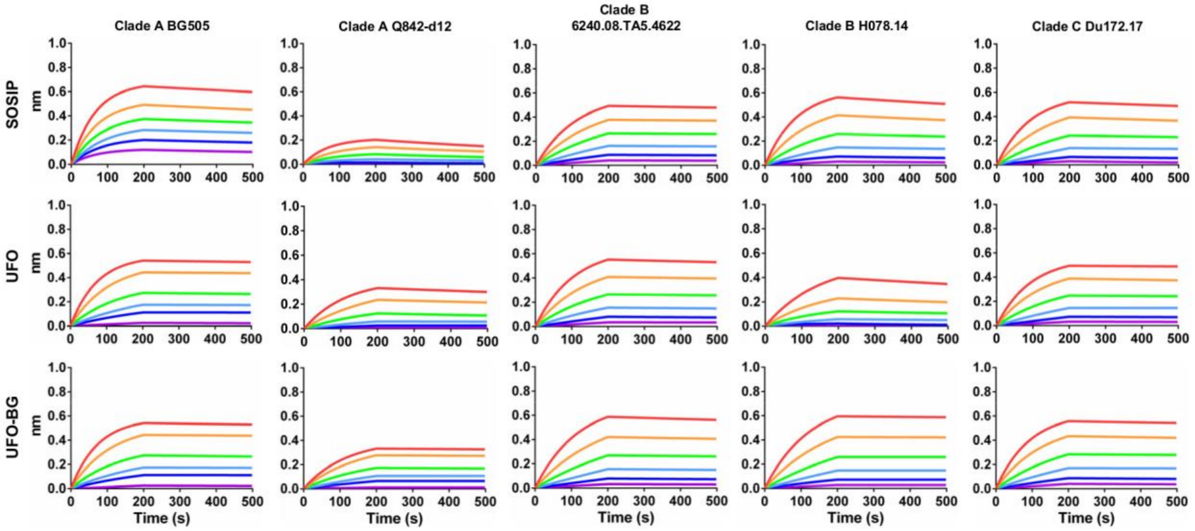

Fig. S4. Antigenic profiles of UFO and UFO-BG trimers derived from 10 strains of five subtypes assessed against a panel of 11 bNAbs, 8 non-NAbs, and CD4-Ig. Sensorgrams were obtained from an Octet RED96 instrument using a titration series of six trimer concentrations (200-6.25 nM by 2-fold dilution). The strains tested for antibody binding include (**A**) clade A BG505 (tier 2), (**B**) clade A Q842-d12 (tier 2), (**C**) clade B 6240.08.TA5.4622 (tier 2), (**D**) clade B H078.14 (tier 2), (**E**) clade C Du172.17 (tier 2), (**F**) clade C 16055-2.3 (tier 2), (**G**) clade B/C CN54 with 14 mutations (CN54M14, see fig. S2E), (**H**) clade B/C CH115.12 (tier 3), (**I**) clade A/E 95TNIH022, and (**J**) clade A/E 93JP_NH1. (**K**) CD4-Ig binding to SOSIP, UFO, and UFO-BG trimers measured for five selected Envs of clades A, B, and C. BLI was performed using the same protocol as described above for antibodies.

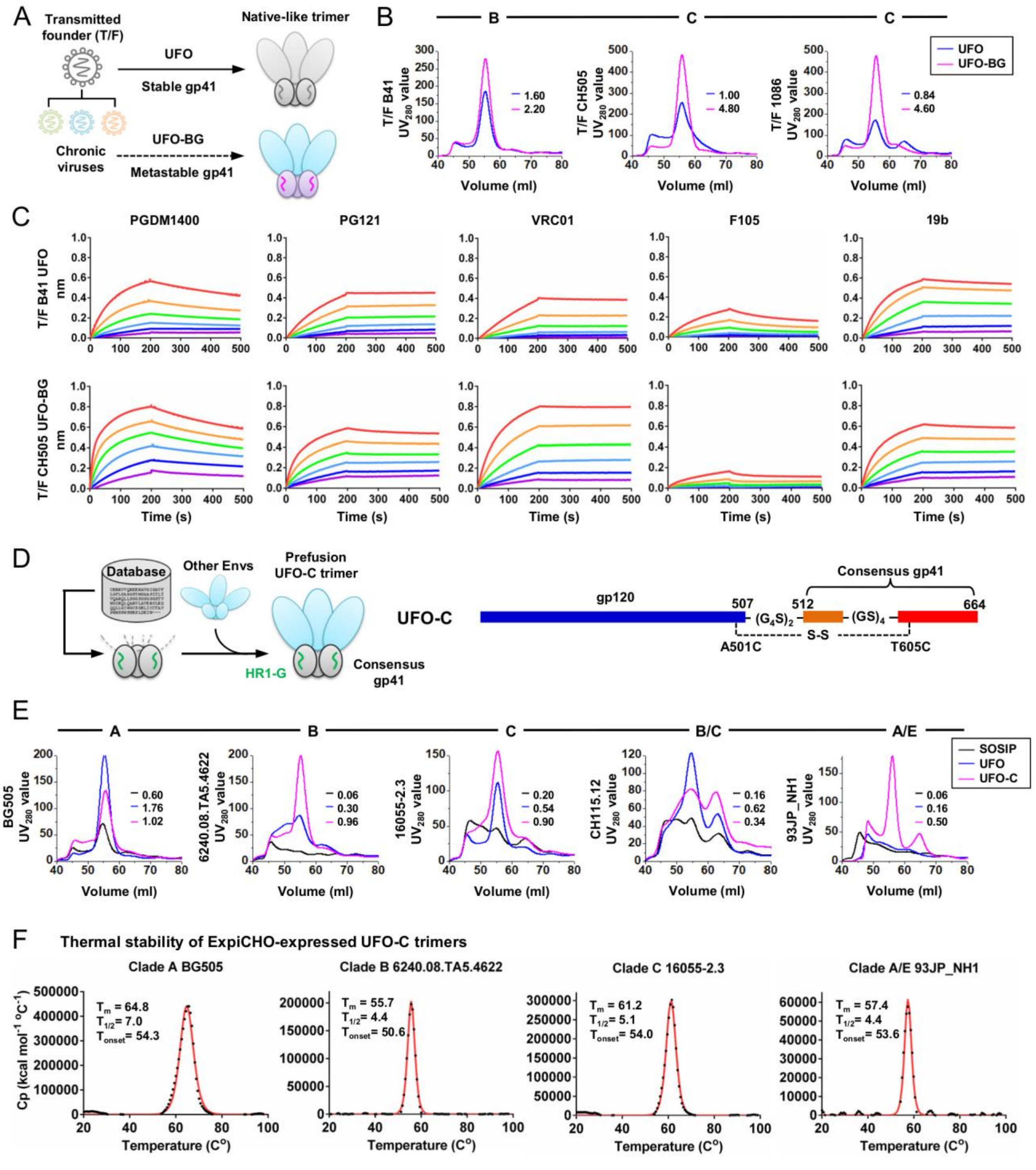

A
Transmitted founder (T/F)
Native-like trimer
UFO
Stable gp41
Chronic viruses
UFO-BG
Metastable gp41
B
B
C
C
UFO
UFO-BG
T/F B41 UV280 value
T/F CH505 UV280 value
T/F 1086 UV280 value
1.60
2.20
1.00
4.80
0.84
4.60
Volume (ml)
C
PGDM1400
PG121
VRC01
F105
19b
T/F B41 UFO nm
T/F CH505 UFO-BG nm
Time (s)
D
Database
Other Envs
Prefusion UFO-C trimer
HR1-G
Consensus gp41
UFO-C
gp120
507
(G4S)2
512
(GS)4
664
A501C
S-S
T605C
E
A
B
C
B/C
A/E
SOSIP
UFO
UFO-C
BG505 UV280 value
6240.08.TA5.4622 UV280 value
16055-2.3 UV280 value
CH115.12 UV280 value
93JP_NH1 UV280 value
0.60
1.76
1.02
0.06
0.30
0.96
0.20
0.54
0.90
0.16
0.62
0.34
0.06
0.16
0.50
Volume (ml)
F
Thermal stability of ExpiCHO-expressed UFO-C trimers
Clade A BG505
Clade B 6240.08.TA5.4622
Clade C 16055-2.3
Clade A/E 93JP_NH1
Tm = 64.8
T1/2= 7.0
Tonset= 54.3
Tm = 55.7
T1/2= 4.4
Tonset= 50.6
Tm = 61.2
T1/2= 5.1
Tonset= 54.0
Tm = 57.4
T1/2= 4.4
Tonset= 53.6
Cp (kcal mol-1 °C-1)
Temperature (C°)

G

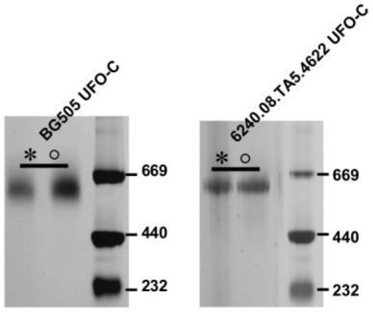

H

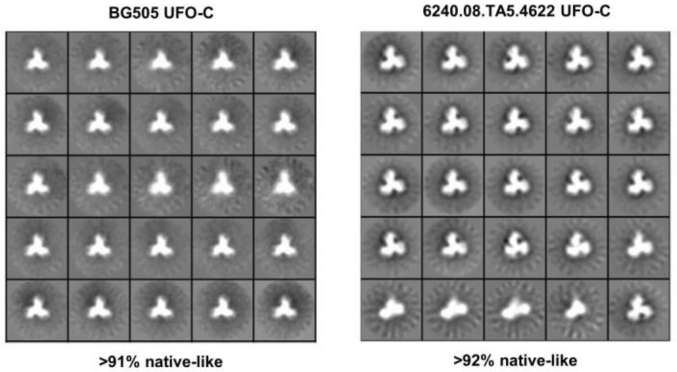

I

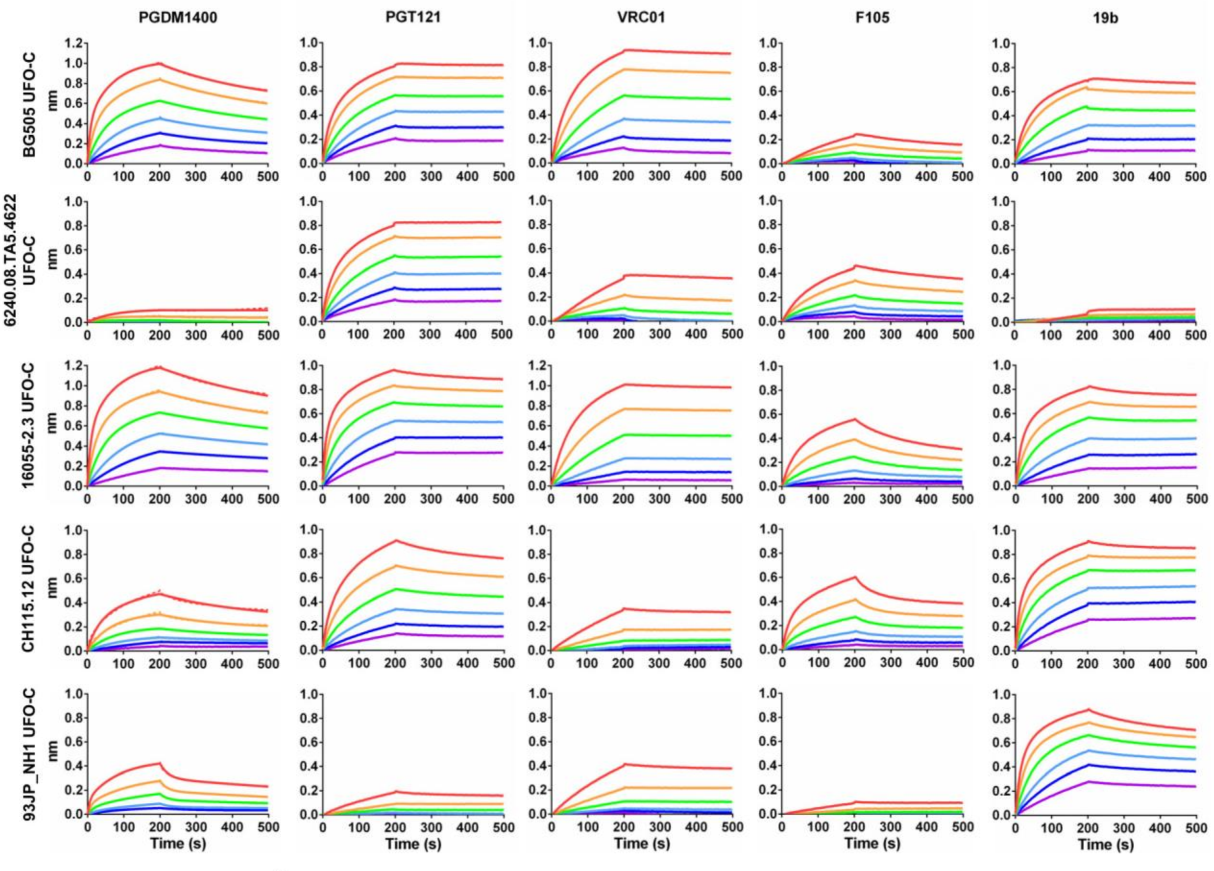

	BG505	6240.08.TA5.4622	16055-2.3	CH115.12	93JP_NH1
PGDM1400	1.0	0.1	1.2	0.5	0.4
PGT121	0.8	0.8	1.0	0.9	0.4
VRC01	0.9	0.4	1.0	0.4	0.2
F105	0.2	0.5	0.6	0.6	0.1
19b	0.7	0.1	0.8	0.9	0.9

Fig. S5. Evolutionary root of metastability and design of UFO-C trimers containing a database-derived ancestral gp41$_{ECTO}$. (**A**) Connection between HIV-1 evolution and Env metastability. (**B**) SEC profiles of UFO and UFO-BG trimers derived from three transmitted/founder (T/F) viruses, including clade B B41, clade C CH505, and clade C 1086. The yield (mg) of SEC-purified trimer protein (fractions corresponding to 53-57ml) obtained from 100-ml ExpiCHO expression is listed for both trimer designs. (**C**) Antigenic profiles of B41 UFO and CH505 UFO-BG trimers measured against three bNAbs and two non-NAbs. Sensorgrams were obtained on an Octet RED96 using a trimer titration series of six concentrations (200-6.25 nM by two-fold dilution). (**D**) Design (left) and schematic representation (right) of the UFO-C trimers. As shown on the left, a database-derived consensus gp41$_{ECTO}$ in conjunction of a generic UFO design is used to stabilize gp120s from diverse HIV-1 Envs in a hybrid form of gp140 trimer designated UFO-C. The generic HR1 redesign, an 8-aa $(GS)_4$ linker, is highlighted in green. (**E**) SEC profiles of UFO-C trimers derived from five representative stains of clade A, B, C, B/C, and A/E origins. The yield (mg) of SEC-purified trimer protein (fractions corresponding to 53-57ml) obtained from 100-ml ExpiCHO expression is listed for each of the three trimer designs (SOSIP, UFO, and UFO-C). (**F**) Thermal stability of 4 ExpiCHO-expressed UFO-C trimers derived from four subtypes by differential scanning calorimetry (DSC). Three thermal parameters (T_m, $T_{1/2}$ and T_{onset}) are labeled on the DSC profiles. Data fitting was performed using the standard software provided by the vendor. The raw data and the fitted curve are plotted as black dotted lines and red lines, respectively. (**G**) BN-PAGE of Env protein after GNL purification but prior to SEC and of purified trimer following SEC and BN-PAGE for two UFO-C constructs derived from clade A BG505 and clade B 6240.08.TA5.4622. (**H**) Reference-free 2D class averages derived from negative-stain EM of the BG505 and 6240.08.TA5.4622 UFO-C trimers. Percentage of native-like trimers is indicated below each panel. (**I**) Antigenic profiles of five purified UFO-C trimers measured against three bNAbs and two non-NAbs. Sensorgrams were obtained on an Octet RED96 using a trimer titration series of six concentrations (200-6.25 nM by two-fold dilution). The strains tested here include clade A BG505 (tier 2), clade B 6240.08.TA5.4622 (tier 2), clade C 16055-2.3 (tier 2), clade B/C CH115.12 (tier 3), and clade A/E 93JP_NH1. The peak values at the highest concentration are summarized in a matrix, in which cells are colored in red and green for bNAbs and non-NAbs, respectively. Higher color intensity indicates greater binding signal measured by Octet.

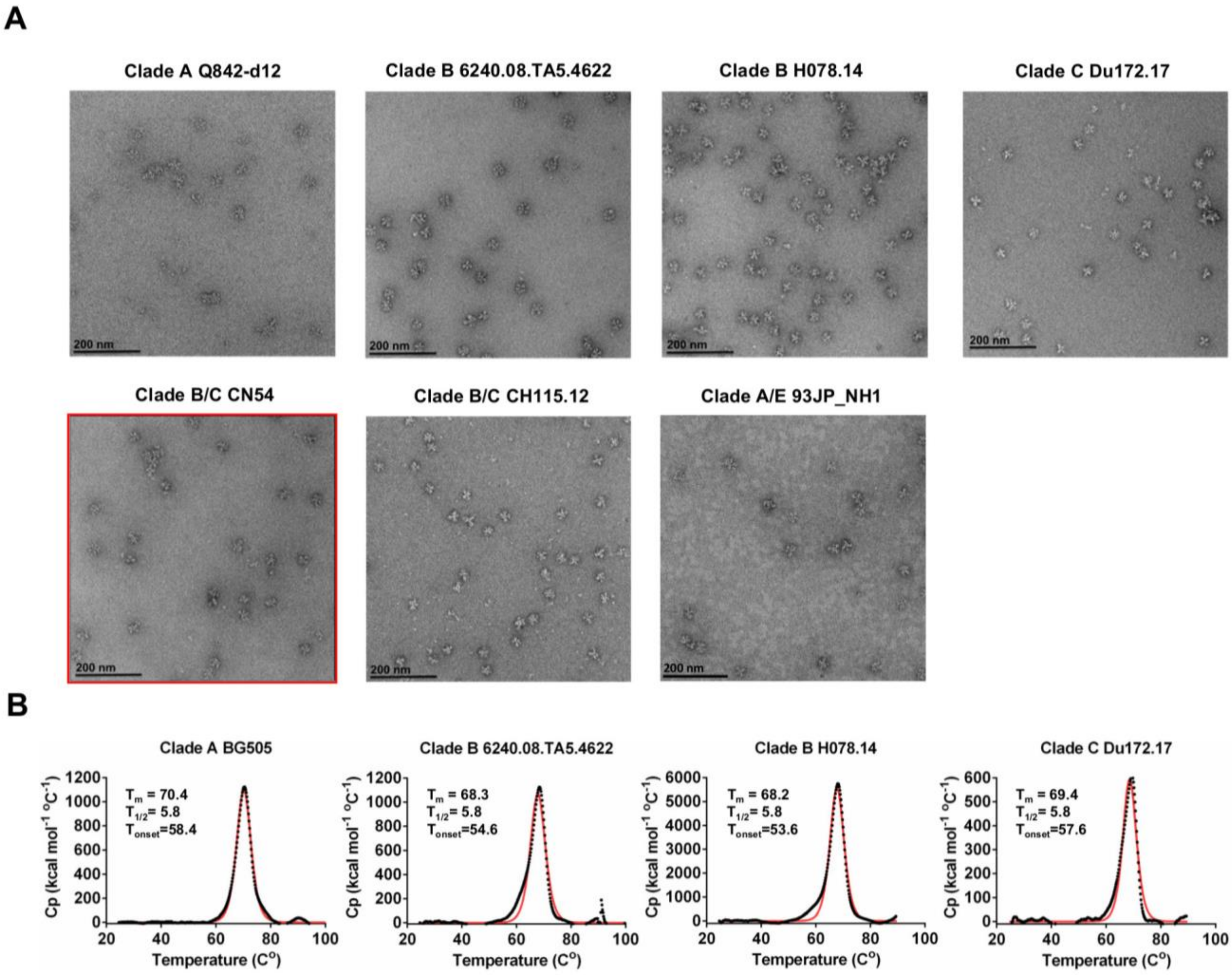
A
Clade A Q842-d12
Clade B 6240.08.TA5.4622
Clade B H078.14
Clade C Du172.17
Clade B/C CN54
Clade B/C CH115.12
Clade A/E 93JP_NH1
200 nm
B
Clade A BG505
Clade B 6240.08.TA5.4622
Clade B H078.14
Clade C Du172.17
Tm = 70.4
T1/2= 5.8
Tonset=58.4
Tm = 68.3
T1/2= 5.8
Tonset=54.6
Tm = 68.2
T1/2= 5.8
Tonset=53.6
Tm = 69.4
T1/2= 5.8
Tonset=57.6
Cp (kcal mol-1 °C-1)
Temperature (C°)

C

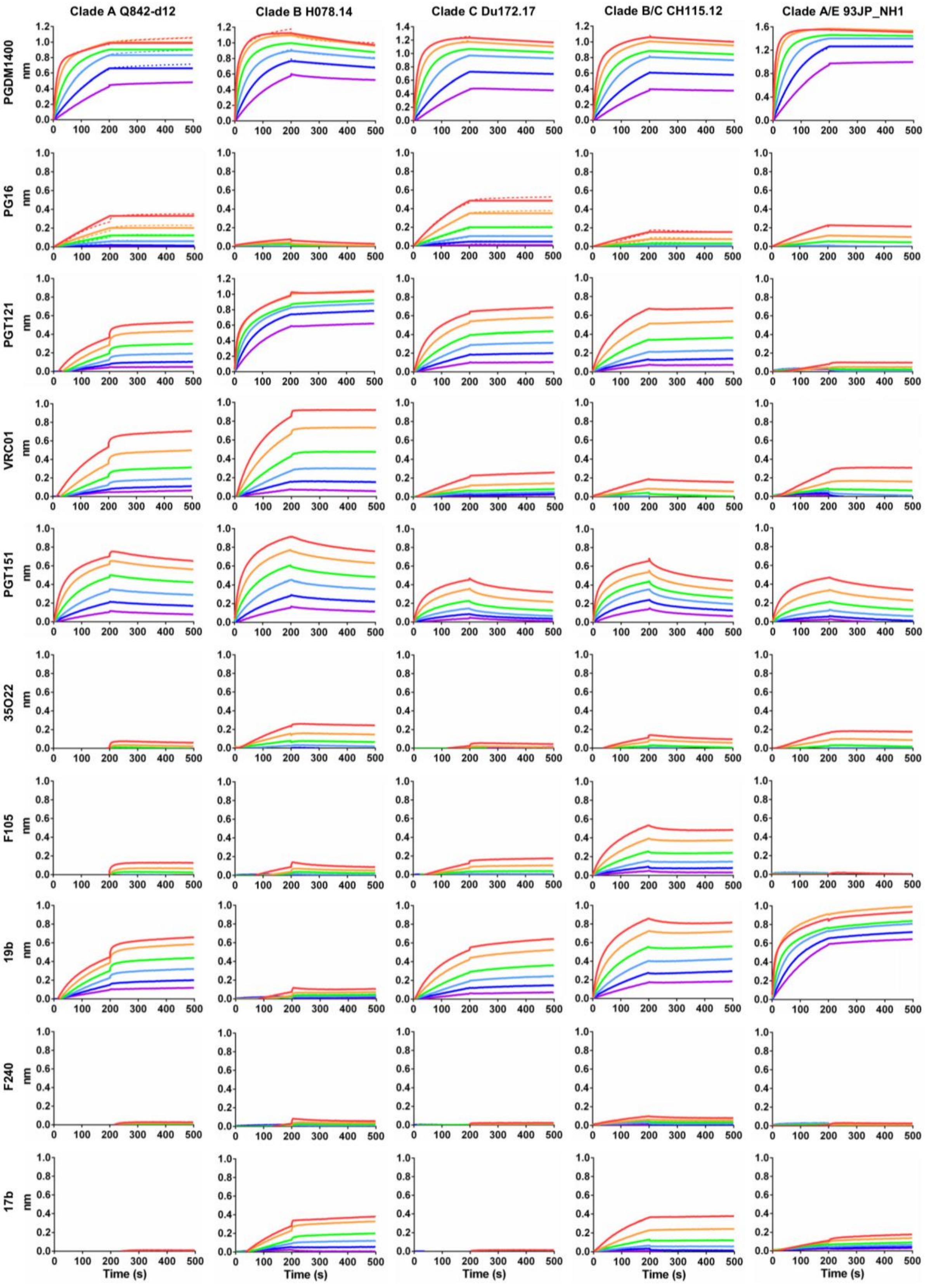
Clade A Q842-d12
Clade B H078.14
Clade C Du172.17
Clade B/C CH115.12
Clade A/E 93JP_NH1
PGDM1400
PG16
PGT121
VRC01
PGT151
35O22
F105
19b
F240
17b
nm
Time (s)

D

Sequence alignment

```
1VLW    MKMEELFKKHKIVAVLRANSVEEAKEKALAVFEGGVHLIEITFTVPDADTVIKELSFLKEKGAIIGAGTVTSVEQCRKAVESGAEFIVSPHLDEEISQFCKEKGVFYMPGVMTPTELVKA
5KP9    MKMEELFKKHKIVAVLRANSVEEAKKKALAVFLGGVHLIEITFTVPDADTVIKELSFLKEMGAIIGAGTVTSVEQCRKAVESGAEFIVSPHLDEEISQFCKEKGVFYMPGVMTPTELVKA
        *************************:****** *************************** ***********************************************************

1VLW    MKLGHTILKLFPGEVVGPQFVKAMKGPFPNVKFVPTGGVNLDNVCEWFKAGVLAVGVGSALVKGTPDEVREKAKAFVEKIRGCTE
5KP9    MKLGHTILKLFPGEVVGPQFVKAMKGPFPNVKFVPTGGVNLDNVCEWFKAGVLAVGVGSALVKGTPVEVAEKAKAFVEKIRGCTE
        ****************************************************************** ** ***************
```

E

```
>1VLW-V1
MKMEELFKKHKIVAVLRANSVEEAKMKALHVFSGGVHLIEITFTVPDADTVIKELSFLKEQGAIIGAGTVTSVEQCRKAVESGAEFIVSPHLDEEISQFCKEKGVFYMPGVMTPTELVKA
MKLGHTILKLFPGEVVGPQFVKAMKGPFPNVKFVPTGGVNLDNVCEWFKAGVLAVGVGSALVKGTWDEVSRKAKAFVEKIRGCTE
>1VLW-V2
MKMEELFKKHKIVAVLRANSVEEAKWKALHVFTGGVHLIEITFTVPDADTVIKELSFLKEQGAIIGAGTVTSVEQCRKAVESGAEFIVSPHLDEEISQFCKEKGVFYMPGVMTPTELVKA
MKLGHTILKLFPGEVVGPQFVKAMKGPFPNVKFVPTGGVNLDNVCEWFKAGVLAVGVGSALVKGTWHEVAAKAKAFVEKIRGCTE
>1VLW-V3
MKMEELFKKHKIVAVLRANSVEEAKMKALHVFTGGVHLIEITFTVPDADTVIKELSFLKEWGAIIGAGTVTSVEQCRKAVESGAEFIVSPHLDEEISQFCKEKGVFYMPGVMTPTELVKA
MKLGHTILKLFPGEVVGPQFVKAMKGPFPNVKFVPTGGVNLDNVCEWFKAGVLAVGVGSALVKGTWDEVAAKAKAFVEKIRGCTE
>1VLW-V4
MKMEELFKKHKIVAVLRANSVEEAKKKALAVFLAGVHLIEITFTVPDADTVIKELSFLKEMGAIIGAGTVTSVEQCRKAVESGAEFIVSPHLDEEISQFCKEKGVFYMPGVMTPTELVKA
MKLGHTILKLFPGEVVGPQFVKAMKGPFPNVKFVPTGGVNLDNVCEWFKAGVLAVGVGSALVKGTVVEVAAKAAAFVEKIRGCTE
>1VLW-V5
MKMEELFKKHKIVAVLRANSVEEAKKKALAVFLGGVHLIEITFTVPDADTVIKELSFLKEMGAIIGAGTVTSVEQCRKAVESGAEFIVSPHLDEEISQFCKEKGVFYMPGVMTPTELVKA
MKLGHTILKLFPGEVVGPQFVKAMKGPFPNVKFVPTGGVNLDNVCEWFKAGVLAVGVGSALVKGTIVEVAAKAAAFVEKIRGCTE
>1VLW-V6
MKMEELFKKHKIVAVLRANSVEEAKKKALAVFLGGVHLIEITFTVPDADTVIKELSFLKEMGAIIGAGTVTSVEQCRKAVESGAEFIVSPHLDEEISQFCKEKGVFYMPGVMTPTELVKA
MKLGHTILKLFPGEVVGPQFVKAMKGPFPNVKFVPTGGVNLDNVCEWFKAGVLAVGVGSALVKGTWVEVAAKAAAFVEKIRGCTE
>1VLW-V7
MKMEELFKKHKIVAVLRANSVEEAKMKALQVFVGGVHLIEITFTVPDADTVIKELSFLKEAGAIIGAGTVTSVEQCRKAVESGAEFIVSPHLDEEISQFCKEKGVFYMPGVMTPTELVKA
MKLGHTILKLFPGEVVGPQFVKAMKGPFPNVKFVPTGGVNLDNVCEWFKAGVLAVGVGSALVKGTLAEVAAKAEAFVEKIRGCTE
>1VLW-V8
MKMEELFKKHKIVAVLRANSVEEAKWKALHVFVGGVHLIEITFTVPDADTVIKELSFLKEAGAIIGAGTVTSVEQCRKAVESGAEFIVSPHLDEEISQFCKEKGVFYMPGVMTPTELVKA
MKLGHTILKLFPGEVVGPQFVKAMKGPFPNVKFVPTGGVNLDNVCEWFKAGVLAVGVGSALVKGTWAEVAAKAKAFVEKIRGCTE
>1VLW-V9
MKMEELFKKHKIVAVLRANSVEEAKMKALAVFVGGVHLIEITFTVPDADTVIKELSFLKELGAIIGAGTVTSVEQCRKAVESGAEFIVSPHLDEEISQFCKEKGVFYMPGVMTPTELVKA
MKLGHTILKLFPGEVVGPQFVKAMKGPFPNVKFVPTGGVNLDNVCEWFKAGVLAVGVGSALVKGTIAEVAAKAAAFVEKIRGCTE
>1VLW-V10
MKMEELFKKHKIVAVLRANSVEEAKMKALAVFYGGVHLIEITFTVPDADTVIKELSFLKEAGAIIGAGTVTSVEQCRKAVESGAEFIVSPHLDEEISQFCKEKGVFYMPGVMTPTELVKA
MKLGHTILKLFPGEVVGPQFVKAMKGPFPNVKFVPTGGVNLDNVCEWFKAGVLAVGVGSALVKGTFVEVAAKAAAFVEKIRGCTE
```

F

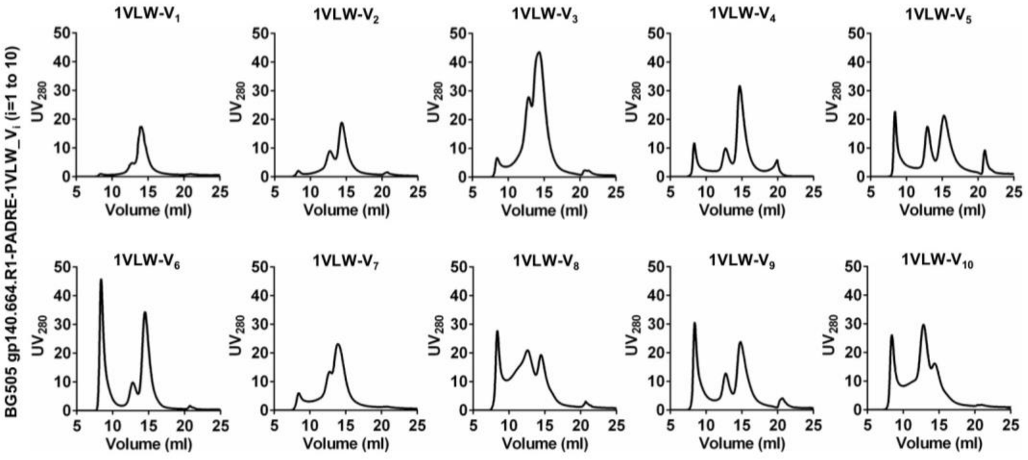

Fig. S6. Characterization of gp41$_{ECTO}$-stabilized trimer-presenting nanoparticles.
(**A**) Micrographs derived from negative-stain EM of UFO-BG-FR nanoparticles of five subtypes. (**B**) Thermal stability of four UFO-BG-FR nanoparticles derived from three subtypes by differential scanning calorimetry (DSC). Three thermal parameters (T_m, $T_{1/2}$ and T_{onset}) are labeled on the DSC profiles. Data fitting was performed using the standard software provided by the vendor. The raw data and the fitted curve are plotted as black dotted lines and red lines, respectively. (**C**) Antigenic profiles of UFO-BG-FR nanoparticles derived from five subtypes evaluated against a panel of six bNAbs and four non-NAbs. Sensorgrams were obtained from an Octet RED96 instrument using a titration series of 6 concentrations (starting at 35 nM by 2-fold dilution). The strains tested here include clade A Q842-d12 (tier 2), clade B H078.14 (tier 2), clade C Du172.17 (tier 2), clade B/C CH115.12 (tier 3), and clade A/E 93JP_NH1. (**D**) Sequence alignment of I3-01 (PDBID: 5KP9) and 2-dehydro-3-deoxyphosphogluconate aldolase/4-hydroxy-2-oxoglutarate aldolase (TM0066) from *Thermotoga maritima* (PDBID: 1VLW), with the five amino acids that differ in the two sequences colored in red and the 11 amino acids at the I3-01 particle-forming interface highlighted in yellow. (**E**) Sequences of ten 1VLW variants (1VLW-V_i, i=1 to 10) by altering amino acids at the corresponding I3-01 particle-forming interface. (**F**) SEC profiles of ten BG505 gp140.664.R1-PADRE-1VLW-V_i constructs obtained from a Superose 6 10/300 GL column. The fusion constructs were expressed in ExpiCHO cells and purified using a 2G12 affinity column.

A

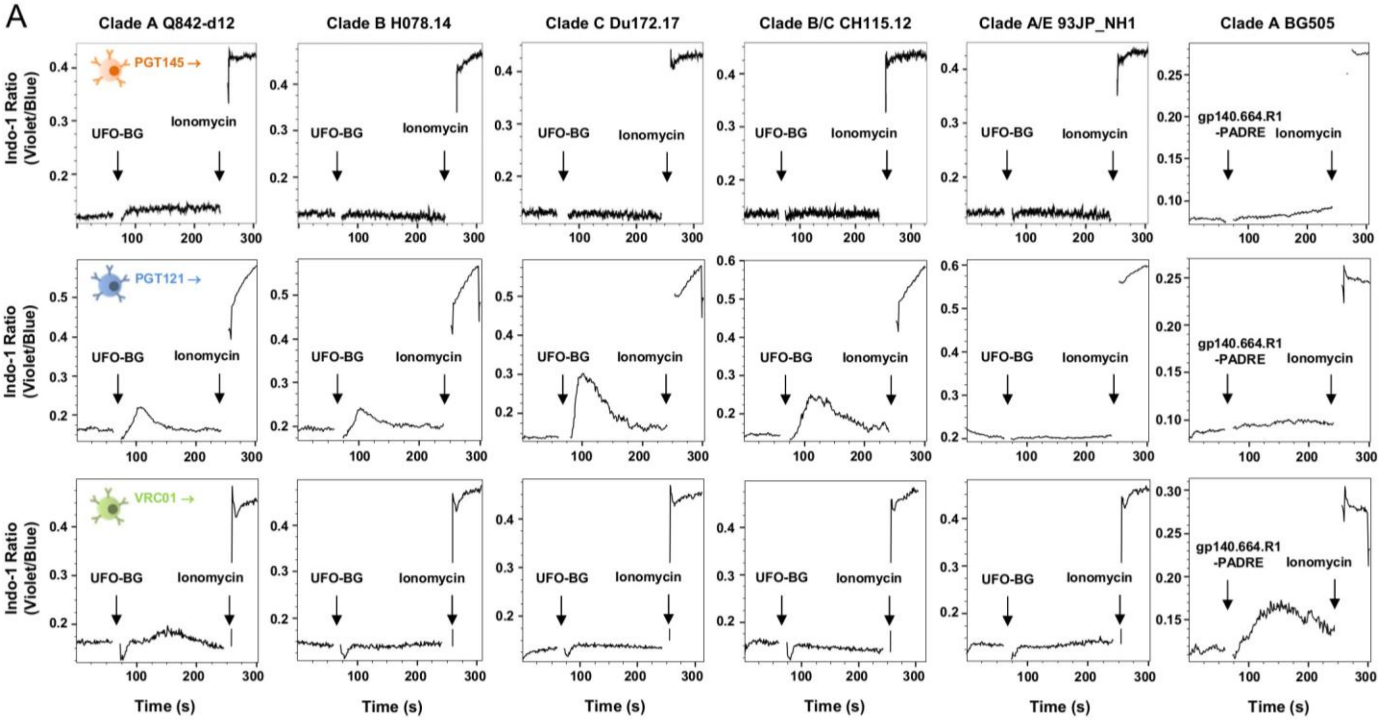

B

gp41$_{ECTO}$-stabilized BG505 trimer and nanoparticle immunogen groups [a]

**Group 1**: gp140.664.R1 (293 F)	**Group 2**: gp140.664.R1 (CHO)	**Group 3**: gp140.681.R1-1NOG (293 F)	**Group 4**: gp140.681.R1-1V6H (293F)	**Group 5**: gp140.681.R1-4FUR
**Group 6**: gp140.664.R1-ferritin (CHO)	**Group 7**: gp140.664.R1-PADRE (CHO)	**Group 8**: gp140.664.R1-D (CHO)	**Group 9**: gp140.664.R1-TpD (CHO)	**Group 10**: gp140.664.R1-PADRE-I3-01 (CHO)
**Group 11**: gp140.664.R1-D-I3-01 (CHO)	**Group 12**: gp140.664.R1-TpD-I3-01 (CHO)	**Group 13**: unimmunized	Trimer	Nanoparticle

[a] All gp41$_{ECTO}$-stabilized trimers and nanoparticles in this mouse immunization were derived from BG505 gp140.664.R1, which contains a redesigned HR1 in gp41$_{ECTO}$. This construct has been previously characterized using biochemical, biophysical, structural, and antigenic approaches (Kong et al. (2016), *Nat. Commun.* 7:12040) and is parental to BG505 UFO and all UFO-BG trimers. In the construct names, R1 stands for HR1 redesign 1 and 664 indicates the amino acid position at which gp140 is truncated (the expression system, 293F or CHO, is indicated in the parentheses).

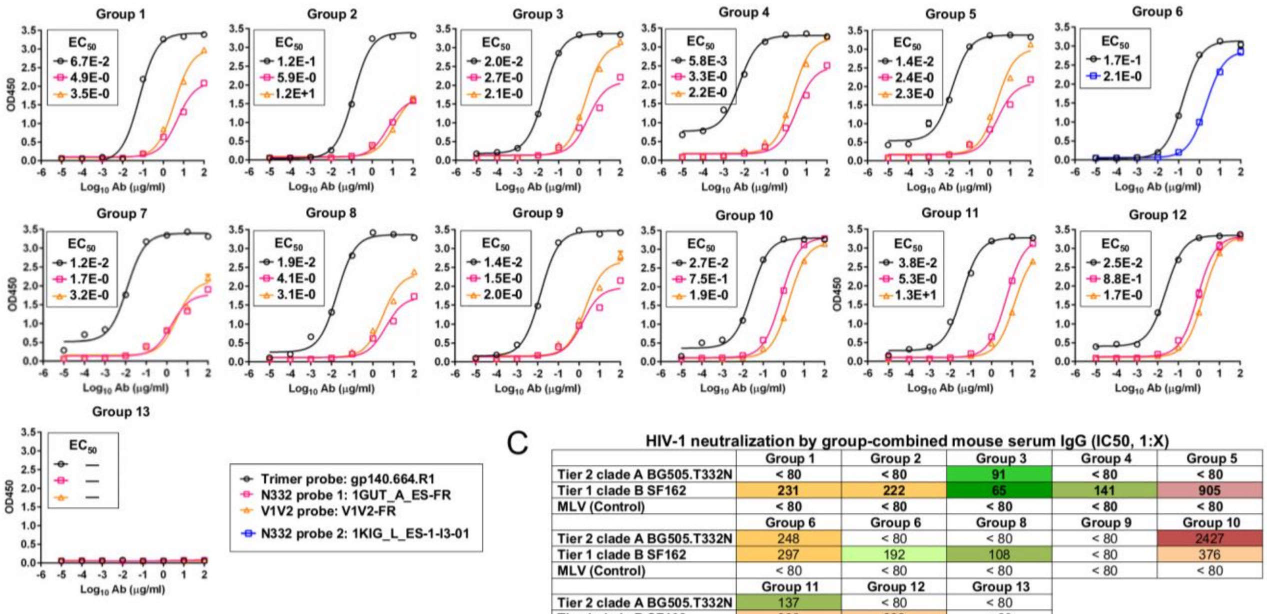

C

HIV-1 neutralization by group-combined mouse serum IgG (IC50, 1:X)

	Group 1	Group 2	Group 3	Group 4	Group 5
**Tier 2 clade A BG505.T332N**	< 80	< 80	91	< 80	< 80
**Tier 1 clade B SF162**	231	222	65	141	905
**MLV (Control)**	< 80	< 80	< 80	< 80	< 80
	**Group 6**	**Group 6**	**Group 8**	**Group 9**	**Group 10**
**Tier 2 clade A BG505.T332N**	248	< 80	< 80	< 80	2427
**Tier 1 clade B SF162**	297	192	108	< 80	376
**MLV (Control)**	< 80	< 80	< 80	< 80	< 80
	**Group 11**	**Group 12**	**Group 13**		
**Tier 2 clade A BG505.T332N**	137	< 80	< 80		
**Tier 1 clade B SF162**	396	229	< 80		
**MLV (Control)**	< 80	< 80	< 80		

D Longitudinal ELISA analysis of rabbit plasma binding to BG505 UFO trimer and an N332 probe

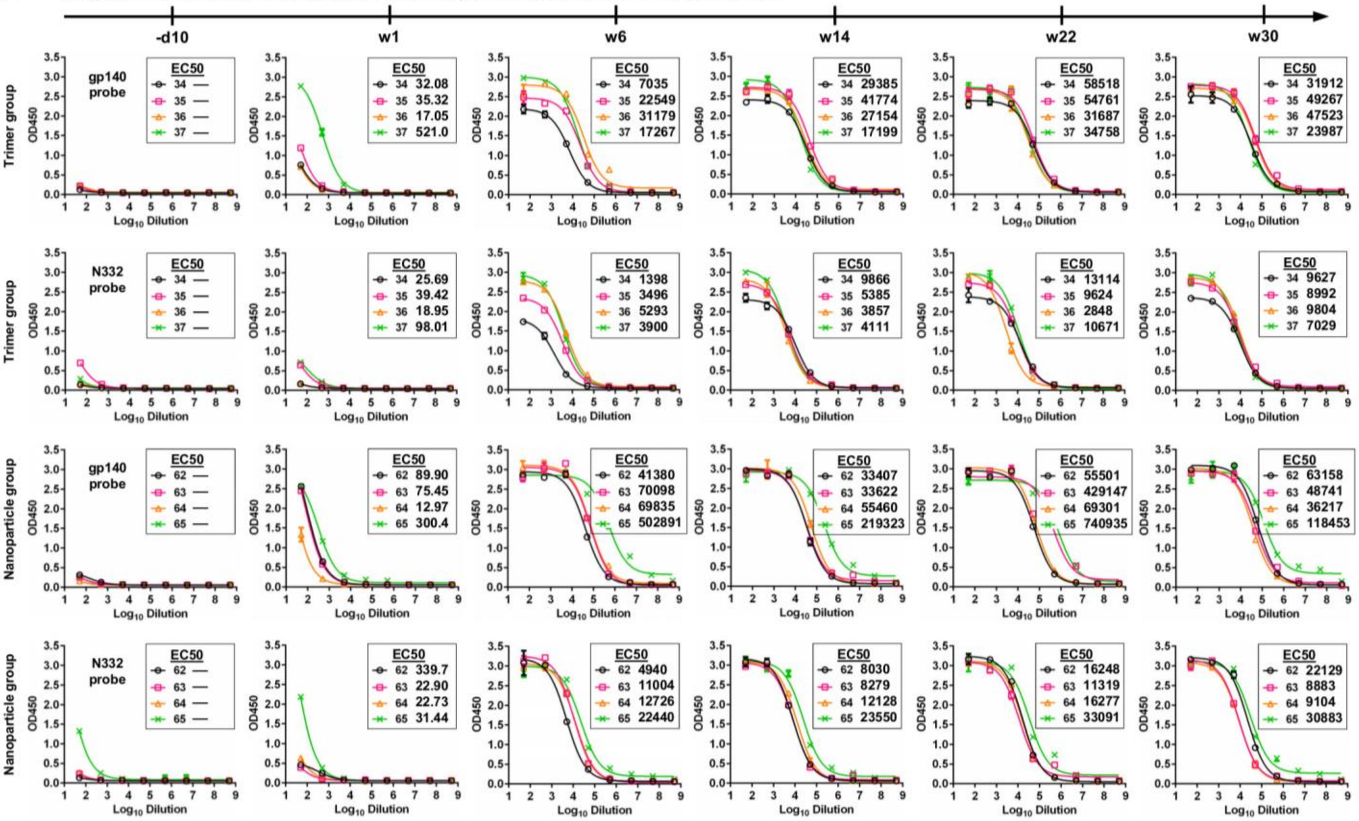

E Longitudinal analysis of rabbit plasma neutralization of clade A tier 2 BG505.T332N and clade B tier 1 SF162

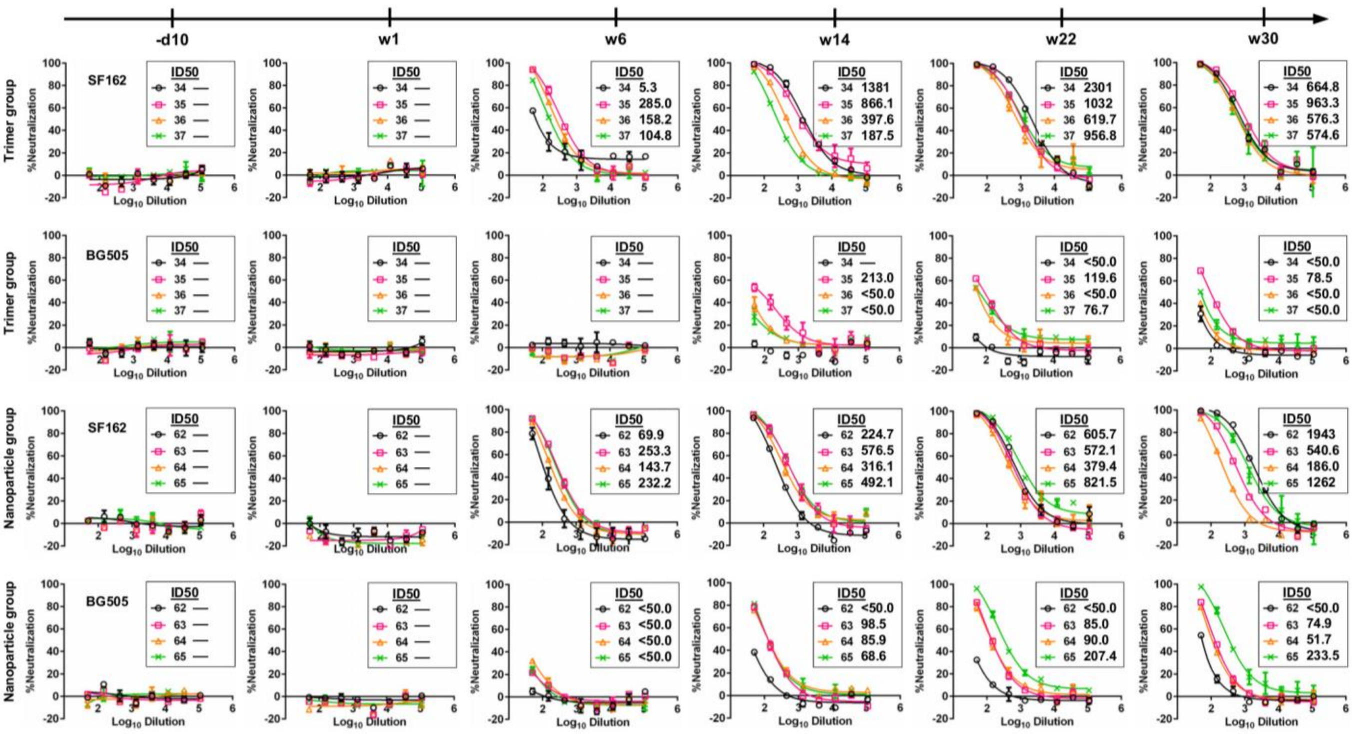

F Neutralization of global panel by rabbit plasma pre-immunization (-d10) and at week 22 (w22) in the trimer group

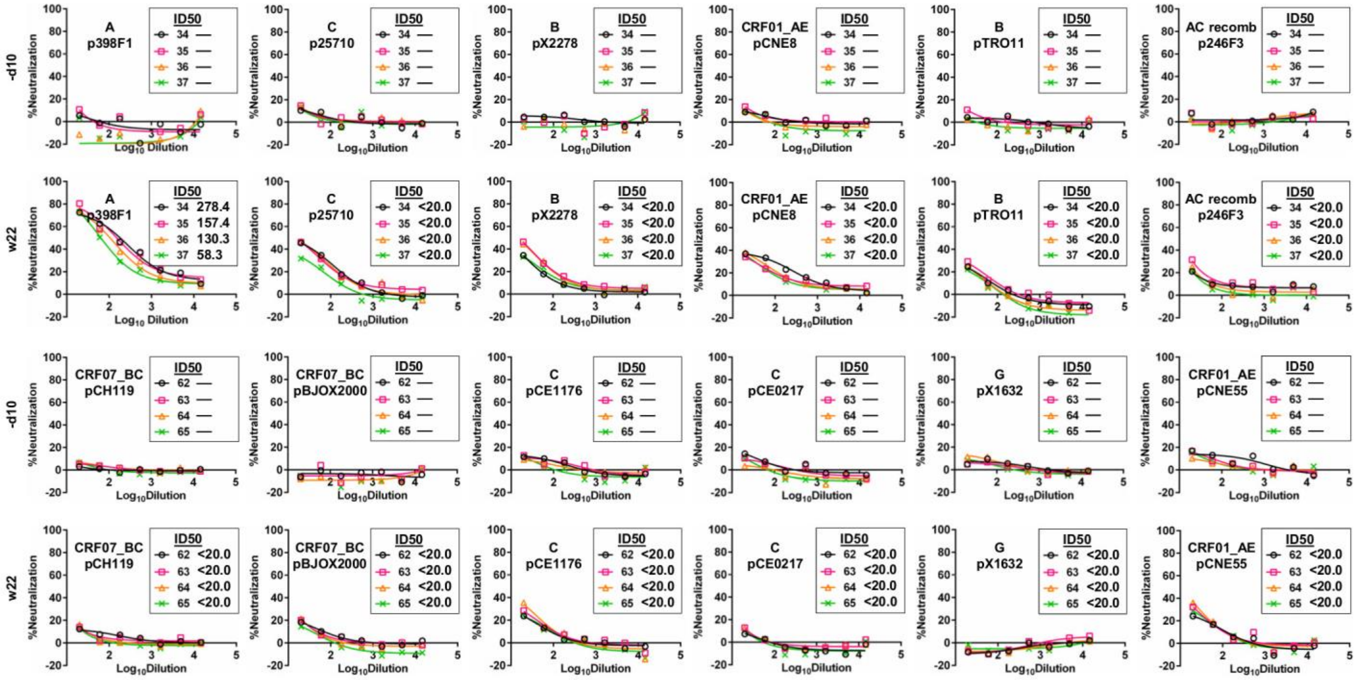

G Neutralization of global panel by rabbit plasma pre-immunization (-d10) and at week 22 (w22) in the ferritin group

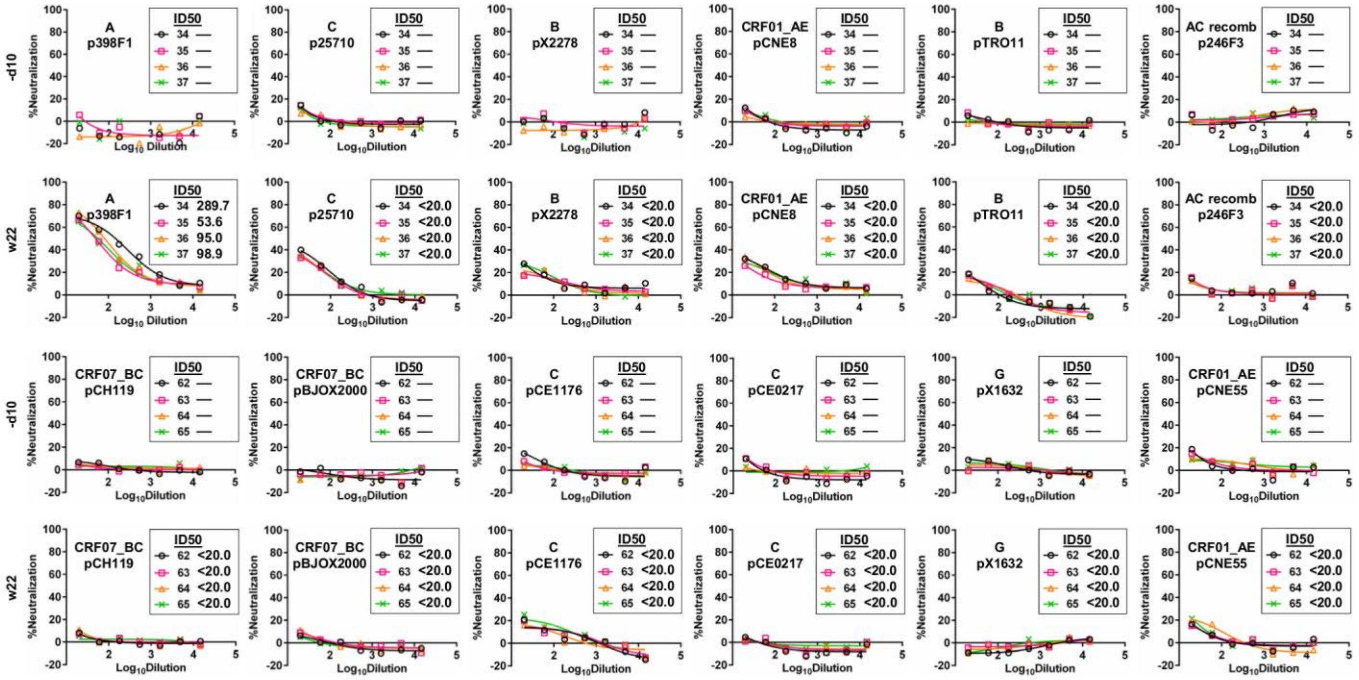

Fig. S7. B cell activation and in vivo evaluation of $gp41_{ECTO}$-stabilized trimers and nanoparticles. (**A**) Ca^{2+} mobilization in B cell transfectants carrying PGT145, PGT121, and VRC01 bNAb receptors. WEHI231 cells expressing a doxycyclin-inducible form of bNAb B cell receptor (BCR) were stimulated with anti-BCR antibodies or the indicated antigens at a concentration of 10 μg ml^{-1}: anti-human Ig κ-chain $F(ab')_2$; anti-mouse IgM; an UFO-BG trimer derived from a clade A, B, C, B/C, or A/E strain, or a gp140-PADRE construct containing a redesigned HR1 bend within $gp41_{ECTO}$. (**B**) Testing $gp41_{ECTO}$-stabilized BG505 trimer and nanoparticle immunogens in mice and ELISA binding of purified mouse IgGs to three HIV-1 antigens, including a BG505 UFO trimer, an N332 supersite probe based on ferritin nanoparticle (1GUT_A_ES-FR) or I3-01 nanoparticle (1KIG_L_ES-2-I3-01), and a V1V2 apex probe based on ferritin nanoparticle (clade C V1V2-FR). EC_{50} values are labeled for all ELISA plots except for instances in which the highest OD_{450} value is below 0.1 or in cases of ambiguous data fitting. (**C**) HIV-1 neutralization by group-combined mouse IgGs, with IC_{50}s color-coded according to the value ranging from green (weak neutralization) to red (potent neutralization). (**D**) ELISA binding of rabbit plasma to BG505 UFO trimer and an I3-01 nanoparticle presenting 60 N332 scaffolds (1KIG_L_ES-2-I3-01). The heat-inactivated plasma was diluted by 50-fold and subjected to a 10-fold dilution series in the assay. EC_{50} binding antibody titers are labeled for all plots except for the time point of day -10 (-d10). (**E**) Neutralization of clade A tier 2 BG505.T332N and clade B tier 1 SF162 by rabbit plasma. The heat-inactivated plasma was diluted by 50-fold and subjected to a 3-fold dilution series in the TZM-bl assay. ID_{50} NAb titers are labeled for all plots except for the time points of day -10 (-d10) and week 1 (w1), as well as week 6 (w6) for the trimer group tested against BG505.T332N. In (D) and (E), samples obtained from eight rabbits in the trimer group (34, 35, 36, and 37) and the ferritin group (62, 63, 64, and 65) at the time points of day -10 (-d10) and weeks 1, 6, 14, 22, and 30 (w1, w6, w14, w22, and w30) were analyzed in ELISA and neutralization assays. (**F**) Neutralization of 12 isolates in the global panel by rabbit plasma from the trimer group. (**G**) Neutralization of 12 isolates in the global panel by rabbit plasma from the ferritin group. In (F) and (G), samples at day -10 (-d10) and week 22 (w22) were analyzed to examine plasma neutralization of heterologous HIV-1 clones. To increase the sensitivity of detection, rabbit plasma was diluted by 20-fold followed by a 3-fold dilution series in TZM-bl assays. To reduce false positives caused by non-specific antiviral proteins present in the rabbit plasma, a simple metric was utilized that directly compares the percent neutralization of the w22 sample to that of the -d10 sample from the same animal at the 20- and 60-fold dilutions. The w22 signals at both dilutions must be 2-fold greater than the corresponding -d10 signals for a sample to be considered neutralizing a virus clone in the global panel. By this metric, the w22 samples in both trimer and ferritin groups neutralized p398F1 (clade A), p25710 (clade C), pX2278 (clade B), and pCNE8 (clade A/E) among others. ID_{50} NAb titers are only labeled for p398F1, for which 60-80% neutralization was observed for w22 compared to -d10, approaching ID_{50} values observed for the autologous neutralization.

Table S1. X-ray data collection and refinement statistics.

**Data collection**	**H078.14 UFO-BG + Fab PGT124 + Fab 35O22**
Beamline	APS 23-IDB
Wavelength (Å)	1.0332
Detector	Eiger 16M
Space group	$P6_3$
Unit cell parameters (Å)	
a, b, c (Å)	127.3 127.3 316.3
Resolution (Å)	50.0-4.6 (4.68-4.60)[a]
Total reflections	82,828
Unique reflections	15,619 (798)
Redundancy	5.3 (5.2)[a]
Completeness (%)	96.9 (98.0)[a]
$<I/\sigma_i>$	7.7 (1.25)[a]
R_{sym}[b]	0.29 (>1.00)[a]
R_{pim}[c]	0.12 (0.76)[a]
$CC_{1/2}$[d]	0.76 (0.58)[a]
**Refinement statistics**	
Resolution (Å)	48.8-4.6 (4.9-4.6)[a]
Reflections (work)	14,840
Reflections (test)	749
R_{cryst}(%)[e]	31.2
R_{free}(%)[f]	34.0
Average B value (Å^2) (Proteins/Glycans)	272 / 323
**RMSD from ideal geometry**	
Bond length (Å)	0.003
Bond angles (°)	0.678
**Ramachandran statistics (%)**	
Favored	93.67
Allowed	5.85
Outliers	0.48
**PDB ID**	6CE0

[a]Numbers in parentheses are for highest resolution shell

[b]$R_{sym} = \Sigma_{hkl} \Sigma_i | I_{hkl,i} - <I_{hkl}> | / \Sigma_{hkl} \Sigma_i I_{hkl,i}$, where $I_{hkl,i}$ is the scaled intensity of the ith measurement of reflection h, k, l, and $< I_{hkl}>$ is the average intensity for that reflection

[c]$R_{pim} = \Sigma_{hkl} (1/(n-1))1/2 \Sigma_i | I_{hkl,i} - <I_{hkl}> | / \Sigma_{hkl} \Sigma_i I_{hkl,i}$, where n is the redundancy

[d]$CC_{1/2}$ = Pearson Correlation Coefficient between two random half datasets

[e]$R_{cryst} = \Sigma_{hkl} | Fo - Fc | / \Sigma_{hkl} | Fo | \times 100$

[f]R_{free} was calculated as for R_{cryst}, but on a test set comprising 5% of the data excluded from refinement
